# Homo- and hetero-dimeric sesquiterpenoids with unprecedented skeletons and their co-occurring monomers from* Vernonia solanifolia* with anti-liver steatotic potential

**DOI:** 10.1186/s13020-026-01418-9

**Published:** 2026-05-28

**Authors:** Yue Yang, Fei Zhou, Chang-Qiang Ke, Sheng Yao, Ligen Lin, Chunping Tang, Yang Ye

**Affiliations:** 1 State Key Laboratory of Drug Research, and Natural Products Chemistry Department, Shanghai Institute of Materia Medica, Chinese Academy of Sciences, Shanghai, 201203 China; 2 State Key Laboratory of Mechanism and Quality of Chinese Medicine, Institute of Chinese Medical Sciences, University of Macau, Taipa, Macau, 999078 China; 3 Zhongshan Institute for Drug Discovery, Shanghai Institute of Materia Medica, Chinese Academy of Sciences, Zhongshan, 528400 China; 4 China-Serbia “Belt and Road” Joint Laboratory for Natural Products and Drug Discovery, Shanghai Institute of Materia Medica, Chinese Academy of Sciences, Shanghai, 201203 China

**Keywords:** *Vernonia solanifolia*, Asteraceae, Dimeric sesquiterpenoids, Lipid-lowering activity, Liver steatosis

## Abstract

**Background:**

Sesquiterpenoids show potential as therapeutics against metabolic dysfunction-associated steatotic liver disease (MASLD). *Vernonia solanifolia* Benth. has been traditionally used to treat abdominal pain and enteritis. However, the anti-steatotic effect of *V. solanifolia* and its chemical principles have not been illustrated.

**Methods:**

The structures of new compounds were determined through extensive spectroscopic analysis including 1D/2D nuclear magnetic resonance (NMR), high-resolution electrospray ionization mass spectrometry, ultraviolet and infrared. Especially, the structure of compound **6** was further verified by single crystal X-ray diffraction, and the relative and absolute configurations of compounds **1**–**5** and **7**–**9** were resolved with the help from density-functional theory-NMR and time-dependent density-functional theory-electronic circular dichroism calculation. The lipid-lowering activity was evaluated on palmitic acid/oleic acid (P/O)-treated AML12 hepatocytes. The biological evaluation was performed using Nile Red staining, flow cytometry, commercial kits, and Western blotting.

**Results:**

Nine new sesquiterpenoid dimers and their co-occurring monomers (**1**–**9**) were identified from the leaves of *V. solanifolia*. A unique 10/5/6/5/7 pentacyclic ring system is exemplified in compounds **1** and** 2**. These compounds were constructed from a germacranolide and a 4,5-*seco*-guaianolide monomer through an unprecedented spiro cyclohexene ring. The first examples of germacrane-guaiane and guaiane-xanthane heterogeneous dimers are shown in compounds **3** and **4**, respectively, which also possess a spiro cyclohexene ring linkage. A guaianolide dimer featuring a rare C-13/C-13ʹ linkage is exhibited in compound **5**. The plausible biosynthetic pathways for compounds **1** and **5** were proposed. The isolates were tested for their lipid-lowering effect on P/O-treated AML12 hepatocytes, and the results showed that compound **3** significantly reduced lipid content in hepatocytes by triggering the AMP-activated protein kinase/acetyl-CoA carboxylase/peroxisome proliferator-activated receptor *γ* coactivator-1*α* signaling pathway.

**Conclusion:**

Five homo- and hetero-dimeric sesquiterpenoids with unprecedented skeletons were characterized from *V. solanifolia*, together with four new co-occurring monomers. It’s the first time to report the lipid-lowering activity of sesquiterpenoids from this genus, which might be developed as lead compounds against MASLD.

**Supplementary Information:**

The online version contains supplementary material available at 10.1186/s13020-026-01418-9.

## Introduction

Chinese medicines-derived compounds have historically served as an invaluable source of structurally diverse for drug discovery [1]. 63.1% FDA-approved small-molecule drugs from 1981 to 2019 are natural origins, either as unmodified natural products or as derivatives inspired by their pharmacophoric features, such as paclitaxel, artemisinin and lovastatin [2]. This enduring relevance stems from the unique structural complexity of Chinese medicines-derived compounds, which have evolved under selective pressure to interact with biological targets. Sesquiterpenoid oligomers are a distinctive category of natural products, produced via biogenetic assembly of at least two sesquiterpenoid units through diverse linkage patterns [3]. In recent years, sesquiterpenoid oligomers have attracted considerable attention due to their diverse skeletons and intriguing pharmacological activities [4–6]. Our group has continuously devoted to identify a series of sesquiterpenoid dimers, trimers, and even tetramers from plants of the Asteraceae family, which exhibited remarkable anti-inflammatory or cytotoxic activities [7–9].

The genus *Vernonia*, belonging to the Asteraceae family, is one of the largest genera of flowering plants, comprising approximately 1000 species distributed across the tropical and temperate regions of Africa, Asia and America. Over 100 species of *Vernonia* are utilized in folk medicine to treat various diseases, such as malaria, infections, fever, worms, wounds, and diabetes [10]. The primary secondary metabolites of the *Vernonia* genus are sesquiterpenoids, including germacranolides [11, 12], elemanolides [13], guaianolides [14], and eudesmanolides [15], exhibiting a broad spectrum of pharmacological activities, such as anti-tumor [16], anti-inflammation [17], anti-virulence [15], and anti-trypanosomatid [18]. Till now, only a few sesquiterpenoid dimers were found from plants of the *Vernonia* genus [19–21].

**Fig. 1:**
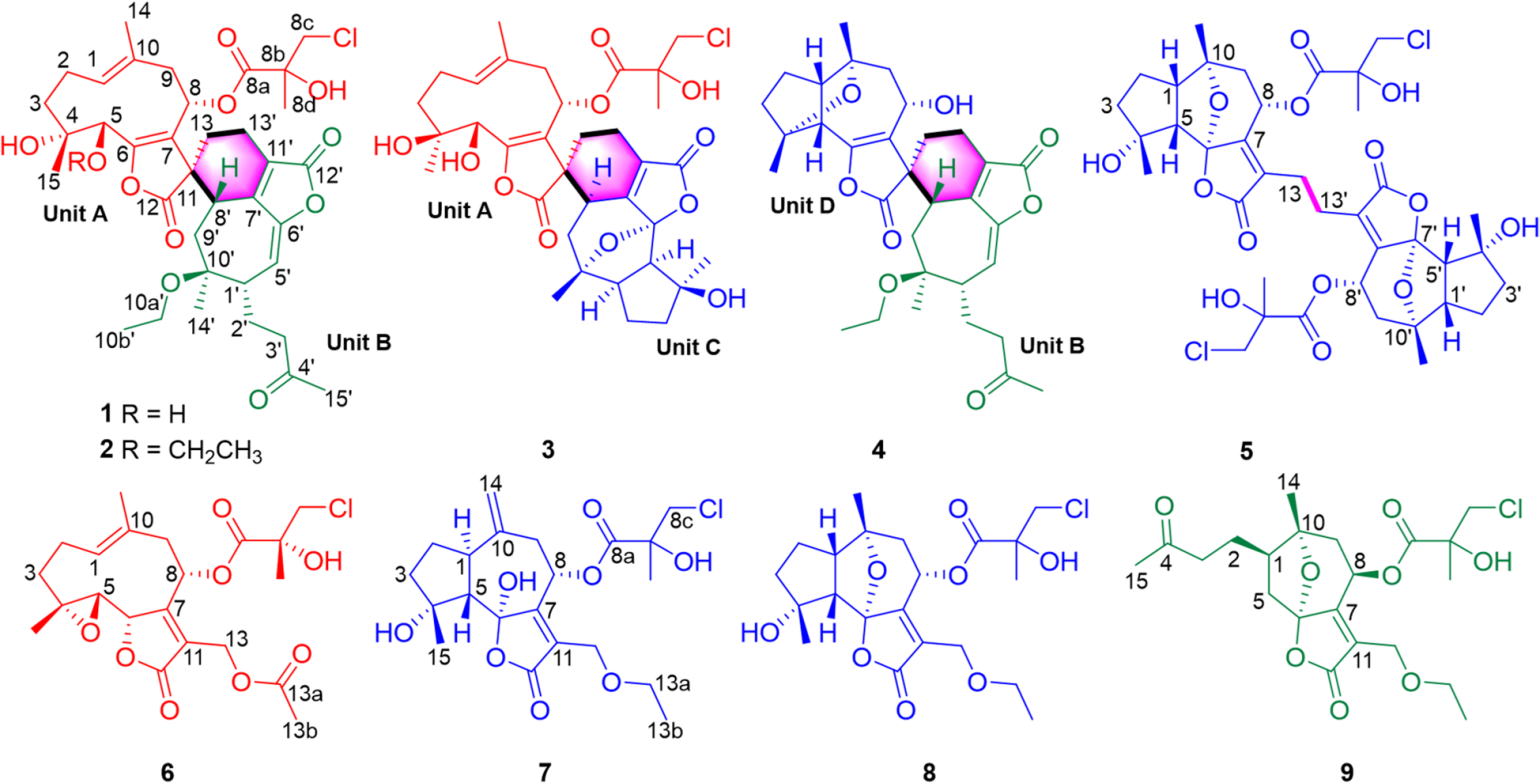
Structures of new compounds

*Vernonia solanifolia* Benth. is a species widely distributed in southern China, India, Myanmar, Vietnam, Laos, and Cambodia. It has been traditionally used to treat abdominal pain and enteritis. Our previous study discovered the existence of an array of bisabolane-type sesquiterpenoids with anti-inflammatory property [22]. During ongoing efforts in searching for novel structures to treat liver steatosis, nine previously undescribed sesquiterpenoids were isolated and characterized from the leaves of *V. solanifolia*, including five dimers (diversolanolides A–E, **1**–**5**) and four monomers (versolanolides A–D, **6**–**9**) (Fig. 1). Specifically, compounds **1**–**4** feature three new carbon scaffolds that incorporate two distinct monomeric units through a rare spiro cyclohexene ring, while compound **5** represents the first guaiane-type sesquiterpenoid dimer with a unique C-13/C-13' linkage. All the isolates were tested for their lipid-lowering effect on palmitic acid/oleic acid (P/O)-treated AML12 hepatocytes. In this study, we describe the isolation, structure characterization, plausible biogenetic pathways, and the anti-liver steatotic potential of these compounds.

## Materials and methods

### General experimental procedures

The optical rotations were measured on a Rudolph Research Analytical Autopol VI automatic polarimeter. Infrared (IR) spectra were obtained on a Nicolet Magna FRIR-750 spectrometer. Ultraviolet (UV) spectra were recorded on a Varian Cary 300 Bio spectrophotometer. Electronic circular dichroism (ECD) spectra were measured on a JACSO J-815 spectrometer. High-resolution electrospray ionization mass spectrometry (HRESIMS) data were collected on a Waters Synapt G2-Si Q-Tof mass spectrometer. 1D and 2D nuclear magnetic resonance (NMR) spectra were recorded using a BrukerAvance III-500 or BrukerAvance III-600 spectrometers and an Agilent DD2-600 spectrometer. The chemical shift (*δ*) values were reported in ppm, the residual signals of dimethyl sulfoxide and CHCl_3_ were used as internal standards, and the coupling constants (*J*) are in hertz. LCESIMS analysis was performed on a Waters 2695 instrument with a 2998 PDA detector equipped with a Waters Acquity evaporative light scattering detector (ELSD), and a Waters 3100 SQDMS detector. Preparative high-pressure liquid chromatography (HPLC) was run on a Varian PrepStar system with an Alltech 3300 ELSD with a Waters Sunfire RP C_18_, 5 μm, 30 × 150 mm column. MCI gel CHP20P (75–150 μm, Mitsubishi Chemical Industries, Tokyo, Japan), silica gel (100–200, 200–300, and 300–400 mesh, Qingdao Marine Chemical Industrials, Qingdao, China), ODS gel AAG12S50 (12 nm, S-50 μm, YMC, Japan) and Sephadex LH-20 (Pharmacia Biotech AB, Uppsala, Sweden) were used for column chromatography. Thin-layer chromatography (TLC) was carried out on precoated silica gel GF254 plates (Yantai Chemical Industrials, Yantai, China).

### Plant material

The leaves of *V*. *solanifolia* were collected from Yunnan Province, China, in 2014, and identified by Professor Jingui Shen from Shanghai Institute of Materia Medica. A voucher specimen (no. 20140610) was deposited at the Herbarium of Shanghai Institute of Materia Medica, Chinese Academy of Sciences.

### Extraction and isolation

The leaves of *V*. *solanifolia* (5 kg) were extracted with 95% EtOH (3 × 20 L) at room temperature (3 days each). After evaporation of the EtOH percolates, 500 g crude residue was obtained. The residue was suspended in water and partitioned successively with petroleum ether, CH_2_Cl_2_, and EtOAc. The CH_2_Cl_2_ extract (21 g) was subjected to a Sephadex LH-20 column (CHCl_3_/MeOH) to give four fractions Fr. 1–Fr. 4. Fr. 3 was chromatographed on an MCI column eluted with aqueous MeOH in a step manner (20, 30, 35, 40, 45, 50, 55, 60, 65, 70, 75, 80, and 100%), and finally acetone, affording 16 fractions (Fr. 3A–Fr. 3P). Fr. 3A was recrystallized to yield compound **6** (200 mg, *t*_R_ = 15.94 min). Subfractions Fr. 3H1–3H7 were yielded from Fr. 3H by using a Sephadex LH-20 column eluted with MeOH. Fr. 3H3 was further separated into 13 sub-fractions (3H3A–3H3M) by a silica gel column (300–400 mesh) eluted with a gradient solvent system of petroleum ether/acetone (50:1, 40:1, 30:1, 20:1, 10:1, and 5:1). Compound **8** (3 mg, *t*_R_ = 14.22 min) was obtained from Fr. 3H3D by preparative HPLC using MeCN/H_2_O as the mobile phase (0–80 min, from 26 to 56%). Fr. 3J was subjected to column chromatography over Sephadex LH-20 (eluted with MeOH) to get seven subfractions 3J1–3J7. Compounds **7** (3 mg, *t*_R_ = 16.31 min) and **9** (4 mg, *t*_R_ = 15.35 min) were isolated from Fr. 3J3 by column chromatography over silica gel (300–400 mesh) and preparative HPLC (MeCN/H_2_O: 0–80 min, from 30 to 60%). Fr. 3K was separated by a Sephadex LH-20 column (eluted with MeOH) to afford six subfractions 3K1–3K6. Fr. 3K2 was subjected to a silica gel column (300–400 mesh) eluted with a mixture of CHCl_3_/EtOH (500:1, 200:1, 100:1, 50:1, 20:1, and 10:1) to give 13 fractions (Fr. 3K2A–3K2M). Compound **1** (2 mg, *t*_R_ = 18.03 min) was isolated from Fr. 3K2D by preparative HPLC (MeCN/H_2_O: 0–80 min, from 38 to 68%). Fr. 3K2L was purified with preparative HPLC using MeCN/H_2_O (0–80 min, from 33 to 63%) to yield compounds **3** (4 mg, *t*_R_ = 16.22 min) and **5** (4 mg, *t*_R_ = 17.51 min). Fr. 3L was isolated by column chromatography over Sephadex LH-20 gel (eluted with MeOH) to afford six subfractions 3L1–3L6. Compound **2** (2 mg, *t*_R_ = 20.25 min) was isolated from Fr. 3L2 by a silica gel column (300–400 mesh) and preparative HPLC using MeCN/H_2_O (0–80 min, from 48 to 78%). Compound **4** (1.5 mg, *t*_R_ = 18.21 min) was obtained from Fr. 3L3 by silica gel column chromatography (300–400 mesh) and preparative HPLC using MeCN/H_2_O (0–80 min, from 40 to 70%). The Retention Time (t_*R*_) of compounds were collected on LCESIMS (MeCN/H_2_O, 0–25 min, from 5 to 95%).

*Diversolanolide A (****1****):* White powder; [*α*]^20^_D_ + 55 (*c* 0.8, MeOH); UV (MeOH) *λ*_max_ (log *ε*) 205 (3.85), 285 (3.92) nm; IR (KBr)* ν*_max_ 3469, 2960, 2918, 2849, 1744, 1371, 1265, 1173, 1089 cm^−1^; ^1^H and ^13^C data, see Table 1; HRESIMS *m/z* 708.3165 [M + NH_4_]^+^ (calcd for C_36_H_51_NO_11_Cl, 708.3151).

**Table 1:** ^1^H (600 MHz) and ^13^C NMR (125 MHz) data of compounds **1**–**4** in CDCl_3_

No	**1**		**2**		**3**		**4**	
	*δ*_H_ (*J* in Hz)	*δ*_C_	*δ*_H_ (*J* in Hz)	*δ*_C_	*δ*_H_ (*J* in Hz)	*δ*_C_	*δ*_H_ (*J* in Hz)	*δ*_C_
1	4.86, d (11.5)	134.8	4.93, d (11.4)	135.6	4.91, d (11.7)	136.0	2.91, d (3.1)	49.5
2	2.28, q (12.8); 2.18, m	24.1	2.23, m; 2.14, m	24.0	2.71, m; 2.02, m	23.0	1.94, m; 1.65, m	25.0
3	2.06, m; 1.67, t (13.4)	41.8	1.94, m; 1.71, t (13.4)	40.4	1.95, dd (14.8, 6.5); 1.56, m	40.1	*α* 1.74, m; *β* 1.67, m	35.3
4		75.7		75.0		75.5		89.0
5	3.73, d (10.4)	69.1	3.49, s	76.3	4.61, s	72.0	2.56, s	52.9
6		153.4		150.6		153.8		151.5
7		112.8		115.4		118.0		121.3
8	5.69, dd (11.4, 2.9)	71.1	5.61, d (11.6)	71.6	5.90, dd (4.7, 2.1)	70.3	4.44, t (5.2)	62.8
9	2.48, m; 2.17, m	43.9	2.39, m; 2.30, m	43.5	2.63, m; 2.14, d (14.0)	42.6	2.13, m; 2.09, m	51.0
10		127.1		126.1		126.4		81.1
11		51.5		52.4		51.2		52.1
12		174.7		175.0		178.3		176.6
13	2.42, m; 2.19, m	28.2	2.50, m; 2.22, m	29.3	2.21, dt (14.1, 8.5); 2.02, m	30.4	2.19, m; 1.94, m	29.1
14	1.81, s	16.7	1.82, s	16.7	1.89, s	19.7	1.34, s	28.0
15	1.38, s	24.6	1.39, s	26.4	1.43, s	25.4	1.28, s	18.5
5a			3.68, dp (8.8, 7.0); 3.42, m	65.7				
5b			1.24, t (7.0)	15.2				
8a		172.3		172.8		171.2		
8b		75.2		75.7		75.3		
8c	3.86, m; 3.64, d (11.3)	51.1	3.95, d (11.1); 3.60, d (11.1)	51.6	3.89, d (11.2); 3.62, d (11.2)	51.6		
8d	1.51, s	24.1	1.51, s	24.0	1.55, s	24.0		
4-OH			2.82, s					
5-OH	3.58, d (10.4)							
8b-OH	3.77, s		4.10, s					
1′	2.53, m	43.1	2.68, m	42.2	2.39, m	51.8	2.60, m	43.0
2′	2.03, m; 1.52, m	25.4	1.97, m; 1.36, m	27.0	1.80, m; 1.58, m	23.6	2.06, m; 1.49, m	25.6
3′	2.58, m; 2.41, m	42.3	2.58, ddd (17.8, 8.3, 5.1); 2.46, m	41.5	1.65, m; 1.57, m	37.0	2.46, m	41.8
4′		208.5		208.2		85.1		208.2
5′	5.37, d (5.7)	111.3	5.53, d (6.4)	111.6	2.51, s	56.0	5.39, d (5.7)	111.3
6′		150.8		149.8		105.4		150.2
7′		151.1		150.0		161.4		151.6
8′	3.88, m	35.1	3.99, d (12.1)	36.1	3.48, m	38.7	3.42, m	33.1
9′	1.89, dd (14.5, 2.1); 1.23, m	35.3	1.78, dd (14.3, 3.8); 1.52, m	35.6	1.80, m; 1.51, m	36.4	1.81, dd (14.5, 1.8); 1.49, m	36.1
10′		79.1		79.0		80.1		78.8
11′		127.2		127.8		126.2		127.0
12′		168.5		168.4		170.1		168.5
13′	2.82, m; 2.59, m	16.9	2.77, m; 2.64, dd (18.9, 6.2)	17.3	2.69, m; 2.57, m	18.0	2.76, m; 2.49, m	16.5
14′	1.12, s	22.5	1.22, s	24.9	1.29, s	27.3	1.21, s	23.7
15′	2.15, s	30.2	2.15, s	30.2	1.19, s	17.4	2.15, s	30.2
10a′	3.41, m; 3.31, dd (8.8, 6.6)	57.2	3.40, m	57.3			3.43, m	57.4
10b′	1.05, t (6.9)	16.3	1.08, t (7.0)	16.0			1.15, t (6.9)	15.9
4′-OH					3.93, s			

*Diversolanolide B (****2****):* White powder; [*α*]^20^_D_ + 61 (*c* 0.8, MeOH); UV (MeOH) *λ*_max_ (log *ε*) 230 (3.82), 285 (4.19) nm; IR (KBr)* ν*_max_ 3459, 2962, 2920, 2849, 1789, 1742, 1462, 1366, 1260, 1181, 1077 cm^−1^; ^1^H and ^13^C data, see Table 1; HRESIMS *m/z* 736.3485 [M + NH_4_]^+^ (calcd for C_38_H_55_NO_11_Cl, 736.3464).

*Diversolanolide C (****3****):* White powder; [*α*]^20^_D_ –46 (*c* 1.2, MeOH); UV (MeOH) *λ*_max_ (log *ε*) 205 (3.94) nm; IR (KBr)* ν*_max_ 3461, 2925, 2854, 1744, 1455, 1383, 1262, 1176, 1087 cm^−1^; ^1^H and ^13^C data, see Table 1; HRESIMS *m/z* 663.2578 [M + H]^+^ (calcd for C_34_H_44_O_11_Cl, 663.2572).

*Diversolanolide D (****4****):* White powder; [*α*]^20^_D_ + 42 (*c* 1.2, MeOH); UV (MeOH) *λ*_max_ (log *ε*) 280 (3.76) nm; IR (KBr)* ν*_max_ 3456, 2960, 2920, 2849, 1764, 1710, 1453, 1374, 1257, 1173, 1065 cm^−1^; ^1^H and ^13^C data, see Table 1; HRESIMS *m/z* 553.2798 [M + H]^+^ (calcd for C_32_H_41_O_8_, 553.2801).

*Diversolanolide E (****5****):* White powder; [*α*]^20^_D_ + 15 (*c* 0.8, MeOH); UV (MeOH) *λ*_max_ (log *ε*) 210 (4.11) nm; IR (KBr)* ν*_max_ 3451, 2965, 2923, 2854, 1742, 1455, 1376, 1260, 1176, 1109, 1072 cm^−1^; ^1^H and ^13^C data, see Table 2; HRESIMS *m/z* 816.2779 [M + NH_4_]^+^ (calcd for C_38_H_52_NO_14_Cl_2_, 816.2765).

**Table 2:** ^1^H and ^13^C NMR data of compounds **5**–**9**

No	**5**^a^		No	**6**^b^		**7**^a^		**8**^a^		**9**^a^	
	*δ*_H_ (*J* in Hz)	*δ*_C_		*δ*_H_ (*J* in Hz)	*δ*_C_	*δ*_H_ (*J* in Hz)	*δ*_C_	*δ*_H_ (*J* in Hz)	*δ*_C_	*δ*_H_ (*J* in Hz)	*δ*_C_
1/1′	2.58, d (3.2)	46.1	1	5.42, t (8.1)	127.3	3.02, td (11.9, 5.5)	46.7	2.60, d (3.1)	46.2	2.19, m	47.0
2/2′	*α* 1.88, d (10.5); *β* 1.64, m	24.3	2	2.28, m	22.0	1.96, m; 1.60, m	27.3	*α* 1.90, m; *β* 1.63, m	24.4	2.03, m; 1.61, m	23.6
3/3′	*α* 1.55, m; *β* 1.70, t (12.0)	36.7	3	*α* 1.25, m; *β* 2.10, m	35.0	1.96, m; 1.90, m	42.1	*α* 1.56, m; *β* 1.71, m	36.6	2.59, m; 2.46, m	42.8
4/4′		84.7	4		60.2		83.2		84.5		208.8
5/5′	2.36, s	58.8	5	2.46, d (8.9)	64.2	1.49, m	60.2	2.37, s	59.1	2.49, m; 2.11, dd (13.2, 6.9)	35.3
6/6′		104.7	6	4.96, dd (8.9, 1.1)	81.8		108.6		103.7		107.1
7/7′		158.0	7		162.6		158.6		162.1		155.2
8/8′	5.89, dd (10.2, 7.0)	69.5	8	5.20, dd (10.4, 3.4)	70.4	6.12, t (7.0)	68.8	5.83, dd (10.4, 7.6)	69.2	6.26, dd (4.5, 3.0)	64.4
9/9′	*α* 1.99, dd (13.8, 10.3); *β* 2.28, dd (13.8, 7.0)	44.8	9	2.74, m	43.0	2.84, d (7.1)	38.7	*α* 1.92, dd (13.8, 10.4); *β* 2.33, dd (13.8, 7.5)	44.9	2.14, m	38.3
10/10′		78.9	10		128.9		143.5		78.9		83.7
11/11′		127.1	11		127.5		130.9		124.9		125.3
12/12′		172.0	12		169.9		169.2		170.5		169.8
13/13′	2.64, m	22.7	13	5.08, d (13.0); 4.87, d (13.0)	55.3	4.25, s	62.9	4.48, d (12.2); 4.13, d (12.2)	60.7	4.32, d (13.8); 4.24, d (13.8)	63.1
14/14′	1.30, s	27.7	14	1.74, s	17.1	5.05, s; 5.00, s	114.0	1.30, s	27.7	1.39, s	25.9
15/15′	1.15, s	19.0	15	1.38, s	16.8	1.57, s	30.9	1.16, s	18.7	2.19, s	30.5
8a/8a′		173.7	8a		171.4		172.7		173.0		172.5
8b/8b′		75.5	8b		73.8		75.0		75.3		75.4
8c/8c′	3.89, d (11.3); 3.62, d (11.3)	50.9	8c	3.75, d (11.1); 3.69, d (11.1)	50.2	3.82, d (11.0); 3.57, d (11.0)	51.3	3.86, d (11.1); 3.59, m	51.5	3.91, d (11.3); 3.62, d (11.3)	51.4
8d/8d′	1.54, s	23.9	8d	1.40, s	22.7	1.48, s	23.6	1.53, s	23.6	1.50, s	23.7
1/1′	2.58, d (3.2)	46.1	13a		169.2	3.52, qt (6.6, 3.3)	67.4	3.60, m	67.1	3.53, q (7.0)	67.3
2/2′	*α* 1.88, d (10.5); *β* 1.64, m	24.3	13b	2.07, s	19.9	1.20, t (7.0)	15.1	1.20, t (7.0)	15.3	1.19, t (7.0)	15.2
			4-OH			2.46					
			6-OH			6.48					
			8b-OH			3.63					

^a^Data recorded at 600 MHz (^1^H) and 125 MHz (^13^C) in CDCl_3_. ^b^Data recorded at 600 MHz (^1^H) and 150 MHz (^13^C) in DMSO-*d*_6_

*Versolanolide A (****6****):* Colorless crystals (MeOH); mp 137–139 ℃; [*α*]^20^_D_ –51 (*c* 1.1, MeOH); UV (MeOH) *λ*_max_ (log *ε*) 205 (4.03) nm; IR (KBr)* ν*_max_ 3464, 2919, 2853, 1767, 1747, 1599, 1384, 1229, 1181, 1108, 1032 cm^−1^; ^1^H and ^13^C data, see Table 2; HRESIMS *m/z* 443.1465 [M + H]^+^ (calcd for C_21_H_28_O_8_Cl, 443.1473).

*Versolanolide B (****7****):* White powder; [*α*]^20^_D_ + 13 (*c* 1.4, MeOH); UV (MeOH) *λ*_max_ (log *ε*) 205 (3.95) nm; IR (KBr)* ν*_max_ 3440, 2964, 2923, 2845, 1745, 1604, 1384, 1182, 1107 cm^−1^; ^1^H and ^13^C data, see Table 2; HRESIMS *m/z* 467.1445 [M + Na]^+^ (calcd for C_21_H_29_O_8_NaCl, 467.1449).

*Versolanolide C (****8****):* White powder; [*α*]^20^_D_ + 21 (*c* 0.7, MeOH); UV (MeOH) *λ*_max_ (log *ε*) 205 (3.89) nm; IR (KBr)* ν*_max_ 3445, 2918, 2850, 1748, 1595, 1384, 1181, 1110, 1075 cm^−1^; ^1^H and ^13^C data, see Table 2; HRESIMS *m/z* 467.1435 [M + Na]^+^ (calcd for C_21_H_29_O_8_NaCl, 467.1449).

*Versolanolide D (****9****):* White powder; [*α*]^20^_D_ + 41 (*c* 1.2, MeOH); UV (MeOH) *λ*_max_ (log *ε*) 210 (4.05) nm; IR (KBr)* ν*_max_ 3445, 2958, 2922, 2871, 1780, 1747, 1714, 1607, 1455, 1417, 1379, 1361, 1260, 1180, 1144, 1106, 1017, 973 cm^−1^; ^1^H and ^13^C data, see Table 2; HRESIMS *m/z* 467.1438 [M + Na]^+^ (calcd for C_21_H_29_O_8_NaCl, 467.1449).

### X-ray crystallographic analysis of compound 6

Crystals were obtained from methanol solution, and a suitable crystal was selected and mounted on a Bruker APEX-II CCD diffractometer. Using Olex2, the structure was solved with the olex2.solve structure solution program using Charge Flipping and refined with the ShelXL refinement package using Least Squares minimisation. Crystal data for compound **6**. The crystal was kept at 100.0 K during data collection. C_21_H_27_ClO_8_, *M* = 442.87 g/mol, orthorhombic, *a* = 8.2250(2) Å, *b* = 10.8921(3) Å, *c* = 23.6848(6) Å, space group P2_1_2_1_2_1_ (no. 19), *α* = 90°, *β* = 90°, *γ* = 90°, V = 2121.86(9) Å^3^, *Z* = 4,* T* = 100 K, *μ*(Cu K*α*) = 1.994 mm^−1^, 9887 reflections measured (7.464° ≤ 2*σ* ≤ 127.25°), 3310 unique (*R*_int_ = 0.0405, *R*_sigma_ = 0.0406), which were used in all calculations. The final *R*_1_ was 0.0282 (*I* > 2*σ*(*I*)) and *wR*_2_ was 0.0709. Flack parameter: 0.039(8). Crystallographic data for **6** have been deposited at the Cambridge Crystallographic Data Centre as deposit no. CCDC 2164554. Copies of the data can be obtained free of charge via the internet at www.ccdc.cam.ac.uk/conts/retrieving.html.

### Computational calculation

Density-functional theory (DFT) NMR and time-dependent DFT (TDDFT) ECD calculations were performed using the Gaussian 16 program [23]. The conformational search was carried out by the Conflex 8.0 software [24] using the MMFF force field within an energy window of 5.0 kcal/mol. The conformers with the Boltzmann population above 1.0% were reoptimized. DFT NMR calculations were run at the level of mPW1PW91/6-311G(d,p) with PCM model for chloroform and used the improved statistical method DP4 + [25] to perform the statistical analysis on the calculated and experimental values. TDDFT ECD calculations were run at the M06-2X/def2TZVP level with the SMD solvent model for methanol. ECD spectra were generated using the SpecDis software [26, 27].

### Cell culture

AML12 cells were obtained from American Type Culture Collection (Rockville, MD, USA) and cultured in Dulbecco’s Modified Eagle Medium (DMEM, Gibco, Gaithersburg, MD, USA) supplemented with 10% fetal bovine serum (FBS, Gibco) and ITS-G (10 μg/mL insulin, 5 mg/L transferrin, 5 μg/L selenous acid, Peiyuan Biotechnology, Shanghai, China) in humidified incubator containing 5% CO_2_ at 37 ℃. Oleic acid and palmitic acid (Sigma-Aldrich, St. Louis, MO, USA) were completely dissolved in 75% (*v*/*v*) ethanol by heating at 55 ℃ and then diluted in DMEM containing 1% (*w*/*v*) fatty acid-free bovine serum albumin (Sigma-Aldrich) to 500 and 250 μM, respectively. The solution was placed in a shaker incubator for 2 h and then sterilized by passing through 0.2 μm filters to obtain a P/O solution. AML12 cells were treated with the P/O solution for 24 h to induce lipid accumulation as described previously [28, 29].

### Cell viability

AML12 cell viability was evaluated by a colorimetric [3-(4,5-dimethylthiazol-2-yl)−2,5-diphenyltetrazolium bromide] (MTT) method as described previously [30]. In brief, AML12 cells (1 × 10^4^ cells/well) were seeded into 96-well plates and cultured for 24 h. The cells were treated with the indicated concentration (40, 20, 10, 5, 2.5, and 1.25 μM) of compounds **1**‒**9** for 24 h. Then, 1 mg/mL MTT solution was added into each well. After incubated for 4 h, 150 μL dimethyl sulfoxide (DMSO) was added to solubilize formazan precipitates. Finally, the absorption was measured at 570 nm using a microplate reader (FlexStation 3, Molecular Devices, CA, USA).

### Nile red staining

Nile red staining was performed as described previously [31]. In brief, AML12 hepatocytes were fixed with 10% formaldehyde solution and stained with Nile red (1 μg/mL, Sigma-Aldrich). After incubating for 30 min at 4 ℃ and then washing with phosphate-buffered saline (PBS), the fluorescence intensity was measured using the microplate reader with excitation and emission wavelength at 530 and 590 nm, respectively. DMSO was used as a blank control, and honokiol (HK) was used as a positive control [28]. The AML-12 cells were counterstained with 4′,6-diamidino-2-phenylindole (DAPI) for 10 min and the fluorescent images were captured by a Leica TCS SP8 fluorescence microscopy (Wetzlar Germany).

### Measurement of TG and T-CHO

Total glycerides (TG) and total cholesterol (T-CHO) in cell lysate were detected by using commercial kits (Nanjing Jiancheng, Nanjing, Jiangsu, China). TG and T-CHO levels were further normalized by the protein concentration.

### Mitochondrial membrane potential

JC-1 dye (Beyotime Biotechnology, Shanghai, China) was used to examine mitochondrial membrane potential of AML-12 cells as described previously [32]. Briefly, AML12 cells were seeded into 6-well microplates. After treated with P/O for 24 h, the cells were treated with 10 μM compound **3** or 10 μM FCCP (carbonyl cyanide-*p*-trifluoromethoxyphenylhydrazone, Sigma-Aldrich) for 24 h. Subsequently, the cells were washed with PBS and then incubated with JC-1 solution (10 µg/mL) for 20 min at 37 ℃. After washed with PBS twice, the fluorescent images were captured with a DMI8 microscope. In addition, ImageJ software (National Institutes of Health, Bethesda, MD, USA) was used to analyze fluorescence intensity.

### Western blotting analysis

AML-12 cells were treated with P/O solution for 24 h followed by treatment of different concentrations of **3** (5, 10, or 20 μM) for 24 h. After washed with pre-cooling PBS, the cells were lysed in RIPA buffer containing EDTA and protein phosphatase inhibitors. Equal amounts of protein (20 µg) were separated via SDS–PAGE and transferred onto 0.45 µm PVDF membranes, where they were stained with primary antibodies (anti-GAPDH, anti-p-AMPK, anti-AMPK, anti-p-ACC, anti-ACC, and anti-PGC-1*α*, Cell signaling, Danvers, MA, USA) overnight after blocked for 2 h in TBST containing 5% non-fat dried milk. Then, the membranes were probed with an HRP-conjugated secondary antibody (Cell Signaling) for 1 h at room temperature after washed with TBST. The bands were visualized using the ChemiDoc MP Imaging System (Bio-Rad, Hercules, CA, USA).

### Statistical analysis

All experiments were performed at least three biological replicates. Data are presented as mean ± standard deviation (SD) and the significant differences among multiple groups were analyzed with one-way ANOVA followed by Tukey’s post-hoc test. Data analysis was performed using GraphPad Prism5.0 (GraphPad Software, Inc., San Diego, CA, US). *P* < 0.05 was considered as significant difference.

## Results and discussion

### Structure elucidation of new compounds

Compound **1** was obtained as a white amorphous powder. Its molecular formula was determined as C_36_H_47_ClO_11_ by HRESIMS, with pseudo-molecular ion peaks at *m/z* 708.3165 and 710.3149 in a ratio of 3:1, indicative of one chlorine atom. IR absorptions at 3469 and 1744 cm^−1^ indicated the presence of hydroxy and carbonyl groups, respectively. The ^13^C NMR and DEPT-135 spectra (Table 1) of **1** displayed 36 carbon resonances, comprising six methyls, 10 methylenes, six methines, and 14 quaternary carbons. These included one ketone carbonyl (*δ*_C_ 208.5), three ester carbonyls (*δ*_C_ 174.7, 172.3, 168.5), and eight olefinic carbons (*δ*_C_ 153.4, 151.1, 150.8, 134.8, 127.2, 127.1, 112.8, 111.3). The ^1^H NMR and HSQC spectra of **1** showed signals for two olefinic protons at *δ*_H_ 5.37 (d, *J* = 5.7 Hz) and 4.86 (d, *J* = 11.5 Hz), two oxygenated methines at *δ*_H_ 5.69 (dd, *J* = 11.4, 2.9 Hz) and 3.73 (d, *J* = 10.4 Hz), five singlet methyls at *δ*_H_ 2.15, 1.81, 1.51, 1.38, and 1.12, and a triplet methyl at *δ*_H_ 1.05 (*J* = 6.9 Hz).

**Fig. 2:**
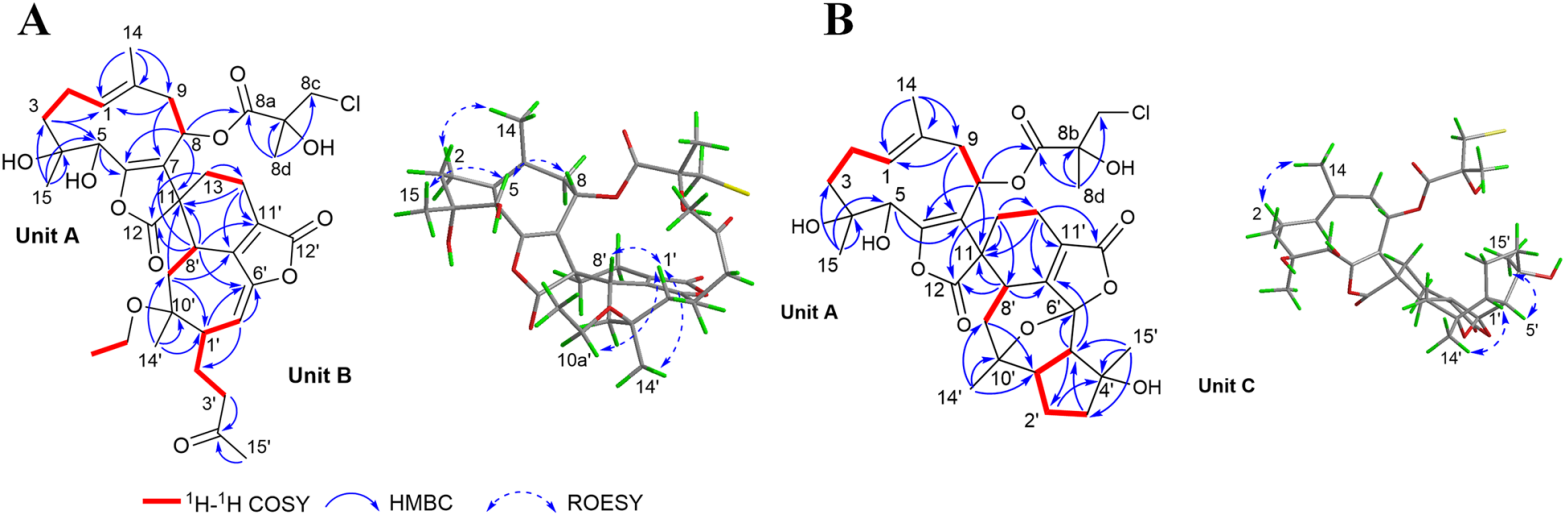
Key 2D NMR correlations of compounds **1** (**A**) and **3** (**B**)

The spin systems of H-1/H_2_-2/H_2_-3 and H-8/H_2_-9 in the ^1^H–^1^H COSY spectrum, together with HMBC correlations from H_2_-3 to C-1/C-5, from H_3_-15 to C-3/C-4/C-5, from H-5 to C-6, from H-8 to C-6/C-11, from H_2_-9 to C-1/C-7, from H_3_-14 to C-1/C-9/C-10, and from H_2_-13 to C-11, established a germarane-type sesquiterpene moiety (unit A, Fig. 2A). In parallel, the spin systems of H_2_-3′/H_2_-2′/H-1′/H-5′, and H-8′/H_2_-9′ in the ^1^H–^1^H COSY spectrum, together with HMBC correlations from H-1′ to C-6′, from H_2_-3′ and H_3_-15′ to C-4′, from H-5′ to C-6′, from H-8′ to C-6′/C-11′, from H_2_-9′ to C-1′/C-7′, from H_2_-13′ to C-7′/C-12′, and from H_3_-14′ to C-1′/C-9′/C-10′ established a 4,5-*seco*-guaiane moiety (unit B, Fig. 2A). The connectivity of units A and B was established by ^1^H–^1^H COSY correlations between H_2_-13 and H_2_-13′, and HMBC correlations from H_2_-13 to C-11/C-13′, from H_2_-13′ to C-11/C-7′/C-11′, and from H-8′ to C-11/C-12/C-11′, indicative of an unusual cyclohexene ring linking the two units (Fig. 2A).

The remaining signals were further assigned to two substituents: a 3-chloro-2-hydroxy-2-methylpropanoyloxy group at C-8 of unit A was established by the HMBC correlations from H-8 (*δ*_H_ 5.69, dd, *J* = 11.4 and 2.9 Hz) to C-8a (*δ*_C_ 172.3), and from H_3_-8d (*δ*_H_ 1.51, s) to C-8a/C-8b (*δ*_C_ 75.2)/C-8c (*δ*_C_ 51.1); and an ethoxy group at C-10′ of unit B was established by ^1^H–^1^H COSY correlations between H_2_-10a′ (*δ*_H_ 3.41, m; 3.31, dd, *J* = 8.8 and 6.6 Hz) and H_3_-10b′ (*δ*_H_ 1.05, t, *J* = 6.9 Hz), together with an HMBC correlation from H_2_-10a′ to C-10′ (*δ*_C_ 79.1) (Fig. 2A). Thus, compound **1** was determined to be a sesquiterpenoid dimer of a germacrene and a *seco*-guaiane unit, featuring a unique spiro cyclohexene linkage, and was named diversolanolide A.

Compound **2** was assigned a molecular formula of C_38_H_51_ClO_11_ by HRESIMS (*m*/*z* 736.3485 [M + NH_4_]^+^, calcd for 736.3464; the observed isotopic peak ratio of 3:1 at *m*/*z* 736.3485 and 738.3479 confirmed the presence of one chlorine atom), corresponding to 13 degrees of unsaturation. The ^1^H and ^13^C NMR spectra of **2** closely resembled those of **1**. Detailed comparison of the NMR data (Table 1) revealed chemical shift differences at C-5 (**2**: *δ*_C_ 76.3; **1**: *δ*_C_ 69.1), C-6 (**2**: *δ*_C_ 150.6;** 1**: *δ*_C_ 153.4) and C-7 (**2**: *δ*_C_ 115.4; **1**: *δ*_C_ 112.8), along with an additional ethoxy group [*δ*_H_ 3.68 (dq, *J* = 8.8, 7.0 Hz), 3.42 (m), *δ*_C_ 65.7; *δ*_H_ 1.24 (t, *J* = 7.0 Hz), *δ*_C_ 15.2] in **2**. The molecular weight of **2** was 28 Da higher than that of **1**, indicating that the 5-hydroxy group in **1** was replaced by an ethoxy group in **2**. The ^1^H–^1^H COSY correlation between H_2_-5a and H_3_-5b, together with the HMBC correlation from H_2_-5a [*δ*_H_ 3.68 (dq, *J* = 8.8, 7.0 Hz), 3.42 (m)] to C-5 (*δ*_C_ 76.3), supported this assignment (Fig. S1). Thus, compound **2** was defined as a 5-*O*-ethyl derivative of **1**, and named diversolanolide B.

Compound **3** was isolated as a white amorphous powder. The HRESIMS of **3** indicated a molecular formula of C_34_H_43_ClO_11_ (*m*/*z* 663.2578 [M + H]^+^, calcd for 663.2572; the characteristic isotopic ratio of 3:1 observed at *m*/*z* 663.2578 and 665.2570 confirmed the presence of one chlorine atom), corresponding to 13 degrees of unsaturation. IR absorptions at 3461 and 1744 cm^−1^ indicated the presence of hydroxy and carbonyl groups, respectively. The ^13^C NMR and DEPT-135 spectra of **3** displayed 34 carbon resonances, ascribed to five methyls, nine methylenes, six methines, and 14 quaternary carbons, including three ester carboxyl carbons (*δ*_C_ 178.3, 171.2, 170.1) and six olefinic carbons (*δ*_C_ 161.4, 153.8, 136.0, 126.4, 126.2, 118.0). The ^1^H NMR spectrum of **3** showed one olefinic proton (*δ*_H_ 4.91, d, *J* = 11.7 Hz), two oxygenated methines (*δ*_H_ 5.90, dd, *J* = 4.7, 2.1 Hz; *δ*_H_ 4.61, s), and five singlet methyls (*δ*_H_ 1.89, 1.55, 1.43, 1.29 and 1.19). Based on the degree of unsaturation, **3** should contain seven rings.

Detailed comparison of the ^1^H and ^13^C NMR data of **1** and **3** (Table 1) unveiled that both compounds shared the same germacranolide moiety (unit A). In addition, spin systems of H-5′/H-1′/H_2_-2′/H_2_-3′ and H-8′/H_2_-9′ in the ^1^H–^1^H COSY spectrum, together with HMBC correlations from H_3_-15′ to C-3′/C-4′/C-5′, from H-1′ to C-6′, from H-5′ to C-2′/C-6′/C-7′, from H-8′ to C-7′/C-9′, from H_3_-14′ to C-1′/C-9′/C-10′, and from H_2_-13′ to C-7′/C-11′/C-12′, established a guaianolide moiety (unit C, Fig. 2B). The connection between units A and C was deduced from the ^1^H–^1^H COSY correlations between H_2_-13 and H_2_-13′, as well as the HMBC correlations from H_2_-13 to C-11/C-8′/C-13′, from H_2_-13′ with C-11/C-7′/C-11′, and from H-8′ with C-11/C-12/C-7′, indicating the formation of a spiro cyclohexene ring at C-11 linking the two sesquiterpene lactone units (Fig. 2B). Given two degrees of unsaturation for unit A and three for unit C, plus one for the spiro cyclohexene ring, one additional ring was required to account for the total degree of unsaturation. Based on the chemical shifts (C-6′, *δ*_C_ 105.4; C-10′, *δ*_C_ 80.1) and HMBC correlations between C‑6′ and H‑1′ and H‑5′, an epoxide bridge between C-6′ and C-10′ was established. Therefore, the planar structure of **3** was fully elucidated, and named diversolanolide C, featuring a spiro cyclohexene ring linkage between a germacranolide and a guaianolide sesquiterpene moiety.

The molecular formula of **4** was designated as C_32_H_40_O_8_ by HRESIMS, corresponding to 13 indices of hydrogen deficiency. The ^13^C NMR and DEPT-135 spectra of **4** displayed 32 carbon resonances, comprising five methyls, nine methylenes, six methines, and 12 quaternary carbons. These included one ketone carbonyl (*δ*_C_ 208.2), two ester carbonyl (*δ*_C_ 176.6, 168.5), three sets of olefinic carbons (*δ*_C_ 151.6, 151.5, 150.2, 127.0, 121.3 and 111.3), one oxygenated methine (*δ*_C_ 62.8), and three oxygenated quaternary carbons (*δ*_C_ 89.0, 81.1 and 78.8). These data implied that compound **4** might also be a sesquiterpenoid dimer.

Detailed comparison of the NMR data of compounds **1** and **4** (Table 1) revealed that both compounds shared the same 4,5-*seco*-guaiane moiety (unit B). Additionally, spin systems of H-5/H-1/H_2_-2/H_2_-3 and H-8/H_2_-9 in the ^1^H–^1^H COSY spectrum, together with HMBC correlations from H_3_-15 (*δ*_H_ 1.28) to C-3 (*δ*_C_ 35.3), C-4 (*δ*_C_ 89.0) and C-5 (*δ*_C_ 52.9), from H-5 (*δ*_H_ 2.56) to C-2 (*δ*_C_ 25.0) and C-7 (*δ*_C_ 121.3), from H-8 (*δ*_H_ 4.44) to C-6 (*δ*_C_ 151.5) and C-10 (*δ*_C_ 81.1), from H_3_-14 (*δ*_H_ 1.34) to C-1 (*δ*_C_ 49.5), C-9 (*δ*_C_ 51.0), and C-10 (*δ*_C_ 81.1), and from H_2_-13 (*δ*_H_ 2.19, 1.94) to C-11 (*δ*_C_ 52.1) and C-12 (*δ*_C_ 176.6), established a guaianolide moiety (unit D, Fig. S1). The linkage between units B and C was defined also as a spiro cyclohexene ring, as deduced from ^1^H–^1^H COSY and HMBC correlations (Fig. S1). Based on the aforementioned data and the degree of unsaturation of **4**, an oxygen bridge between C-4 (*δ*_C_ 89.0) and C-10 (*δ*_C_ 81.1) was inferred. Accordingly, the planar structure of **4** was proposed, and named diversolanolide D.

Notably, all four dimeric sesquiterpenoids featured a spiro cyclohexene ring linkage connecting two distinct monomers. Compounds **1**–**3** shared the same germacranolide moiety (unit A), whereas **1**,** 2**, and** 4** contained the same *seco*-guaianolide fragment (unit B). Given the structural complexity of dimers **1**–**4**, a combined method employing ROESY/NOESY experiments (Fig. 2, S2), DFT NMR calculation, and TDDFT ECD calculation was applied to clarify the absolute configurations of **1**–**4**.

In the common germacranolide moiety (unit A) of** 1**–**3**, although ROESY/NOESY correlations of H_2_-2/H_3_-14 were observed for **1**–**3**, which defined *E*-configuration for the C1-C10 double bond, and those of H_3_-15/H-5 and H-5/H-8 for **1** and **2**, this evidence was insufficient for unambiguous assignment of the relative configurations of unit A due to the conformational flexibility of the 10-membered ring (Fig. 2A). Therefore, four truncated candidate structures (**A1**–**A4**, Fig. S3) with three chiral centers (C-4, C-5, and C-8) were constructed for DFT NMR calculations. DP4 + statistical analysis [25] of the theoretical and experimental data supported the specific structures of *rel*-(4*R*,5*R*,8*S*)-**A1** (Figs. S4 and S5) for compounds **1** and **2**, and *rel*-(4*R*,5*S*,8*R*)-**A3** (Fig. S6) for compound **3**.

In the common *seco*-guaianolide moiety (unit B) of **1**,** 2**, and** 4**, a NOESY/ROESY correlation between H-1′ and H-8′ placed these protons on the same face, whereas additional correlations of H-1′ with both H_3_-14′ and H_2_-10a′ rendered the relative configuration of H-1′ and H_3_-14′ ambiguous. Therefore, four candidate structures (**B1**–**B4**, Fig. S7) with two chiral centers (C-1′/C-8′, and C-10′), together with the spiro stereocenter C-11, were constructed to elucidate the relative configurations of unit B and the intermonomer spiro cyclohexene ring linkage via DFT NMR calculation. DP4 + statistical analysis of the theoretical and experimental data favored **B3** with *rel*-(11*R*, 1′*S*, 8′*S*, 10′*S*) configuration (Fig. S8–S10) for all three compounds, indicating a common relative configurations at C-11 and within the *seco*-guaianolide moiety.

Unit C of compound **3** and unit D of compound **4** were both guaianolide moieties. NOE correlations of H-5′/H_3_-15′ in unit C of **3** (Fig. 2B), together with the singlet nature of H-5′, placed H-1′, H-5′, and H_3_-15′ on the same face. The presence of an oxygen bridge between C-10′ and C-6′ restricted the conformational flexibility of the ring system. Accordingly, the NOE correlation between H-1′ and H_3_-14′ indicated that these protons were in different orientations. The relative configuration of C-8′ remained unassigned due to the absence of diagnostic NOE correlations involving H-8′. Therefore, to figure out the relative configuration of unit C and the spiro stereocenter C-11, DFT NMR calculations were performed on four candidate structures for unit C of **3** (Fig. S11). DP4 + statistical analysis of the theoretical and experimental data supported the *rel*-(11*R*, 1′*R*, 4′*S*, 5′*R*, 6′*S*, 8′*R*, 10′*R*)-**C3** (Fig. S12). Key NOESY correlations of H_3_-15/H-5, H-5/H-1, H-1/H_3_-14, and H-1/H-8 in unit D of **4** (Fig. S2), together with the singlet nature of H-5, established the relative configuration of unit D as depicted.

**Fig. 3:**
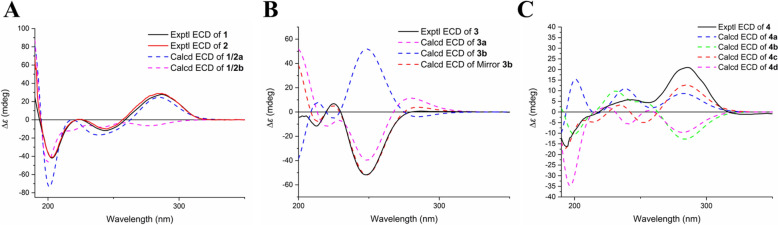
Comparison of experimental and calculated ECD spectra of **1** and **2** (**A**), **3** (**B**) and **4** (**C**)

After determining the relative configurations of each structural segment (units A–D) and the spiro cyclohexene ring linkage, possible whole structures were generated for **1**/**2** (two candidates, **1**/**2a** and **1**/**2b**, Fig. S13), **3** (two candidates, **3a** and **3b**, Fig. S14), and **4** (four candidates, **4a**–**4d**, Fig. S15). The additional two candidates for **4** (**4a** and **4b**) arose from the configurational variability at C-8. TDDFT ECD calculations were used to determine their absolute configurations. The calculated ECD spectra of **1/2a** (Fig. 3A), the mirror structure of **3b**, and **4c** (Fig. 3B, C) exhibited Cotton effects in good agreement with the respective experimental spectra. Therefore, the whole structures of sesquiterpenoid dimers **1**–**4** were proposed as shown.

The molecular formula of compound **5** was established as C_38_H_48_O_14_Cl_2_ by HRESIMS; the isotopic peaks at *m*/*z* 816.2779, 818.2753 and 820.2770 with an intensity ratio of 9:6:1 indicated the presence of two chlorine atoms. The ^1^H and ^13^C NMR data of **5** showed high similarities to those of **8** (Table 2). Considering the molecular formula, compound **5** was inferred to be a guaiane-type sesquiterpenoid dimer. The absence of an ethoxy group, together with the key HMBC correlations from the protons at *δ*_H_ 2.64 (H_2_-13/13′) to the carbons at *δ*_C_ 158.0 (C-7/7′), *δ*_C_ 127.1 (C-11/11′), *δ*_C_ 172.0 (C-12/12′) and *δ*_C_ 22.7 (C-13/13′) (Fig. S1), implied that two monomers might be connected through a C-13–C-13′ bond. The absolute configuration of **5** was defined to keep the same with that of **8** by the NOESY correlations and their similar Cotton effects in the ECD spectra (Fig. S16). Consequently, the structure of** 5** was fully elucidated, and named diversolanolide E, a guaiane-type sesquiterpenoid dimer featuring a previously unreported linkage of C-13–C-13′.

In parallel, four new sesquiterpenoid monomers, compounds **6**–**9** (versolanolides A–D), were isolated from *V*. *solanifolia* as putative monomeric constituents of dimers **1**–**5**.

**Fig. 4:**
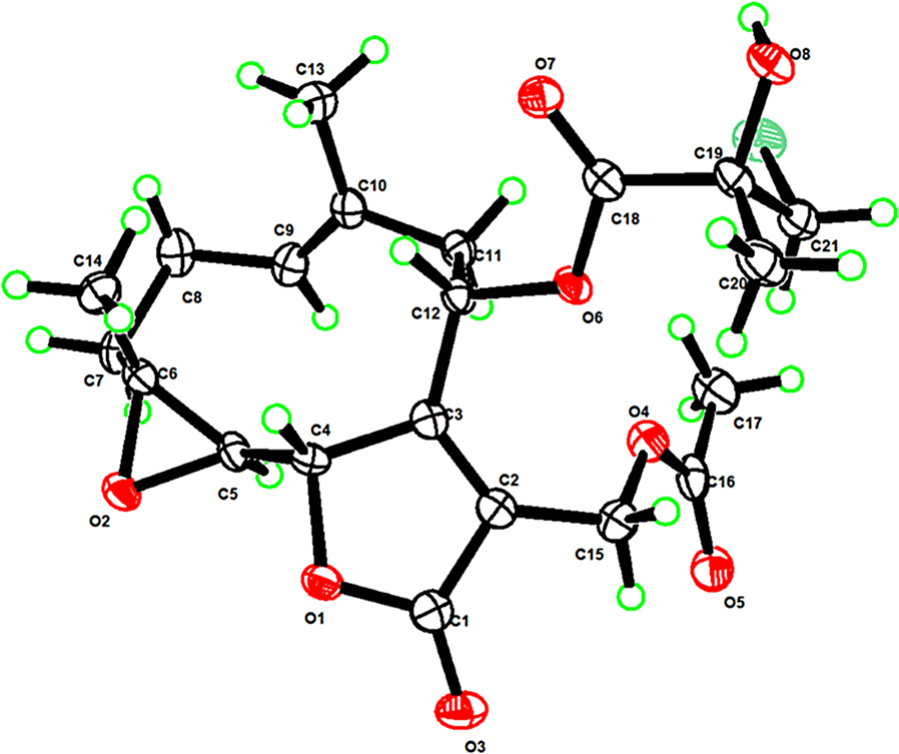
Perspective ORTEP drawing of compound **6**

Versolanolide A (**6**) was obtained as colorless crystals. The HRESIMS spectrum showed protonated molecular ion peaks at *m*/*z* 443.1465 and 445.1452 in a ratio of 3:1, indicative of one chlorine atom, establishing the molecular formula as C_21_H_27_ClO_8_. The IR spectrum of **6** indicated the presence of hydroxy (3464 cm^−1^) and carbonyl groups (1747 cm^−1^). The ^1^H NMR spectrum of **6** displayed one olefinic proton (*δ*_H_ 5.42, t, *J* = 8.1 Hz), two oxygenated methines (*δ*_H_ 5.20, dd, *J* = 10.4, 3.4 Hz; 4.96, dd, *J* = 8.9, 1.1 Hz), one oxygenated methylene (*δ*_H_ 5.08, d, *J* = 13.0 Hz; 4.87, d, *J* = 13.0 Hz), one chlorinated methylene (*δ*_H_ 3.75, d, *J* = 11.1 Hz; 3.69, d, *J* = 11.1 Hz), and four singlet methyl groups (*δ*_H_ 2.07, 1.74, 1.40, 1.38). A total of 21 carbon resonances were well resolved in the ^13^C NMR and DEPT-135 spectra, ascribed to four methyls, five methylenes, four methines, and eight quaternary carbons (Table 2). Comparison of the 1D and 2D NMR data revealed high similarity between compound **6** and 8-deacyl marginatin-methacrylate [33], differing only in the substituent at C-8. HMBC correlations from H_3_-8d (*δ*_H_ 1.40) to C-8a (*δ*_C_ 171.4), C-8b (*δ*_C_ 73.8), and C-8c (*δ*_C_ 50.2) indicated the presence of a 3-chloro-2-hydroxyl-2-methylpropanoyloxy moiety. NOESY correlations of H-8/H-6, H-6/H_3_-15, H_3_-15/H_2_-3*β*, and H-5/H_2_-3*α* designated a plausible relative configuration of **6** as shown in Fig. S17. The absolute configuration was finally established by single-crystal X-ray crystallographic diffraction with Cu K*α* radiation (CCDC 2164554, Fig. 4).

Compound **7** possessed a molecular formula of C_21_H_29_ClO_8_ as defined by HRESIMS and ^13^C NMR data (Table 2). The NMR data of **7** were highly similar to those of vernocinolide A [34], indicating that the two compounds were structural analogues. The major difference was the presence of a 1,1-disbustituented alkene moiety in **7** (*δ*_H_ 5.05, s, 5.00, s; *δ*_C_ 143.5, 114.0) in place of the methyl group and oxygenated quaternary carbon observed in vernocinolide A. The ^1^H‒^1^H COSY correlations of H-1/H-5 and H-8/H-9, together with the HMBC correlations from H-5 (*δ*_H_ 1.49), H-8 (*δ*_H_ 6.12) and H_2_-14 (*δ*_H_ 5.05, 5.00) to C-10 (*δ*_C_ 143.5), and from H-1 (*δ*_H_ 3.02) and H-9 (*δ*_H_ 2.84) to C-14 (*δ*_C_ 114.0), constructed an exocyclic double bond between C-10 and C-14 (Fig. S18).

In the NOESY spectrum, cross-peaks of 4-OH/H-1 and H-1/6-OH placed 4-OH, H-1, and 6-OH on the same face. The NOESY correlation of H-5/H-8 was also observed, implying spatial proximity. However, the correlation of the two groups of protons were uncertain due to the absence of a key NOESY correlation of H-1/H-5. Therefore, to designate unambiguously the relative configurations of **7**, two possible structures, *rel*-(1*R*,4*R*,5*R*,6*S*,8*R*)-**7a** and *rel*-(1*R*,4*R*,5*S*,6*S*,8*S*)-**7b** (Fig. S19), were generated for DFT NMR calculations. For computational efficiency, the ethoxy group at C-13 and the chloromethyl group at C-8b were replaced by hydroxy and methyl groups, respectively. Conformational search was carried out on these two simplified structures, and all conformers with Boltzmann population above 1.0% were selected for re-optimization at the level of B3LYP/6-31G(d) in vacuo. The unduplicated conformers were further used for the DFT calculation of ^1^H and ^13^C NMR chemical shifts at the level of mPW1PW91/6-311G(d,p) with the PCM model for chloroform. The theoretical chemical shifts of **7a** and **7b** were yielded after Boltzmann averaging over individual conformers based on their relative free energies. The improved statistical method DP4 + was used to analyze statistically the calculated data and the experimental ones [25]. The result gave 100% possibilities (H data, C data, and all data, Fig. S20) for **7b**. To further determine its absolute configuration, TDDFT ECD calculation was employed on the *rel*-(1*R*,4*R*,5*S*,6*S*,8*S*)-**7b** using the method of M06-2X/def2TZVP//M06-2X/6-311G(d,p) with the SMD solvent model of methanol. The calculated ECD spectrum gave a positive Cotton effect around 255 nm and a negative one around 200 nm, in good agreement with the experimental curve (Fig. S21). Thus, the absolute configuration of **7** was defined as 1*R*,4*R*,5*S*,6*S*,8*S*. Accordingly, the full structure of compound **7** was established, and named versolanolide B.

The HRESIMS of compound **8** gave a molecular formula of C_21_H_29_ClO_8_, consistent with seven indices of hydrogen deficiency. The ^1^H and ^13^C NMR spectra of **8** (Table 2) almost resembled those of the known compound vernocinolide A [34]. The molecular weight of **8** was 18 Da lower than that of vernocinolide A, suggesting the formation of an epoxide ring in **8** via dehydration. Compared with vernocinolide A, the chemical shifts of **8** at C-1, C-5, C-6, C-7, and C-10 shifted from *δ*_C_ 51.2, 55.3, 108.2, 158.1 and 72.4 to *δ*_C_ 46.2, 59.1, 103.7, 162.1 and 78.9. HMBC correlations from H_3_-14 (*δ*_H_ 1.30) to C-1 (*δ*_C_ 46.2), C-9 (*δ*_C_ 44.9), and C-10 (*δ*_C_ 78.9), from H-5 (*δ*_H_ 2.37) to C-6 (*δ*_C_ 103.7), C-7 (*δ*_C_ 162.1) and C-10 (*δ*_C_ 78.9), and from H-8 (*δ*_H_ 5.83) to C-6 (*δ*_C_ 103.7) established an epoxide bond between C-6 and C-10 (Fig. S18). In the NOESY spectrum, the cross-peaks of H_3_-15/H-5, H-5/H-2*β*, and H-5/H-3*β* suggested that these protons were co-facial and *β*-orientated. NOESY correlations of H-1/H-8, and H-1/H_3_-14 supported that these protons were spatially close. The small coupling constant of H-1 (*δ*_H_ 2.60, d, *J* = 3.1 Hz) indicated a *cis* relationship, confirming the *β*-orientation of H-5. The absolute configuration of **8** was determined by TDDFT ECD calculation on the truncated model of **8a** (Fig. S19). The calculated ECD spectrum of **8a** agreed well with the experimental one of **8** (Fig. S16). Therefore, the structure of **8** was fully established as versolanolide C with the absolute configuration of 1*R*,4*R*,5*S*,6*S*,8*S*,10*S*.

The HRESIMS data of **9** designated a molecular formula of C_21_H_29_ClO_8_, identical to that of **8**. Comparison of their NMR data (Table 2) revealed the presence of a ketone carbonyl at *δ*_C_ 208.8 in **9**, replacing the oxygenated quaternary carbon (C-4) in **8**. The spin system of H_2_-3/H_2_-2/H-1/H_2_-5 in the ^1^H‒^1^H COSY spectrum, together with the HMBC correlations from H_3_-15 (*δ*_H_ 2.19) to C-4 (*δ*_C_ 208.8), from H_2_-3 (*δ*_H_ 2.59, 2.46) to C-4 (*δ*_C_ 208.8), and from H_2_-5 (*δ*_H_ 2.49, 2.11) to C-2 (*δ*_C_ 23.6), C-7 (*δ*_C_ 155.2) and C-10 (*δ*_C_ 83.7), indicated that **9** was a *seco*-ring A derivative of **8**, belonging to the xanthane-type sesquiterpenes (Fig. S18). Detailed analysis of 2D NMR spectra further constructed the planar structure of **9**. The NOE cross-peaks of H-1/H_3_-14 indicated spatial proximity, consistent with the oxygen bridge between C-10 and C-6. In addition, the relative configuration of C-8 could not be determined by NOE correlations. Therefore, DFT NMR calculations were performed on four simplified structures **9a**‒**9d** (Fig. S22) using the same protocol as for **7**. DP4 + statistical analysis of the theoretical and experimental data supported the specific structure of **9c** (Fig. S23). The calculated ECD spectrum of **9c** matched the experimental one (Fig. S24). Accordingly, the structure of **9** was proposed, with the absolute configuration (1*R*,6*S*,8*R*,10*R*), and named versolanolide D. Notably, the C-8 configuration of compound **9** is opposite to that of** 8**, as reflected in the upfield shifts of C-7, C-8, and C-9 in **9** when compared to **8** (Table 2).

**Fig. 5:**
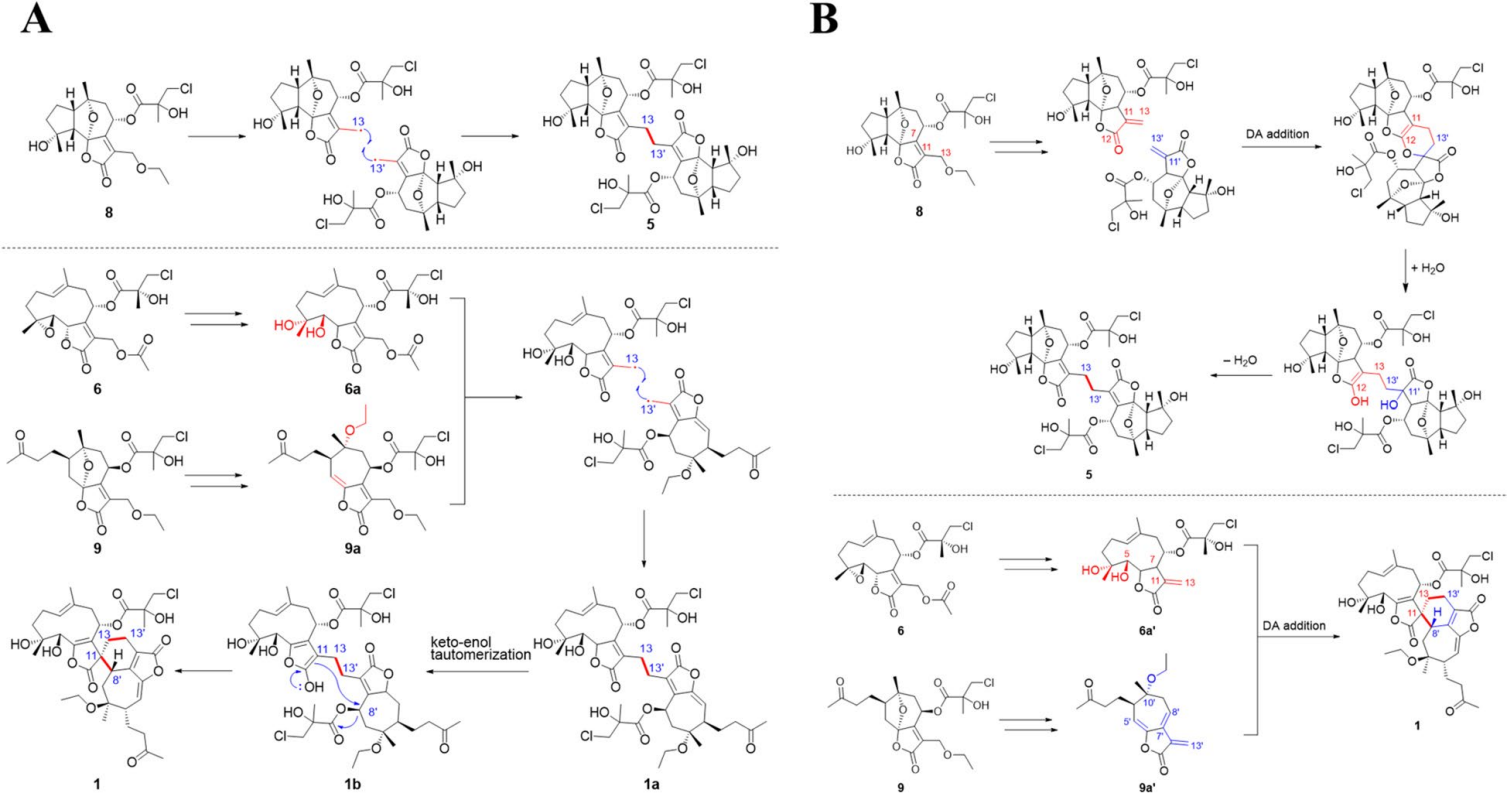
Possible biosynthetic pathways for compounds **1** and **5** via radical reactions (**A**) or Diels–Alder reactions (**B**)

Taken together, all heterodimers (**1**–**4**) and the homodimer (**5**) were composed of germacrene-, guaiane-, and *seco*-guaiane-type monomeric units, and their corresponding monomers (**6**–**9**) were isolated from the same plant. The co-occurrence of these monomeric and dimeric compounds implied that the plant might employ the monomeric block to assemble both the homo- and heterodimers. Plausible biogenetic pathways for compounds **1** and **5** were proposed involve either radical-mediated coupling (Fig. 5A) or Diels–Alder cycloadditions (Fig. 5B). These two dimerization strategies represent established mechanisms in the biosynthetic assembly of sesquiterpenoid dimers [35].

It was noted that all the monomers isolated from *V. solanifolia* possessed acetoxy or ethoxy substitutions at C-13. Given diversolanolide E (**5**) is a homodimer linked via C-13/C-13', it is proposed that the C-13/C-13' bond could be formed through a free radical coupling following loss of the ethoxy group from versolanolide C (**8**). Subsequently, versolanolides A (**6**) and D (**9**) could serve as biosynthetic precursors for diversolanolide A (**1**). A series of ring-opening and oxidation reactions would produce intermediates **6a** and **9a**, followed by free radical reactions upon loss of the acetyl and ethoxy groups, affording key intermediate **1a**. The intermediate **1b** would subsequently be formed by the keto-enol tautomerism of **1a**. Finally, **1** would be formed via intramolecular C-11-C-8' bond formation.

Alternatively, the formations of compounds **5** and **1** could be achieved through Diels–Alder cycloaddition reactions. Compound **8**, with the removal of the ethoxy group at the C-13 position, forms a C-11/C-13 double bond. This *α*,*β*-unsaturated ketone segment serves as both a diene and a dienophile, leading to the formation of a crucial intermediate via a hetero-Diels–Alder cycloaddition reaction. Subsequent hydration and ring-opening, followed by dehydration, result in the synthesis of compound **5**. Similarly, compounds **6** and **9**, the possible precursors of compound **1**, were speculated to undergo a transformation to form key intermediates, **6a'** and **9a'**, both featuring a C-11/C-13 double bond akin to that in compound **8**. In these intermediates, the C-11/C-13 double bond in **6a'** was proposed to act as the dienophile, while the 1,3-butadiene segment (C-8'-C-7'-C-11'-C-13') in **9a'** served as the diene. A subsequent Diels–Alder cycloaddition reaction between these intermediates eventually led to the formation of compound **1**.

In a similar manner, compounds **2**–**4** could presumably arise from the corresponding monomeric precursors (**6**–**9**). Ethoxy substituents were observed at multiple positions across the isolated compounds: C-5 in **2**, C-10′ in **1**, **2**, and **4**, and C-13 in **7**–**9**. Given that 95% ethanol was used as the extraction solvent, the occurrence of ethoxy groups in these structures is most likely attributed to acid-catalyzed nucleophilic addition, alcoholysis, or etherification reactions between electron-rich double bonds, epoxide moieties, or hydroxyl groups within the sesquiterpenoid skeletons and ethanol during the extraction process.

### Lipid-lowering activity

**Fig. 6:**
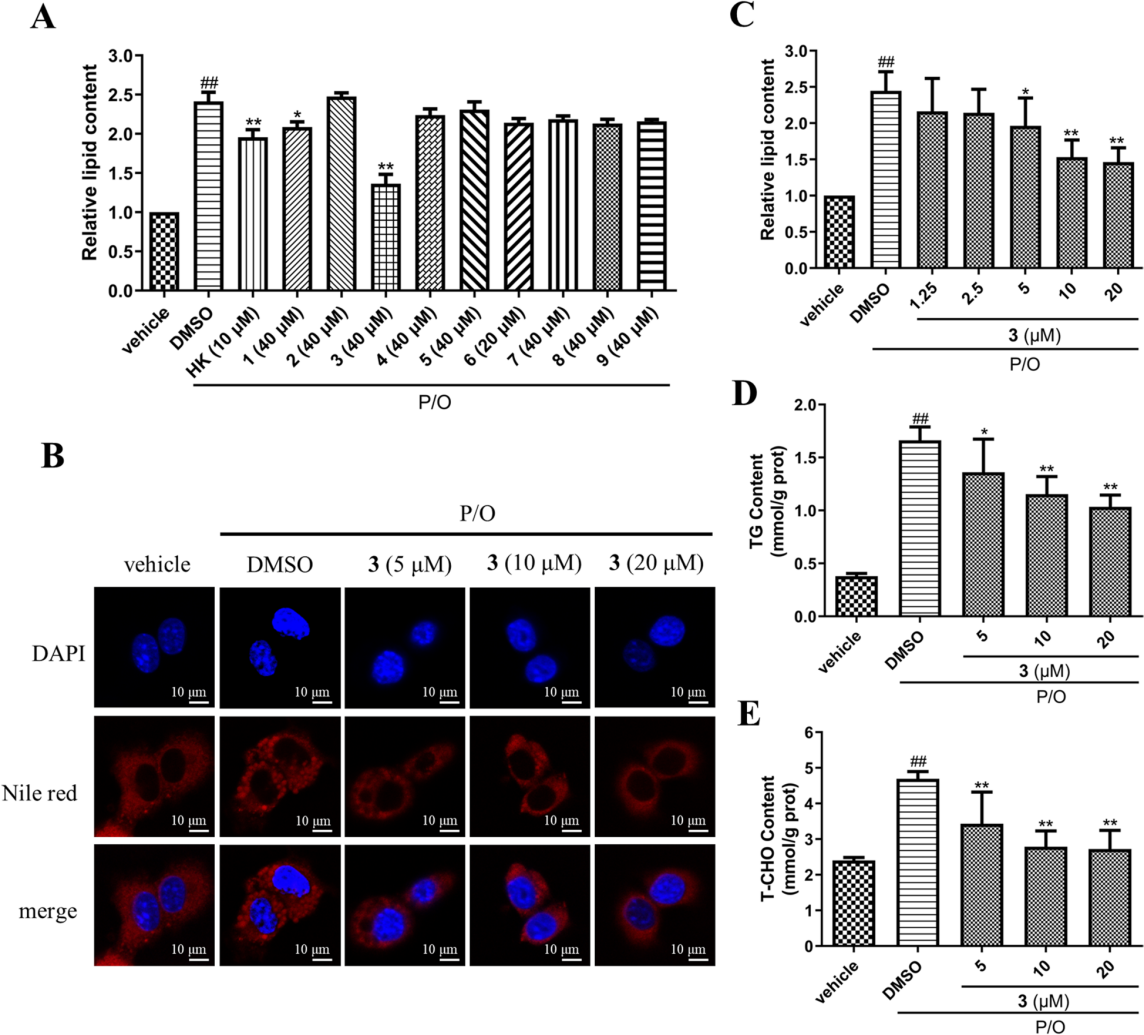
The lipid lowering effect of compound **3** in P/O-treated AML12 hepatocytes. **A** The lipid lowering effect of compounds **1**–**9** at their maximum safe concentrations in P/O-treated AML12 hepatocytes. **B** Intracellular lipid content was visualized with Nile red staining (red). Nuclei were stained with DAPI (blue). Scale bar = 10 µm. **C** The quantitation of lipid content determined by Nile red staining. **D** The cellular TG content was determined. **E** The cellular T-CHO content was determined. Data are shown as mean ± S.D., n = 3. ^##^*P* < 0.01 vs. vehicle; *P < 0.05, **P < 0.01 vs. P/O

The plants from *Vernonia* genus have been proved to have lipotropic activities [36, 37], thus compounds **1**–**9** were evaluated for their lipid-lowing effect on palmitic acid/oleic acid (P/O)-treated AML12 hepatocytes. Firstly, the maximum safe concentrations of compounds **1**–**9** on AML12 hepatocytes were evaluated by the MTT assay. The MTT results showed that compound **9** did not show evident cytotoxicity up to 20 μM, and the maximum safe concentration of other compounds was 40 μM (Fig. S25). Nile red staining results indicated that the treatment of P/O mixture significantly increased lipid content in AML12 hepatocytes; pretreatment of compounds **1** and **3** at 40 μM obviously reduced lipid accumulation in P/O-treated cells, which was comparable with the positive control, honokiol (HK, 10 μM) (Fig. 6A). As the most potent one, compound **3** was chosen for further studies. The fluorescent images and quantitation of Nile red staining showed that compound **3** dose-dependently reduced P/O-induced elevation of lipid content in AML12 cells (Fig. 6B, C). In addition, compound **3** reduced the contents of TG and T-CHO in P/O-induced AML12 hepatocytes in dose-dependent manners (Fig. 6D, E). Taken together, compound **3** exhibited a pronounced lipid-lowering effect in P/O-induced AML12 hepatocytes.

**Fig. 7:**
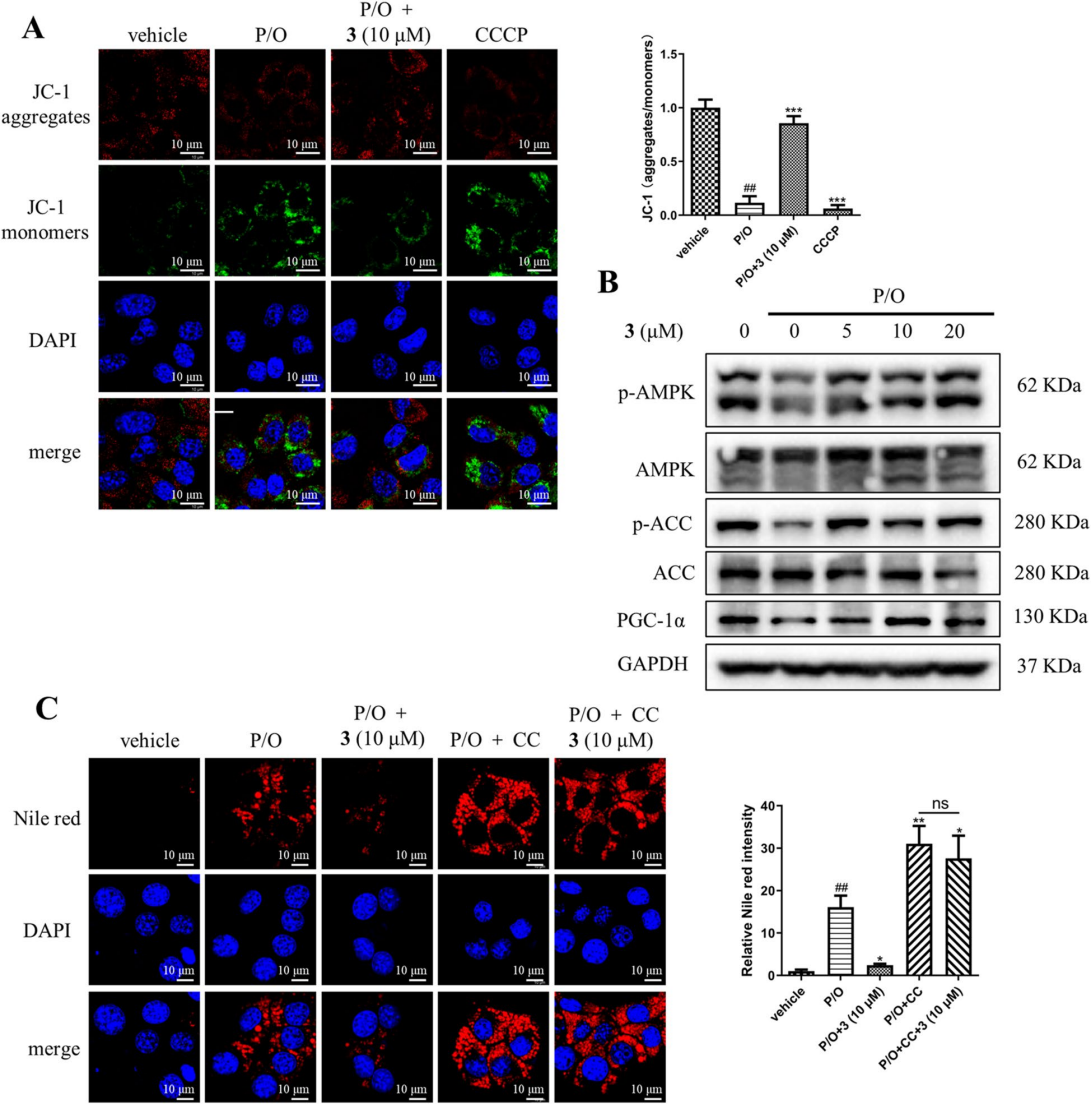
Compound **3** protected mitochondrial function in P/O-treated AML12 hepatocytes through activating AMPK/ACC/PGC-1*α* signaling pathway. **A** Compound **3** protected AML12 cells from P/O-induced depolarization of membrane potential. JC-1 monomers (green) and JC-1 aggregates (red) were visualized. CCCP was used as a depolarization-positive control. Scale bar = 10 µm. **B** Compound **3** activated AMPK/ACC/PGC-1*α* signaling pathway in P/O-treated AML12 hepatocytes. **C** Nile red staining in P/O-induced AML12 cells treated with compound **3**, in the presence or absence of CC. Data are shown as mean ± S.D., n = 3. ^##^*P* < 0.01 vs. vehicle; *P < 0.05, **P < 0.01, ***P < 0.001 vs. P/O

Mitochondria act as an important site of fatty acid *β*-oxidation, the maintenance of mitochondrial membrane potential is tightly connected to mitochondrial function [38]. P/O treatment significantly decreased the ratio of JC-1 aggregates (Red) to JC-1 monomers (Green), indicating disrupted mitochondrial membrane potential; and compound **3** (10 μM) treatment totally reversed the above change (Fig. 7A). CCCP (10 µM) was used as a depolarization-positive control (Fig. 7A). AMP-activated protein kinase (AMPK) acts as a crucial energy sensor that regulates energy supply and lipid metabolism [39]. Acetyl-CoA (ACC) is a rate-limiting enzyme in fatty acid synthesis. AMPK activation inhibits lipogenesis by inducing ACC phosphorylation to suppress the conversion of acetyl-CoA to malonyl-CoA [40]. Peroxisome proliferator-activated receptor γ co-activator 1*α* (PGC-1*α*) is the master transcriptional cofactor to regulate mitochondrial biogenesis and function [41]. AMPK activation enhances the transcriptional activity of PGC-1*α*, thereby promoting mitochondrial function [42]. As shown in Fig. 7B, P/O treatment decreased the phosphorylation levels of AMPK and ACC, and the protein expression level of PGC-1*α*; while compound **3** obviously reversed those changes. Furthermore, Compound C (CC) was used to verify whether compound **3** inhibits lipid accumulation via the AMPK signaling pathway. Nile Red results showed that co-treatment of CC largely abolished the effect of compound **3** on decreasing lipid accumulation (Fig. 7C). These observations indicated that the lipid lowering effect of compound **3** might be mediated through activating AMPK/ACC/PGC-1*α* signaling pathway.

## Conclusion

In summary, homo- and hetero-dimeric sesquiterpenoids (**1**–**5**) were fully characterized from *V. solanifolia*, together with their co-occurring monomers (**6–9**). This marks the first report of dimeric sesquiterpenoids from *V. solanifolia* [10]. Additionally, the unique linkage patterns observed between the constituent halves, namely the spiro cyclohexene ring and the ethylene bridge, have not been reported in known dimeric structures within the *Vernonia* genus. Furthermore, this study reports, for the first time, the lipid-lowering activity of natural compounds isolated from this genus, complementing the previously reported cytotoxic, antiplasmodial, anti-inflammatory, and other biological activities [10]. In conclusion, these findings not only expand the chemical diversity within the *Vernonia* genus but also lay a foundation for future pharmacological investigations and potential drug development.

## Supplementary Information

Supplementary material 1.

## Data Availability

Data will be made available on request.

## Abbreviations

ACC: Acetyl-CoA
AMPK: AMP-activated protein kinase
CC: Compound C
DAPI: 4′,6-Diamidino-2-phenylindole
DFT: Density-functional theory
DMEM: Dulbecco’s Modified Eagle Medium
DMSO: Dimethyl sulfoxide
ECD: Electronic circular dichroism
ELSD: Evaporative light scattering detector
FBS: Fetal bovine serum
HK: Honokiol
HPLC: High-pressure liquid chromatography
HRESIMS: High-resolution electrospray ionization mass spectrometry
IR: Infrared
MTT: [3-(4,5-Dimethylthiazol-2-yl)-2,5-diphenyltetrazolium bromide]
NMR: Nuclear magnetic resonance
PBS: Phosphate-buffered saline
PGC-1*α*: Peroxisome proliferator-activated receptor γ co-activator 1*α*
P/O: Palmitic acid/oleic acid
TC: Total cholesterol
TDDFT: Time-dependent density-functional theory
TG: Total glycerides
TLC: Thin-layer chromatography
UV: Ultraviolet
