# Supplementary material for: Homo- and hetero-dimeric sesquiterpenoids with unprecedented skeletons and their co-occurring monomers from Vernonia solanifolia with anti-liver steatotic potential
Source: Chin Med. 2026 May 28;21:150. doi: 10.1186/s13020-026-01418-9 (PMC13217702; doi:10.1186/s13020-026-01418-9)
Supplement: Supplementary file 1 — Supplementary material 1. [file 13020_2026_1418_MOESM1_ESM.docx]

**Homo- and hetero-dimeric sesquiterpenoids with unprecedented skeletons and their co-occurring monomers from *Vernonia solanifolia* with anti-liver steatotic potential**

Yue Yang^1†^, Fei Zhou^2†^, Chang-Qiang Ke^1^, Sheng Yao^1,3^, Ligen Lin^2^*, Chunping Tang^1^* and Yang Ye^1,4^*

^1^State Key Laboratory of Drug Research, and Natural Products Chemistry Department, Shanghai Institute of Materia Medica, Chinese Academy of Sciences, Shanghai 201203, China. ^2^State Key Laboratory of Mechanism and Quality of Chinese Medicine, Institute of Chinese Medical Sciences, University of Macau, Macau 999078, China. ^3^Zhongshan Institute for Drug Discovery, Shanghai Institute of Materia Medica, Chinese Academy of Sciences, Zhongshan 528400, China. ^4^China-Serbia “Belt and Road” Joint Laboratory for Natural Products and Drug Discovery, Shanghai Institute of Materia Medica, Chinese Academy of Sciences, Shanghai 201203, China.

^†^Yue Yang and Fei Zhou contributed equally to this work.

**Correspondence:**

Ligen Lin

ligenl@um.edu.mo

Chunping Tang

tangcp@simm.ac.cn

Yang Ye

yye@mail.shcnc.ac.cn

**Table of contents**

[**Fig. S1** ^1^H–^1^H COSY, and key HMBC correlations of compounds **2** and **4**–**5**. 3](#_Toc162905636)

[**Fig. S2** Key NOESY correlations of compounds **4** and **5**. 3](#_Toc162905637)

[**Fig. S3** Simplified structures of unit A of compounds **1**–**3** with their possible relative configurations. 3](#_Toc162905638)

[**Fig. S4** DP4+ probability statistical analysis result for the unit A of compound **1**. 4](#_Toc162905639)

[**Fig. S5** DP4+ probability statistical analysis result for the unit A of compound **2**. 4](#_Toc162905640)

[**Fig. S6** DP4+ probability statistical analysis result for the unit A of compound **3**. 4](#_Toc162905641)

[**Fig. S7** The structures of **B1**–**B4**. 4](#_Toc162905642)

[**Fig. S8** DP4+ probability statistical analysis result for the **B1**–**B4** of compound **1**. 5](#_Toc162905643)

[**Fig.S9** DP4+ probability statistical analysis result for the **B1**–**B4** of compound **2**. 5](#_Toc162905644)

[**Fig. S10** DP4+ probability statistical analysis result for the **B1**–**B4** of compound **4**. 5](#_Toc162905645)

[**Fig. S11** The structures of **C1**–**C4**. 5](#_Toc162905646)

[**Fig. S12** DP4+ probability statistical analysis result for the **C1**–**C4** of compound **3**. 6](#_Toc162905647)

[**Fig. S13** Simplified structures of compounds **1** and **2** with their possible relative configurations. 6](#_Toc162905648)

[**Fig. S14** Simplified structures of compound **3** with their possible relative configurations. 6](#_Toc162905649)

[**Fig. S15** Simplified structures of compound **4** with their possible relative configurations. 7](#_Toc162905650)

[**Fig. S16** Experimental ECD spectra of compounds **5** and **8**, and calculated ECD spectrum of **8**. 7](#_Toc162905651)

[**Fig. S17** key 2D NMR correlations of compound **6**. 7](#_Toc162905652)

[**Fig. S18** key 2D NMR correlations of compounds **7**–**9**. 8](#_Toc162905653)

[**Fig. S19** Simplified structures of compounds **7** and **8** with their possible relative configurations. 8](#_Toc162905654)

[**Fig. S20** DP4+ probability statistical analysis result for compound **7**. 9](#_Toc162905655)

[**Fig. S21** Experimental and calculated ECD spectra of compound **7**. 9](#_Toc162905656)

[**Fig. S22** Simplified structures of compound **9** with their possible relative configurations. 9](#_Toc162905657)

[**Fig. S23** DP4+ probability statistical analysis result for compound **9**. 10](#_Toc162905658)

[**Fig. S24** Experimental and calculated ECD spectra of compound **9**. 10](#_Toc162905659)

[**Fig. S25** Cytotoxic effect of compounds **1**‒**9** on AML-12 hepatocytes. AML-12 cells were treated with different compounds at indicated concentrations for 24 h. Data are shown as mean ± S.D., n = 3. ***P* < 0.01 vs. DMSO. 11](#_Toc162905660)

[**Fig. S26**–**S31** The original Western blots. 12](#_Toc162905661)

[**Fig. S32**−**S40** MS, UV, IR, 1D and 2D NMR spectra of diversolanolide A (**1**). 15](#_Toc162905662)

[**Fig. S41**−**S49** MS, UV, IR, 1D and 2D NMR spectra of diversolanolide B (**2**). 19](#_Toc162905663)

[**Fig. S50**−**S58** MS, UV, IR, 1D and 2D NMR spectra of diversolanolide C (**3**). 24](#_Toc162905664)

[**Fig. S59**−**S67** MS, UV, IR, 1D and 2D NMR spectra of diversolanolide D (**4**). 28](#_Toc162905665)

[**Fig. S68**−**S76** MS, UV, IR, 1D and 2D NMR spectra of diversolanolide E (**5**). 33](#_Toc162905666)

[**Fig. S77**−**S85** MS, UV, IR, 1D and 2D NMR spectra of versolanolide A (**6**). 37](#_Toc162905667)

[**Fig. S86**−**S94** MS, UV, IR, 1D and 2D NMR spectra of versolanolide B (**7**). 42](#_Toc162905668)

[**Fig. S95**−**S103** MS, UV, IR, 1D and 2D NMR spectra of versolanolide C (**8**). 46](#_Toc162905669)

[**Fig. S104**−**S112** MS, UV, IR, 1D and 2D NMR spectra of versolanolide D (**9**). 51](#_Toc162905670)

## **Fig. S1** ^1^H–^1^H COSY, and key HMBC correlations of compounds **2** and **4**–**5**.

## **Fig. S2** Key NOESY correlations of compounds **4** and **5**.

## **Fig. S3** Simplified structures of unit A of compounds **1**–**3** with their possible relative configurations.

##
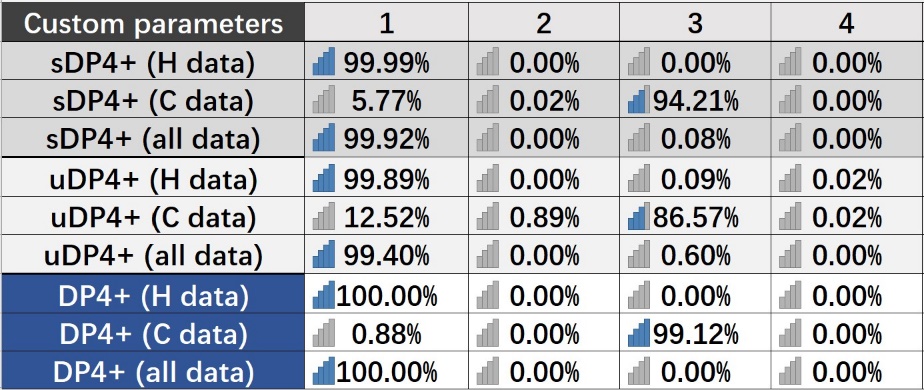

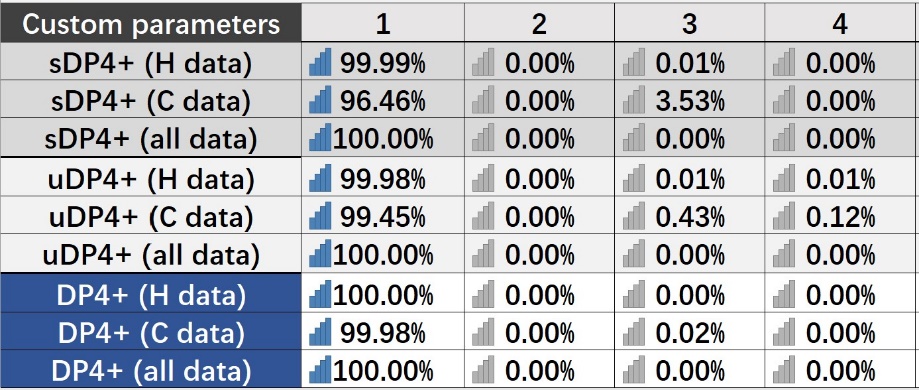
**Fig. S4** DP4+ probability statistical analysis result for the unit A of compound **1**.

## **Fig. S5** DP4+ probability statistical analysis result for the unit A of compound **2**.

##
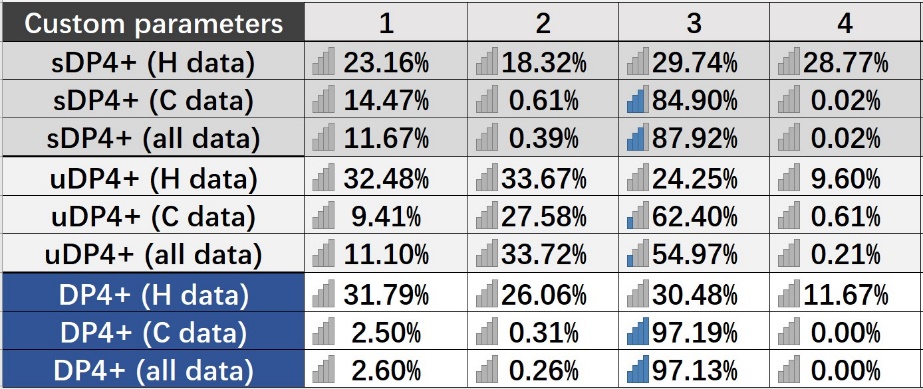
**Fig. S6** DP4+ probability statistical analysis result for the unit A of compound **3**.

## **Fig. S7** The structures of **B1**–**B4**.

##
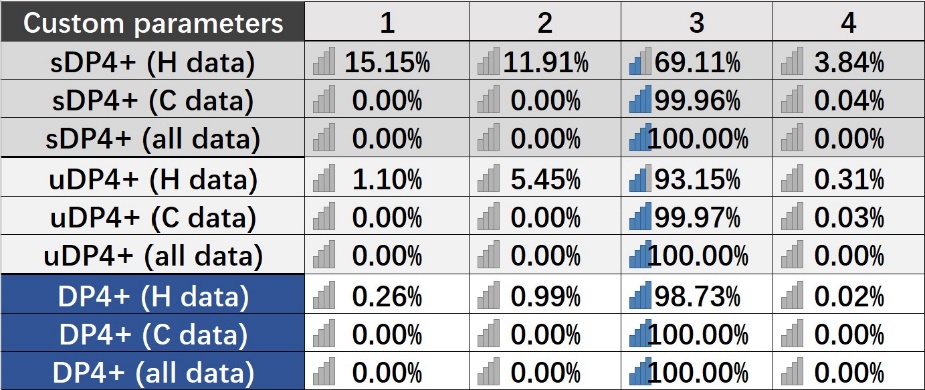

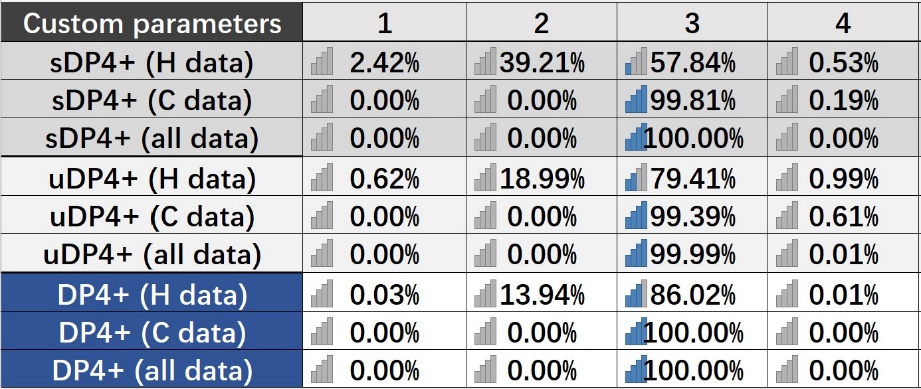
**Fig. S8** DP4+ probability statistical analysis result for the **B1**–**B4** of compound **1**.

## **Fig. S9** DP4+ probability statistical analysis result for the **B1**–**B4** of compound **2**.

##
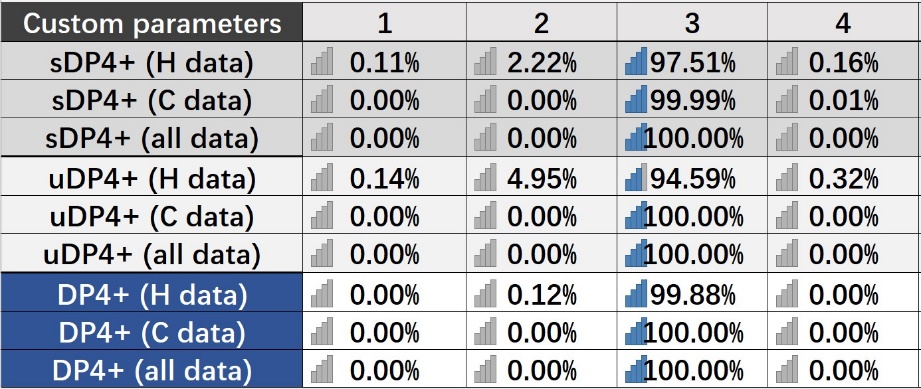
**Fig. S10** DP4+ probability statistical analysis result for the **B1**–**B4** of compound **4**.

## **Fig. S11** The structures of **C1**–**C4**.

##
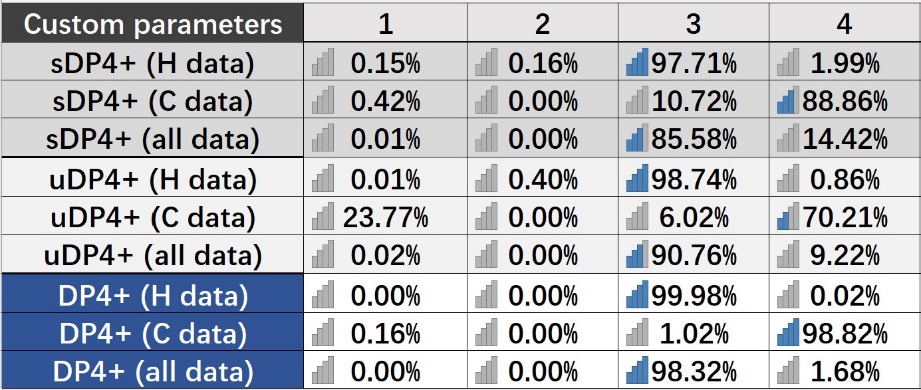
**Fig. S12** DP4+ probability statistical analysis result for the **C1**–**C4** of compound **3**.

## **Fig. S13** Simplified structures of compounds **1** and **2** with their possible relative configurations.

## **Fig. S14** Simplified structures of compound **3** with their possible relative configurations.

## **Fig. S15** Simplified structures of compound **4** with their possible relative configurations.





## **Fig. S16** Experimental ECD spectra of compounds **5** and **8**, and calculated ECD spectrum of **8**.

## **Fig. S17** key 2D NMR correlations of compound **6**.

## **Fig. S18** key 2D NMR correlations of compounds **7**–**9**.

## **Fig. S19** Simplified structures of compounds **7** and **8** with their possible relative configurations.


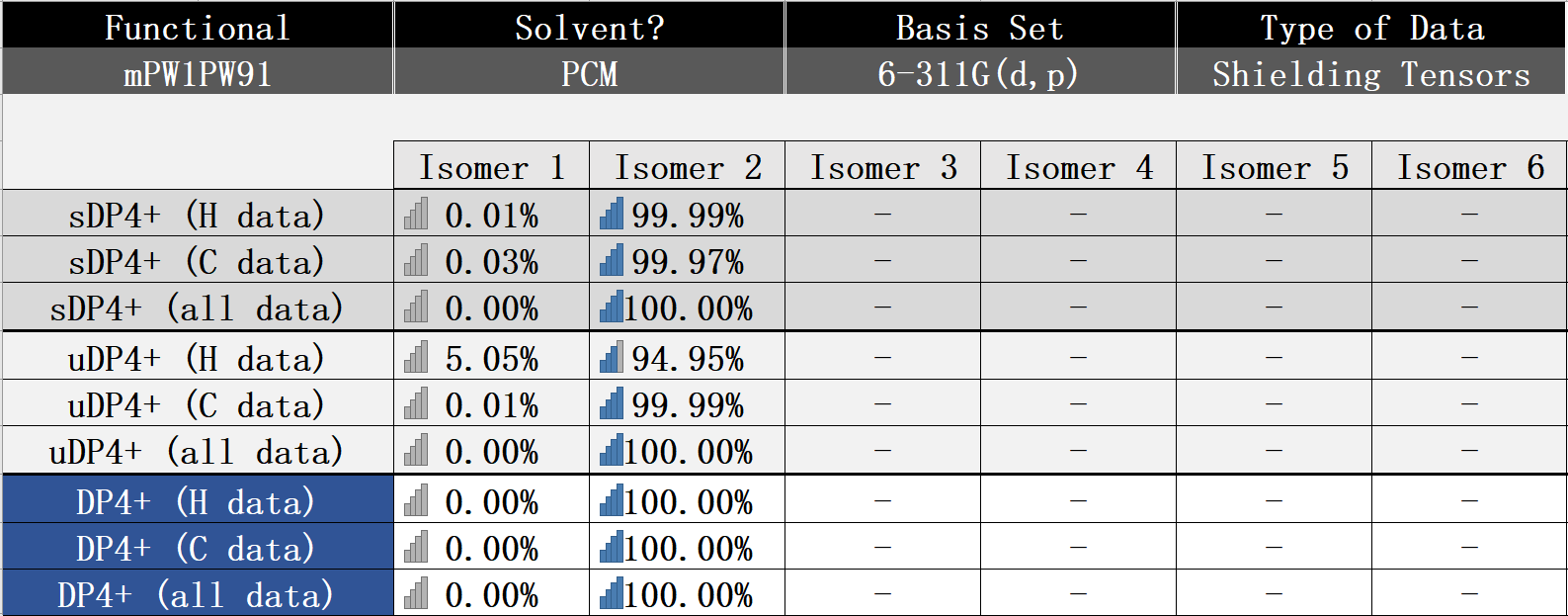


## **Fig. S20** DP4+ probability statistical analysis result for compound **7**.





## **Fig. S21** Experimental and calculated ECD spectra of compound **7**.

## **Fig. S22** Simplified structures of compound **9** with their possible relative configurations.


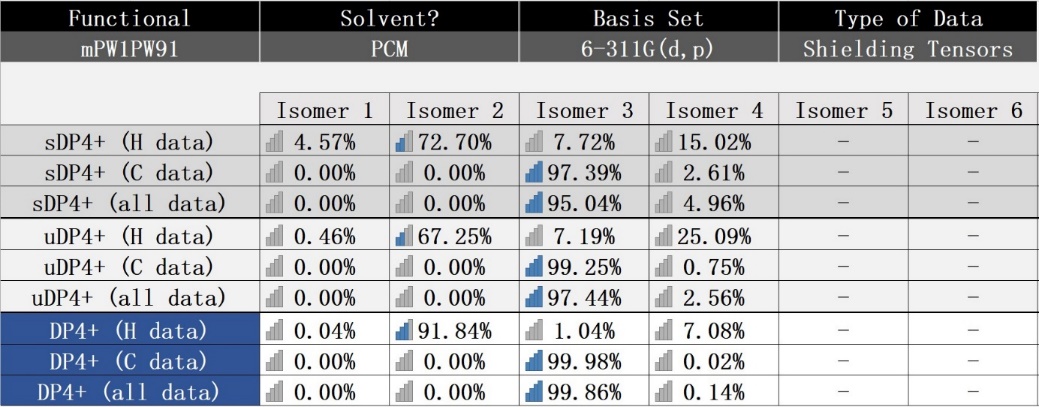


## **Fig. S23** DP4+ probability statistical analysis result for compound **9**.





## **Fig. S24** Experimental and calculated ECD spectra of compound **9**.


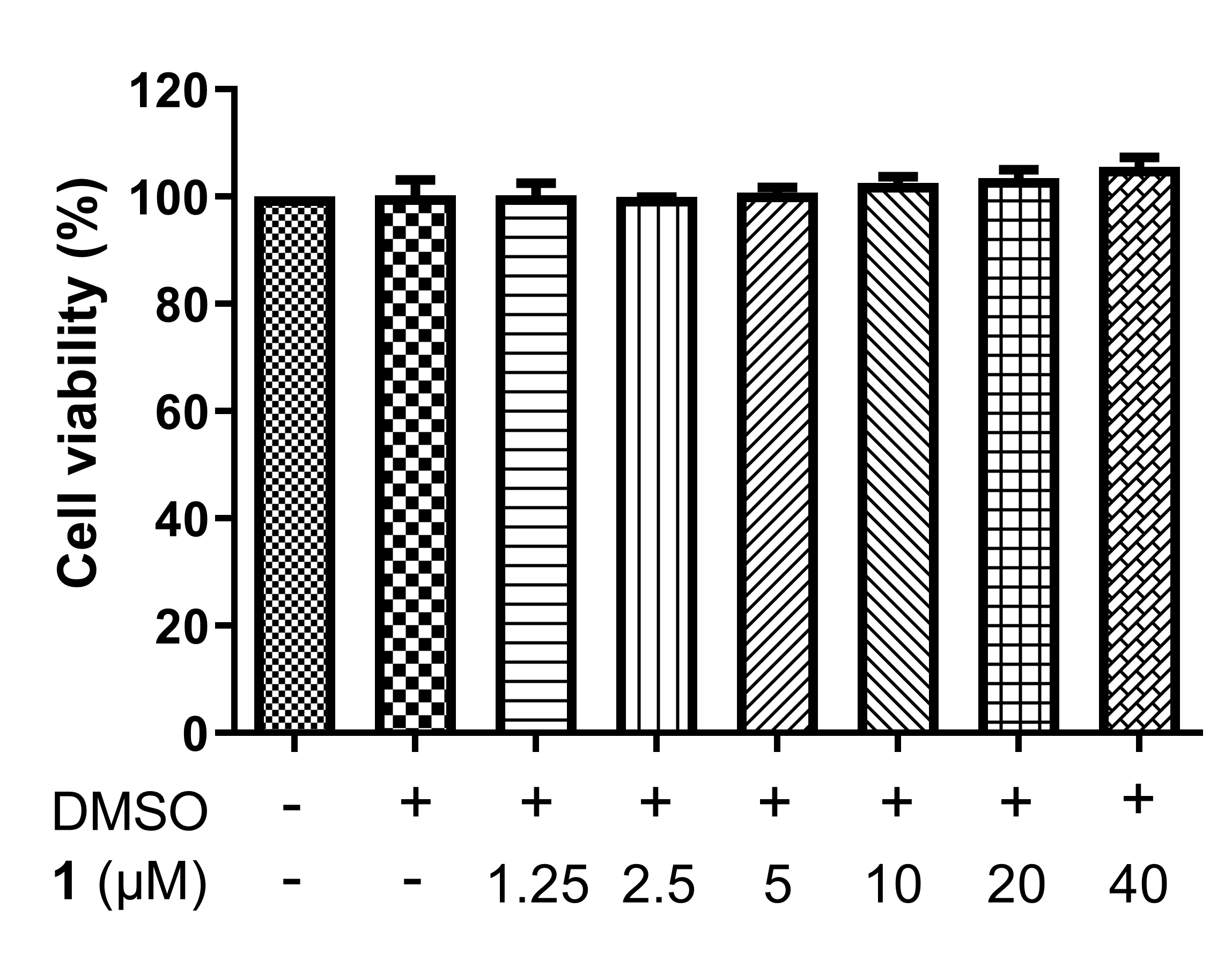

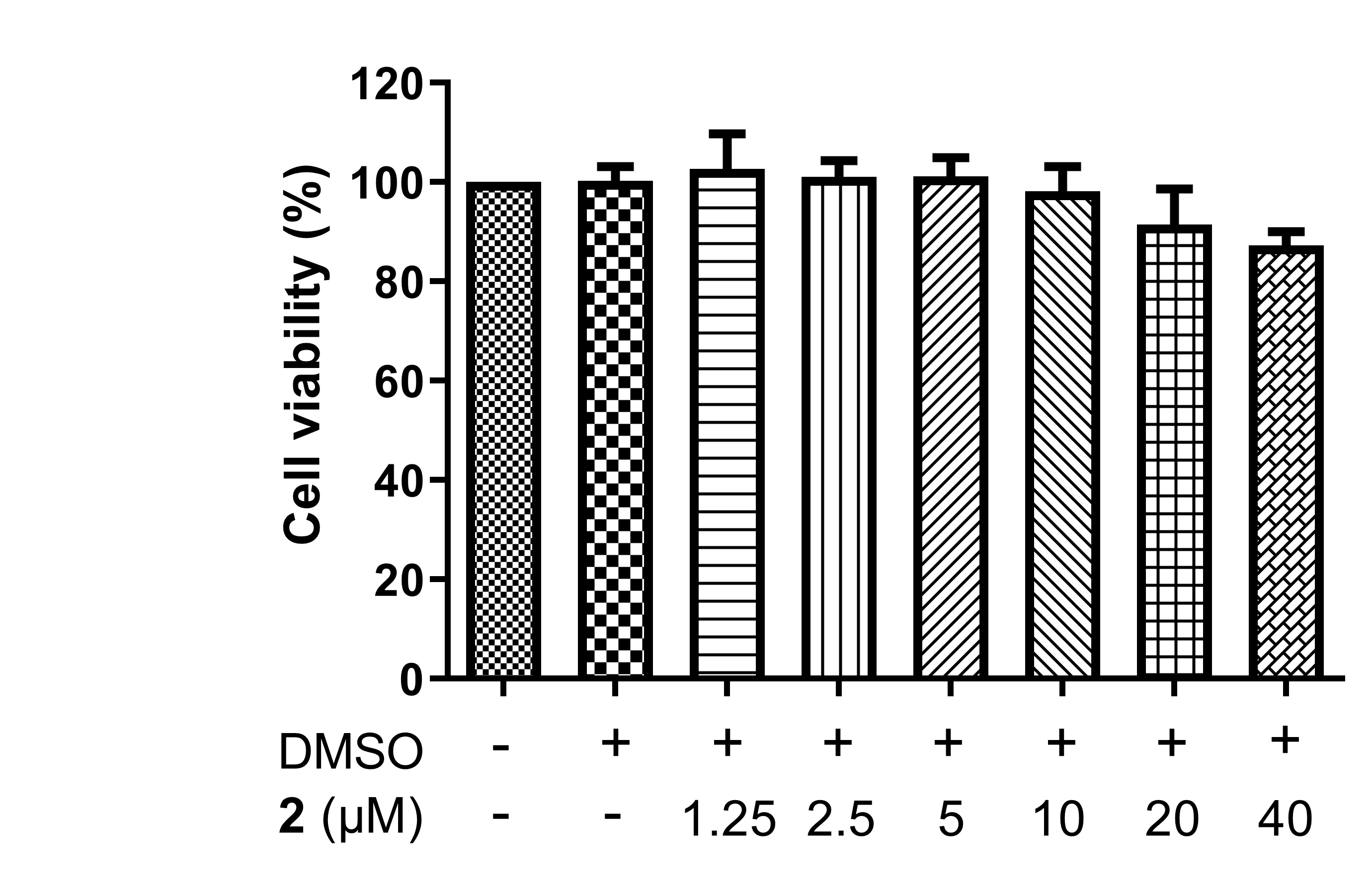

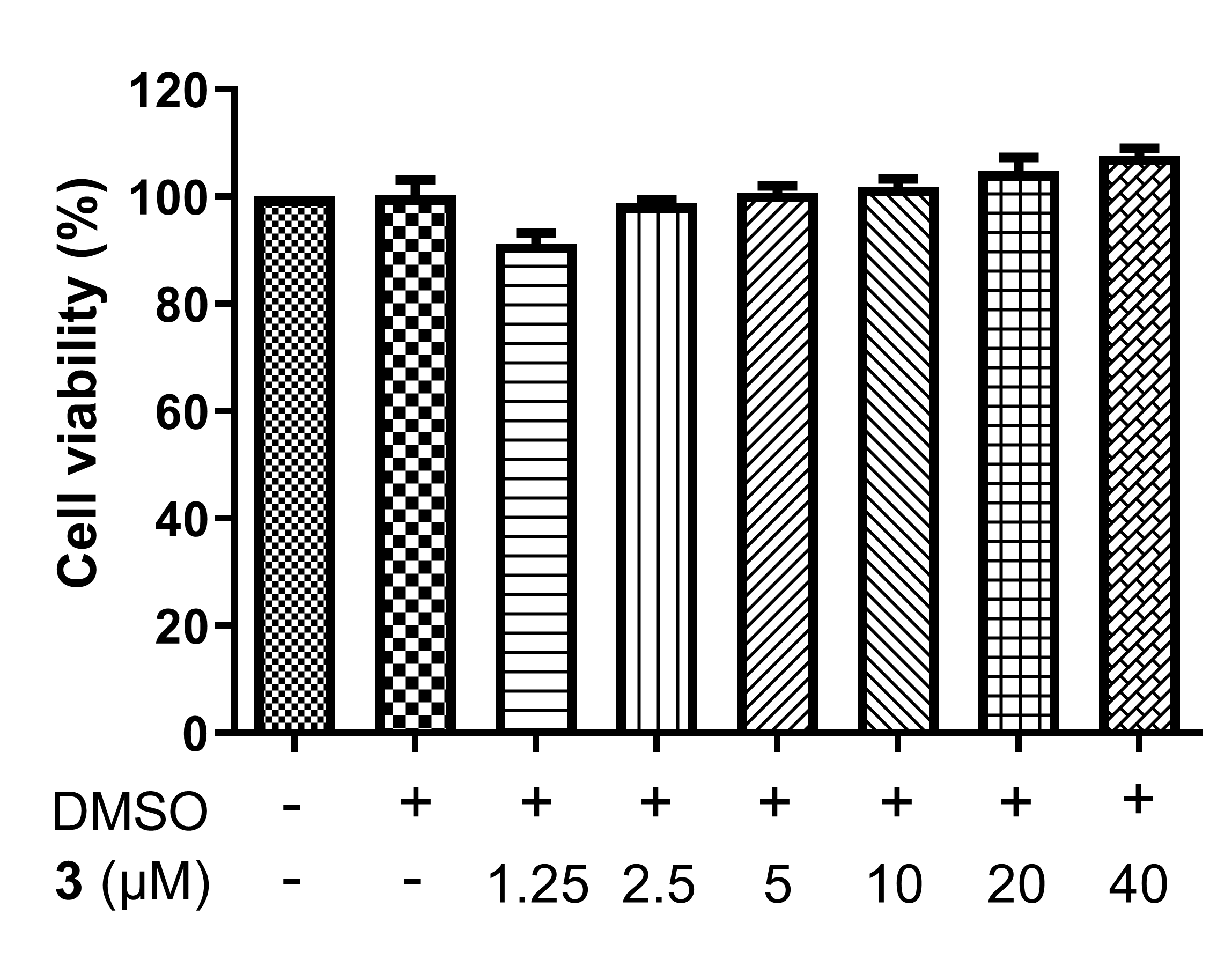

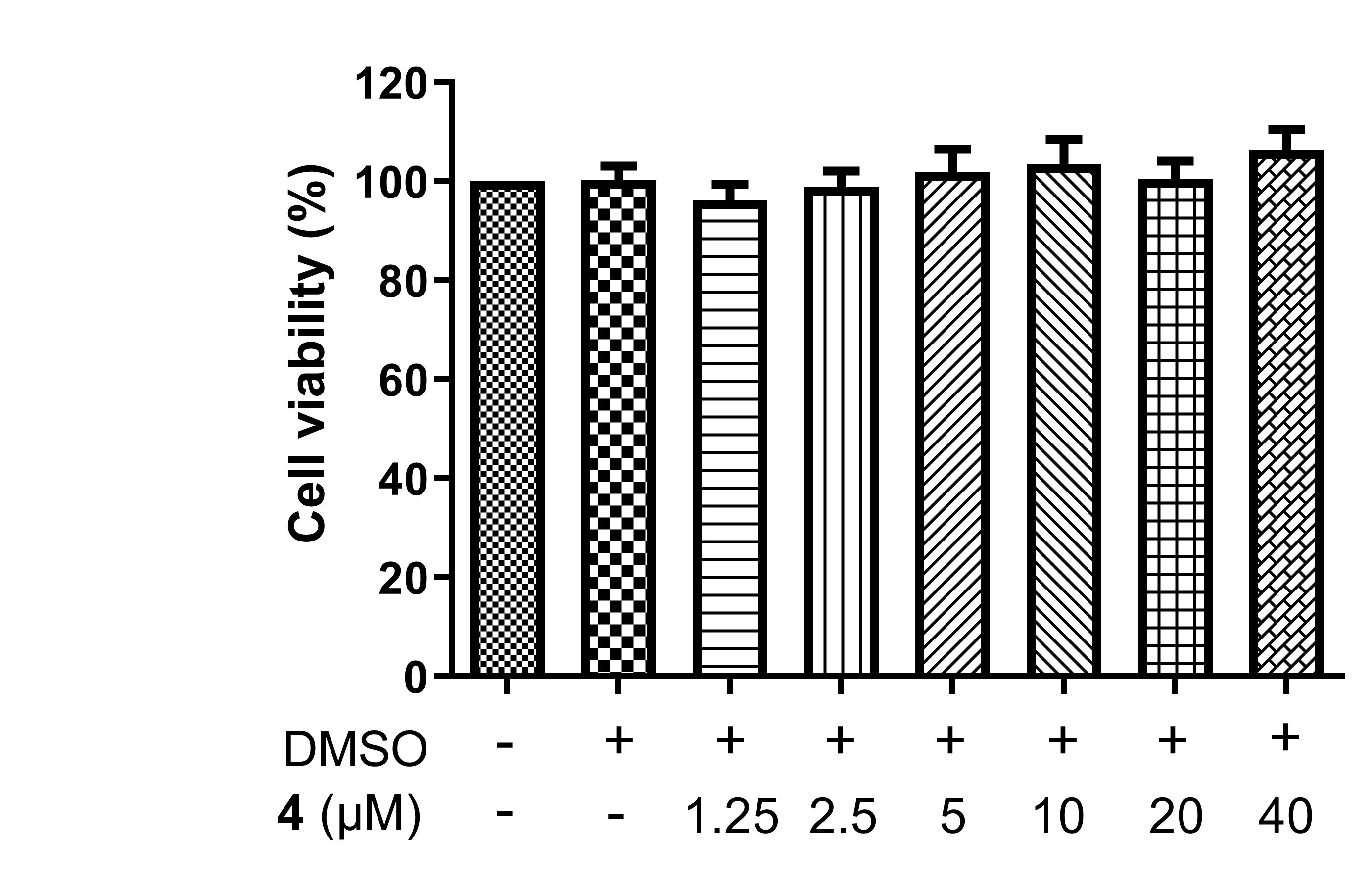

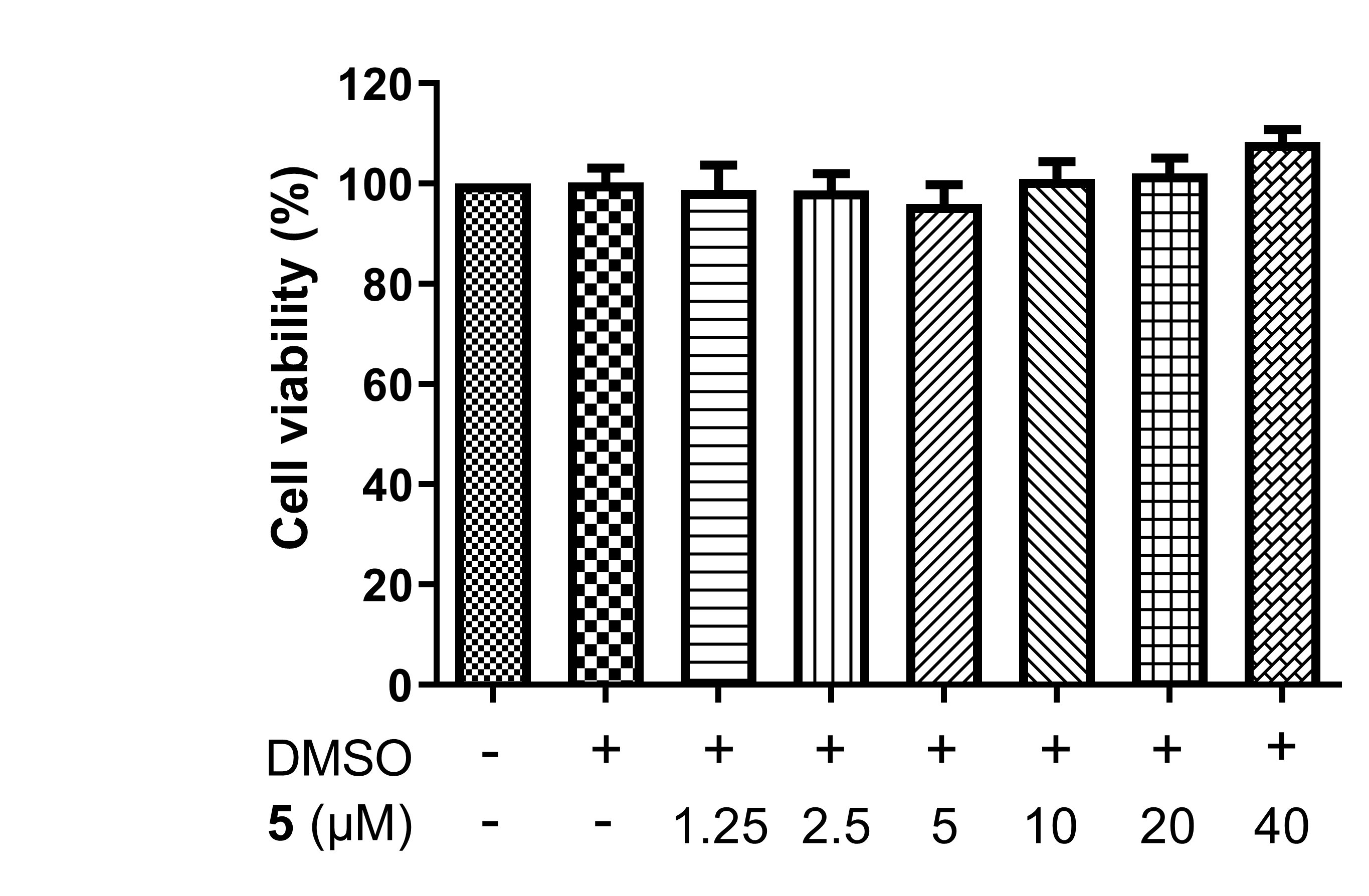

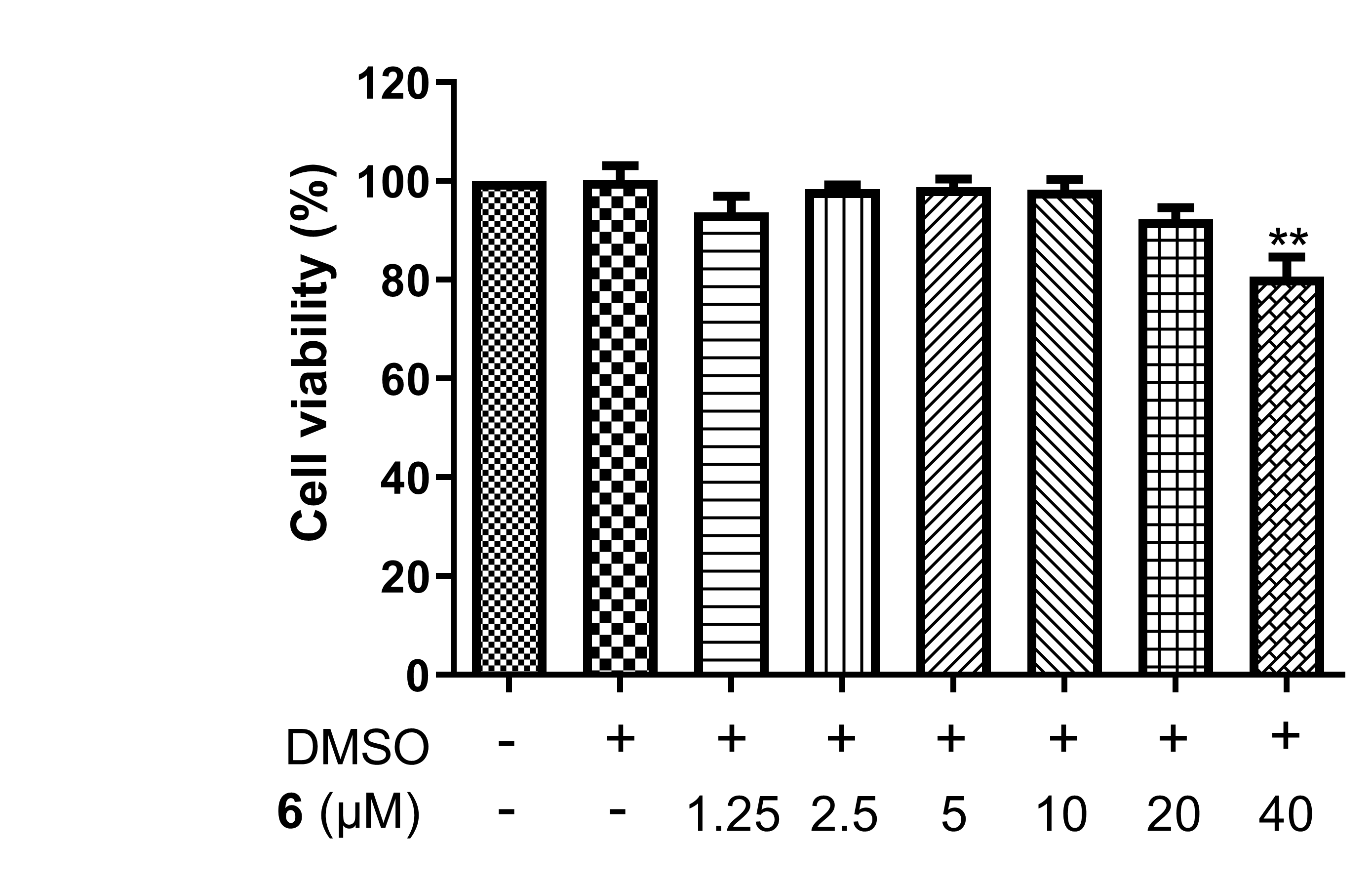

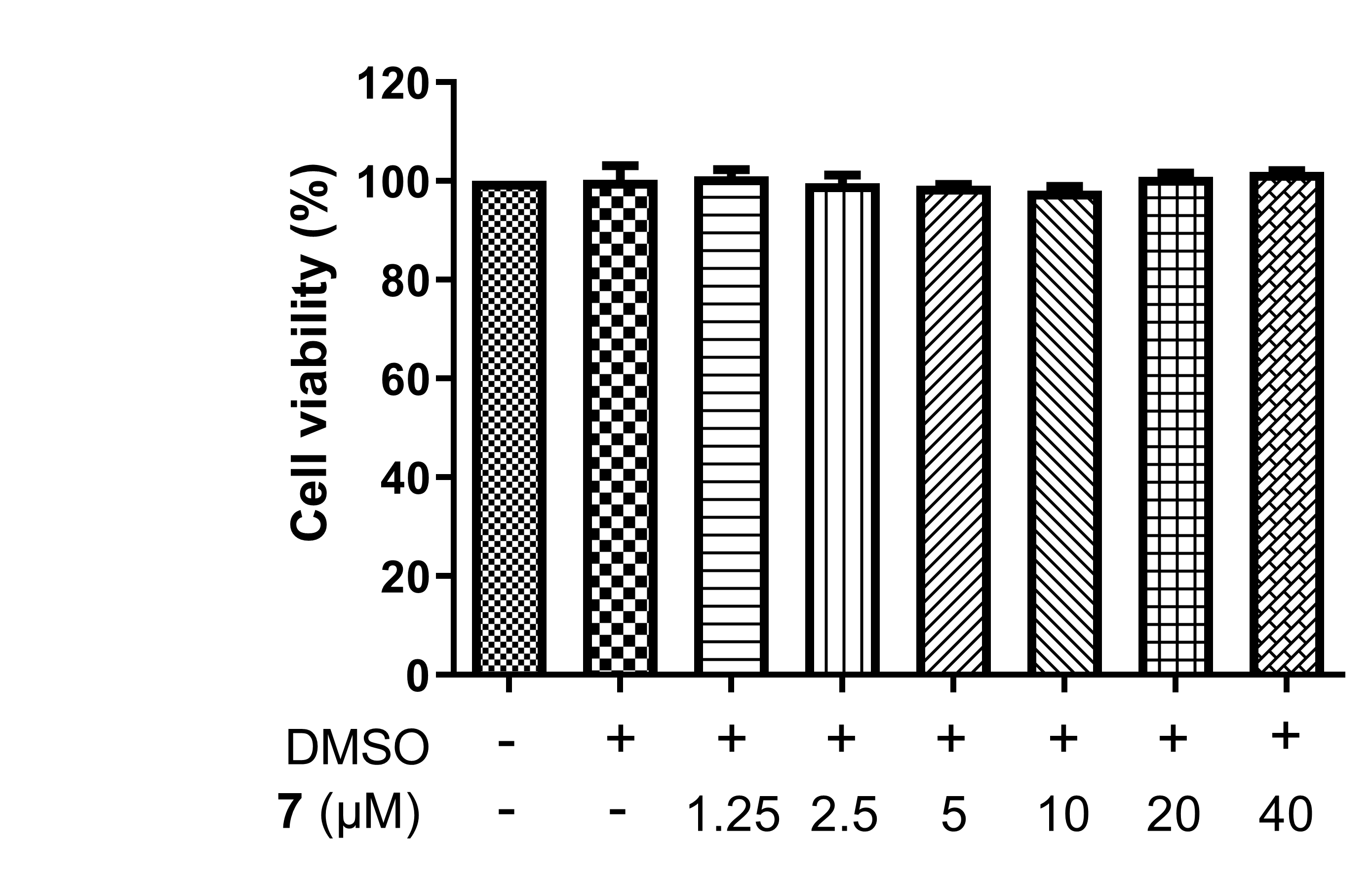

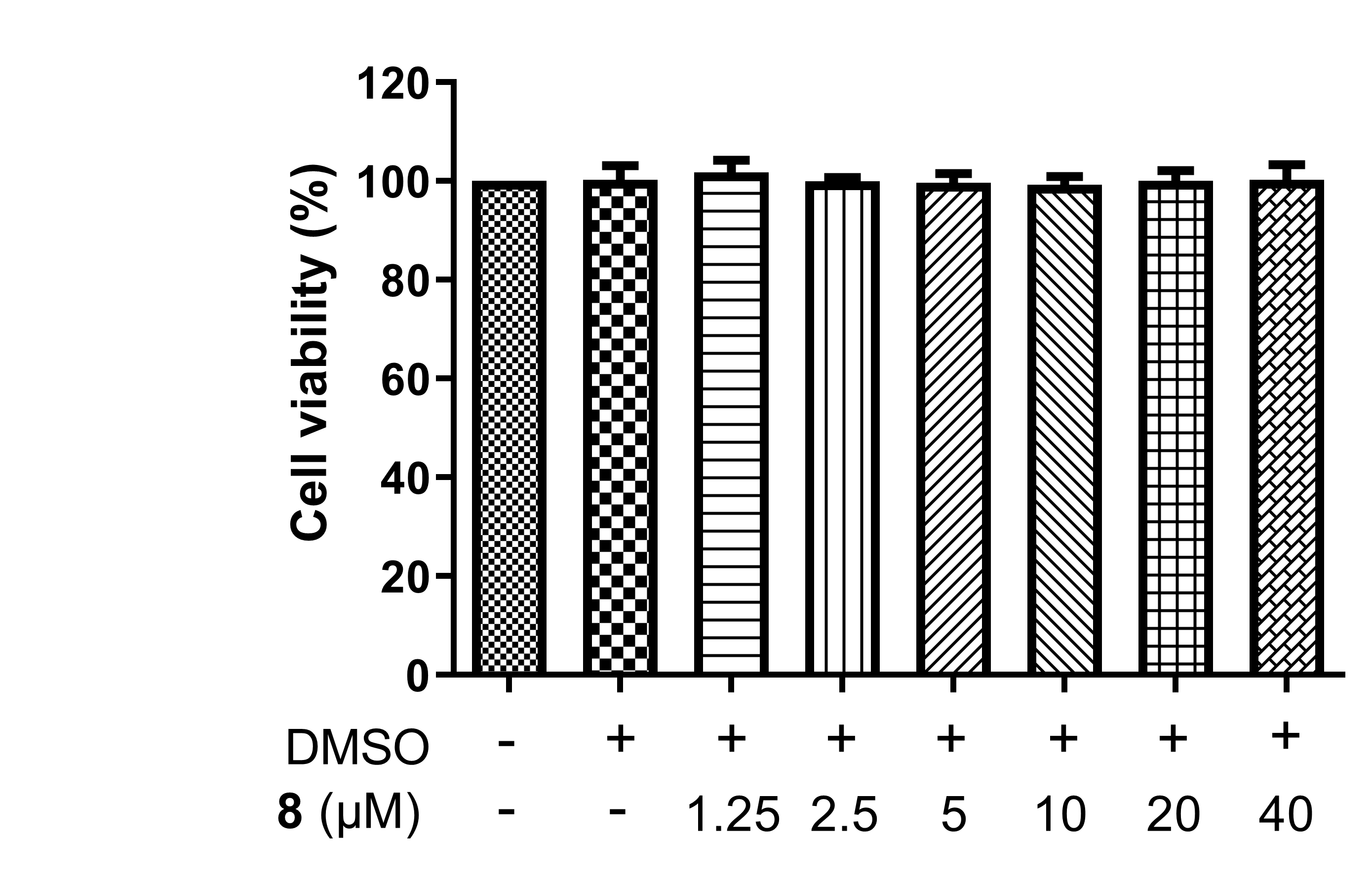

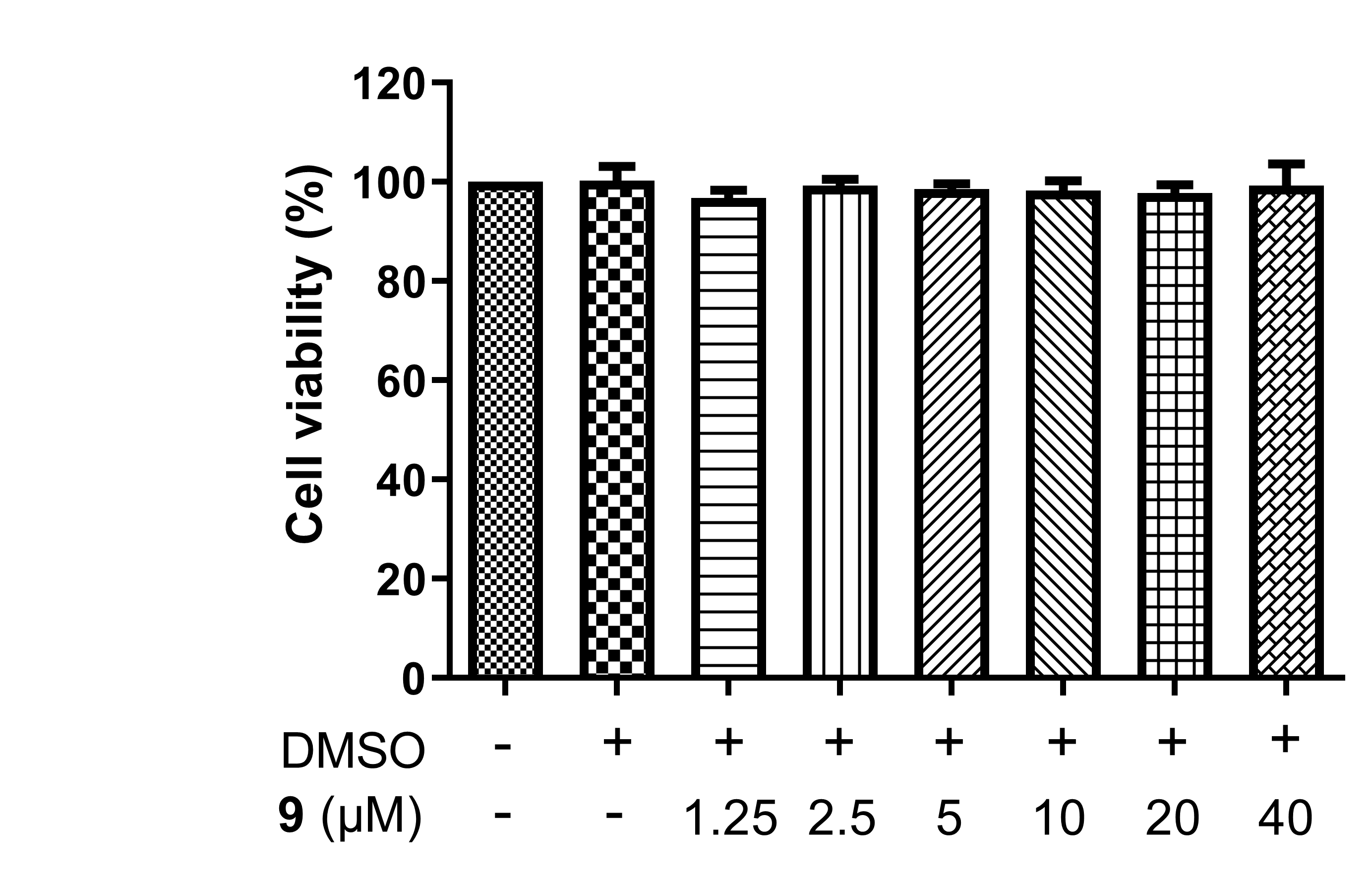


## **Fig. S25** Cytotoxic effect of compounds **1**‒**9** on AML-12 hepatocytes. AML-12 cells were treated with different compounds at indicated concentrations for 24 h. Data are shown as mean ± S.D., n = 3. ***P* < 0.01 vs. DMSO.

## The original Western blots.


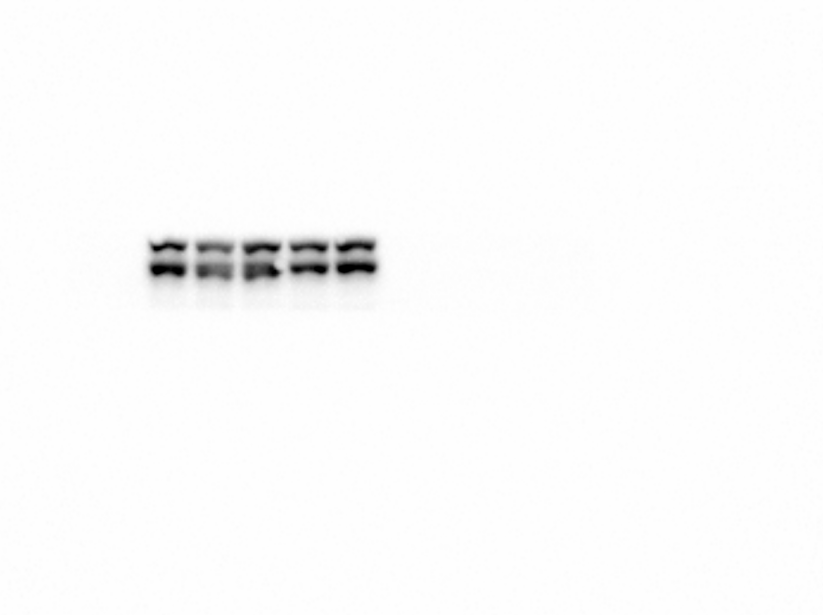


**Fig. S26** The original Western blot of p-AMPK.


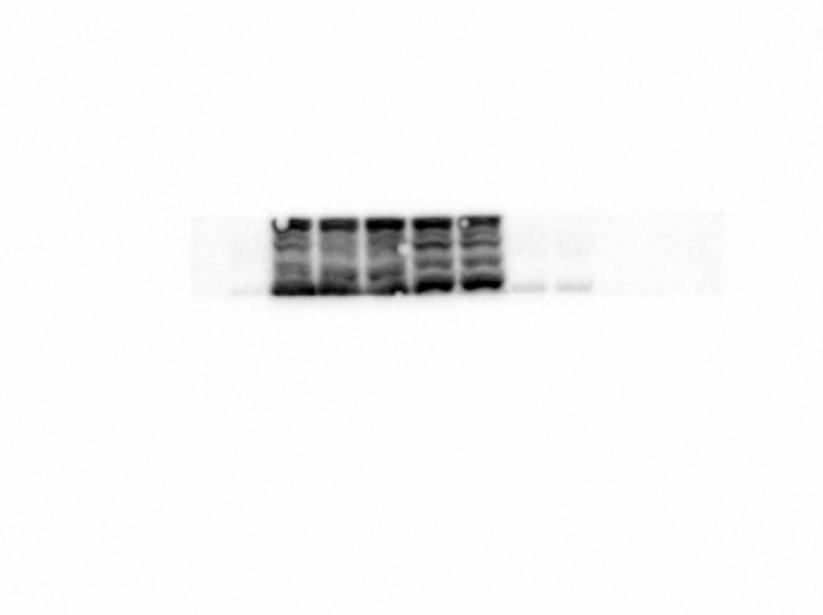


**Fig. S27** The original Western blot of AMPK


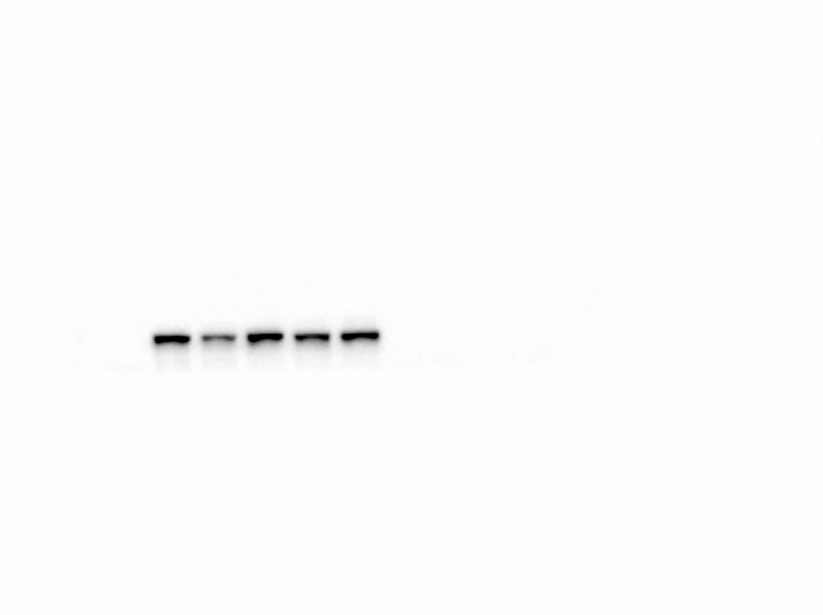


**Fig. S28** The original Western blot of p-ACC.


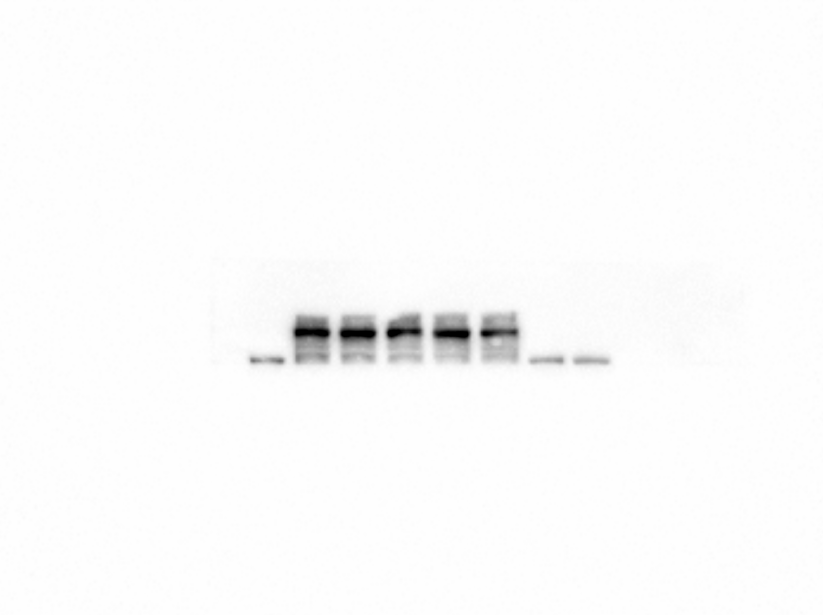


**Fig. S29** The original Western blot of ACC


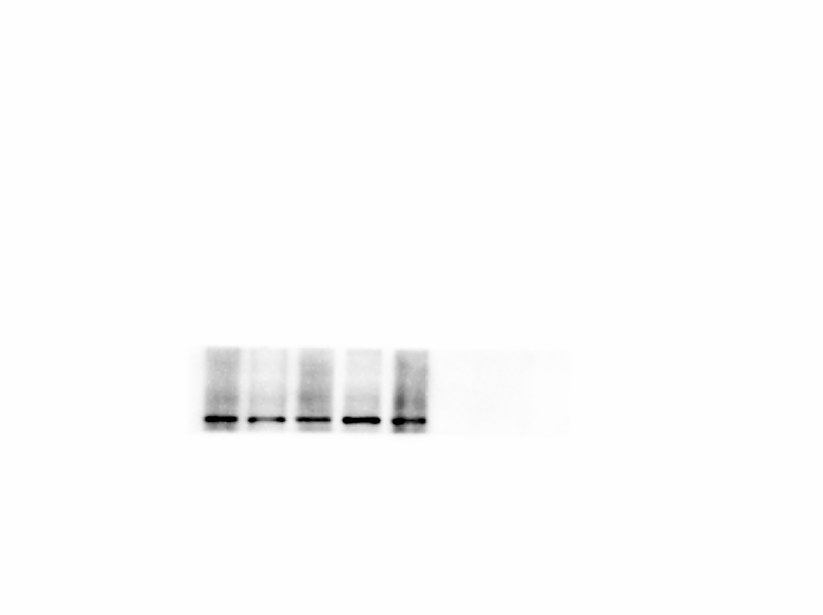


**Fig. S30** The original Western blot of PGC-1α.


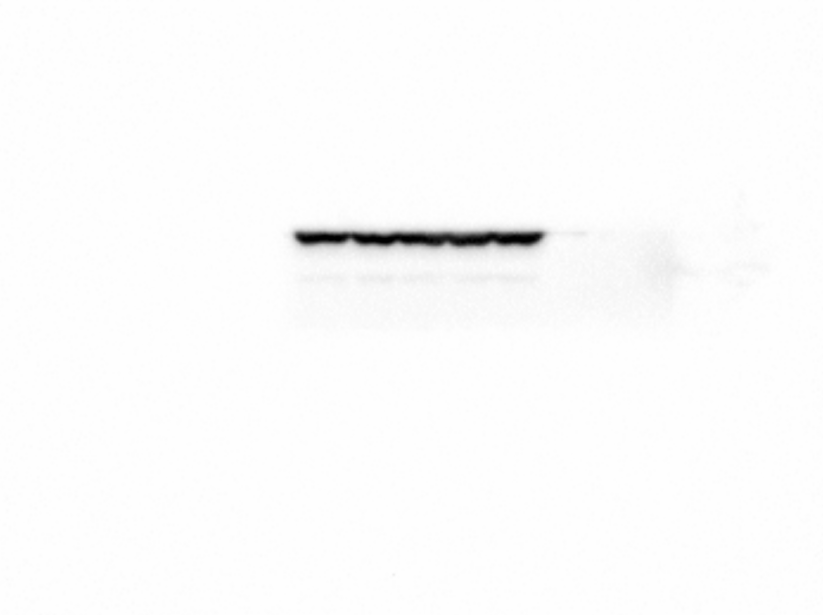


**Fig. S31** The original Western blot of GAPDH.

## **Fig. S32**−**S40** MS, UV, IR, 1D and 2D NMR spectra of diversolanolide A (**1**).


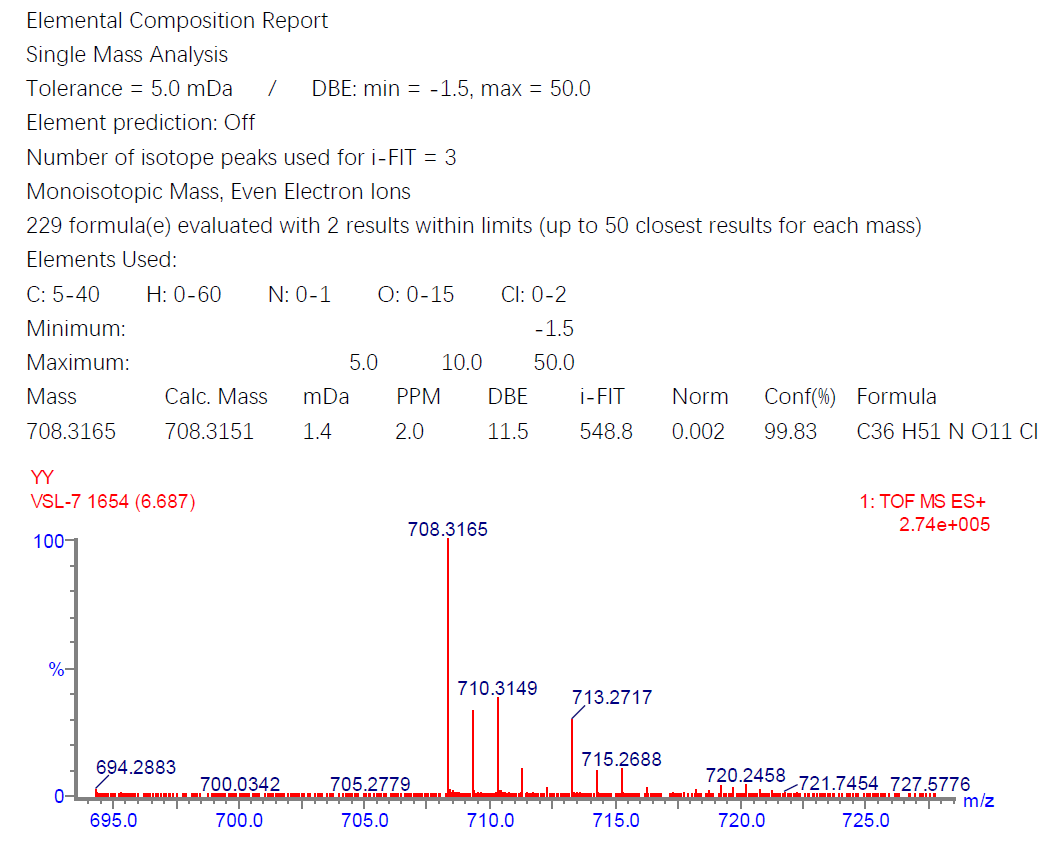


**Fig. S32** HRESIMS spectrum of diversolanolide A (**1**).


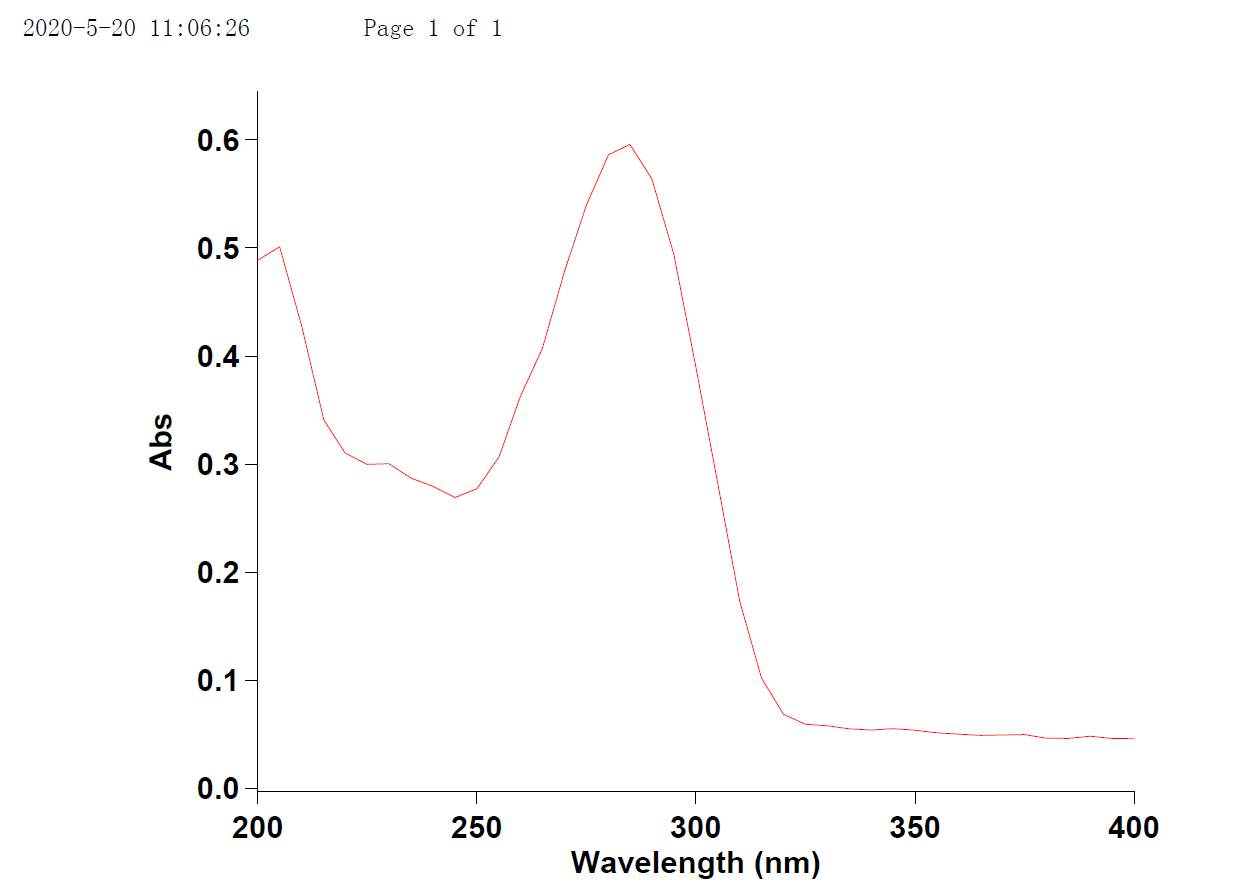


**Fig. S33** UV spectrum of diversolanolide A (**1**).


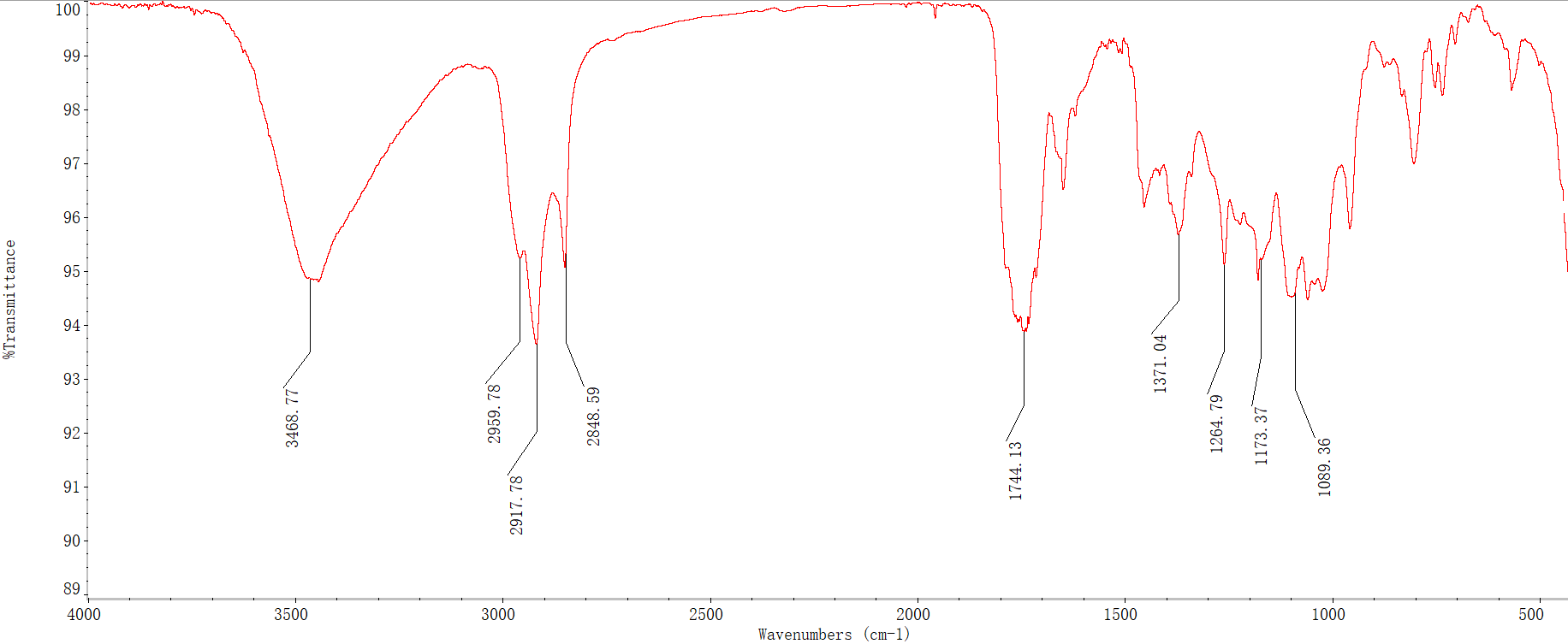


**Fig. S34** IR spectrum of diversolanolide A (**1**).

**Fig. 35** ^1^H NMR (600 MHz) spectrum of diversolanolide A (**1**) in CDCl_3_.

**Fig. S36** ^13^C and DEPT-135 NMR (125 MHz) spectra of diversolanolide A (**1**) in CDCl_3_.

**Fig. S37** ^1^H-^1^H COSY spectrum of diversolanolide A (**1**) in CDCl_3_.

**Fig. S38** HSQC spectrum of diversolanolide A (**1**) in CDCl_3_.


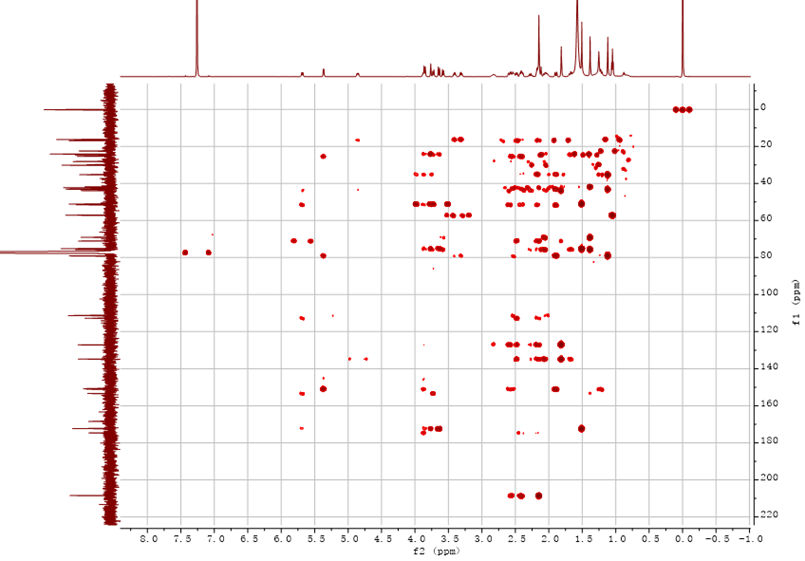


**Fig. S39** HMBC spectrum of diversolanolide A (**1**) in CDCl_3_.

**Fig. S40** ROESY spectrum of diversolanolide A (**1**) in CDCl_3_.

## **Figure S41.**−**Figure S49.** MS, UV, IR, 1D and 2D NMR spectra of diversolanolide B (**2**).


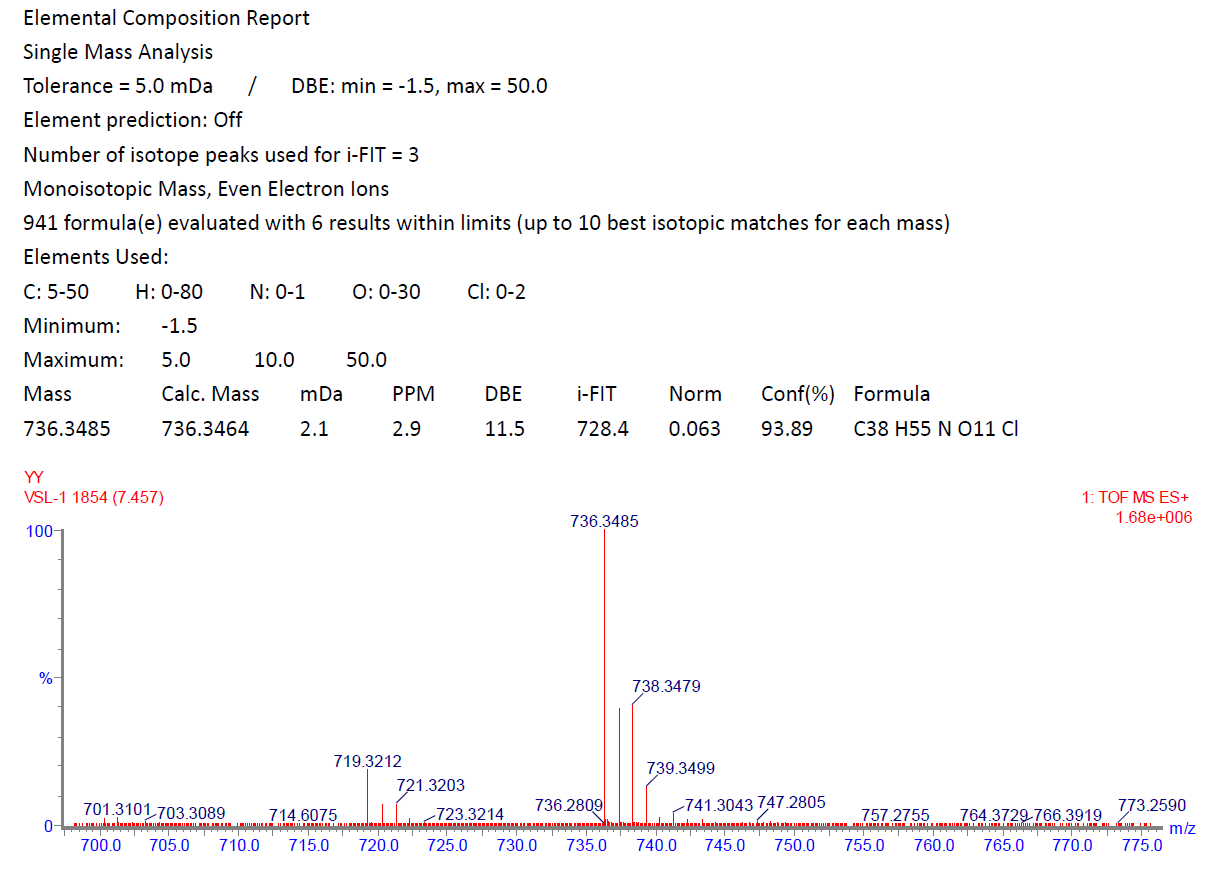


**Fig. S41** HRESIMS spectrum of diversolanolide B (**2**).


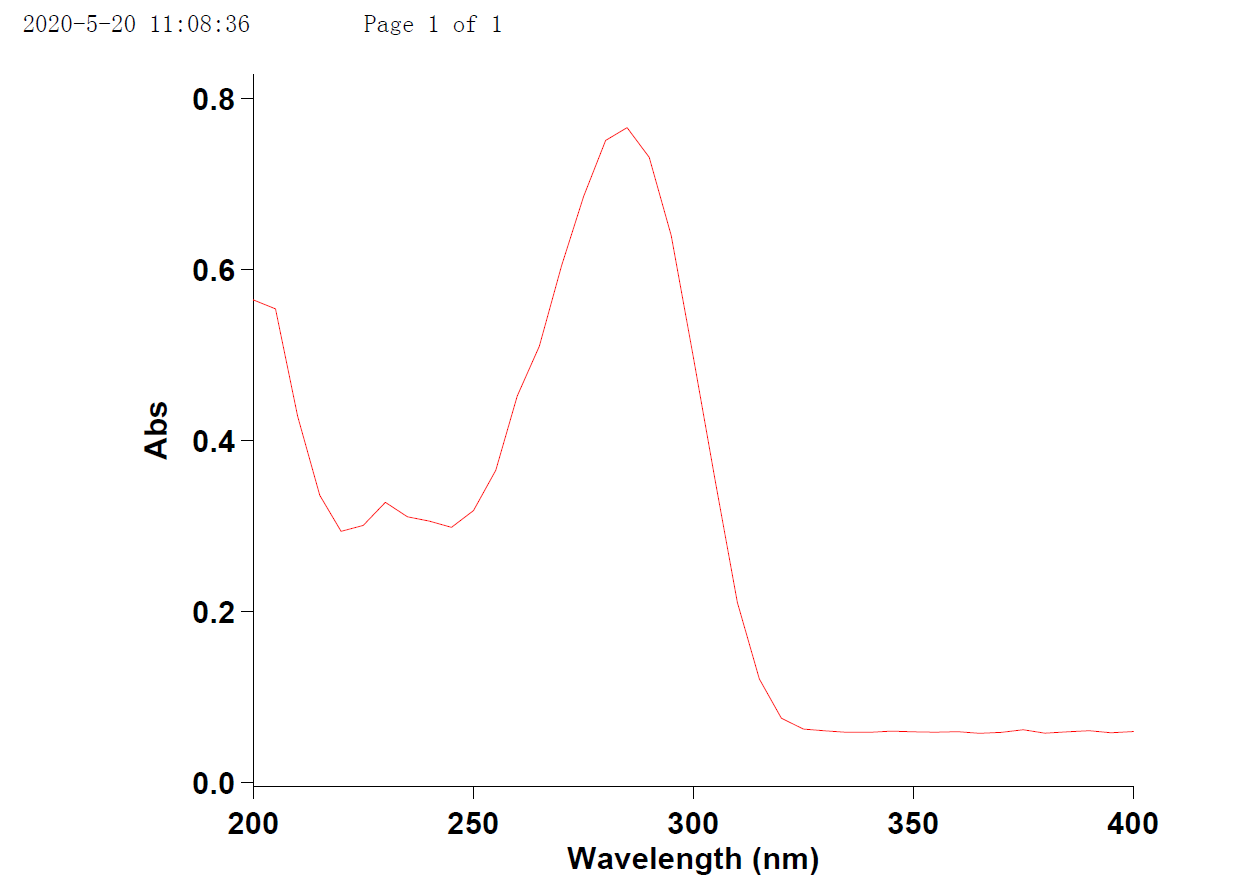


**Fig. S42** UV spectrum of diversolanolide B (**2**).


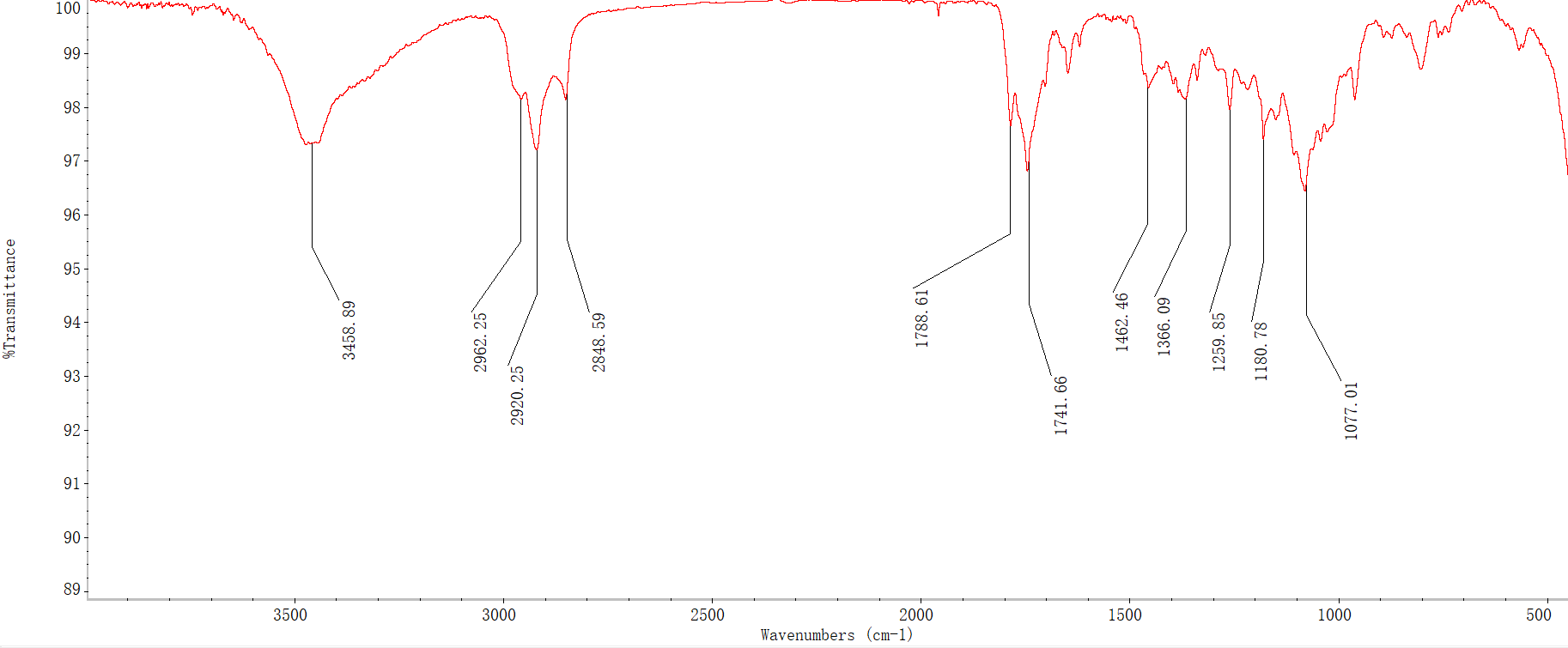


**Fig. S43** IR spectrum of diversolanolide B (**2**).

**Fig. S44** ^1^H NMR (600 MHz) spectrum of diversolanolide B (**2**) in CDCl_3_.

**Fig. S45** ^13^C and DEPT-135 NMR (125 MHz) spectra of diversolanolide B (**2**) in CDCl_3_.

**Fig. S46** ^1^H-^1^H COSY spectrum of diversolanolide B (**2**) in CDCl_3_.

**Fig. S47** HSQC spectrum of diversolanolide B (**2**) in CDCl_3_.

**Fig. S48** HMBC spectrum of diversolanolide B (**2**) in CDCl_3_.

**Fig. S49** NOESY spectrum of diversolanolide B (**2**) in CDCl_3_.

## **Fig. S50**−**58** MS, UV, IR, 1D and 2D NMR spectra of diversolanolide C (**3**).


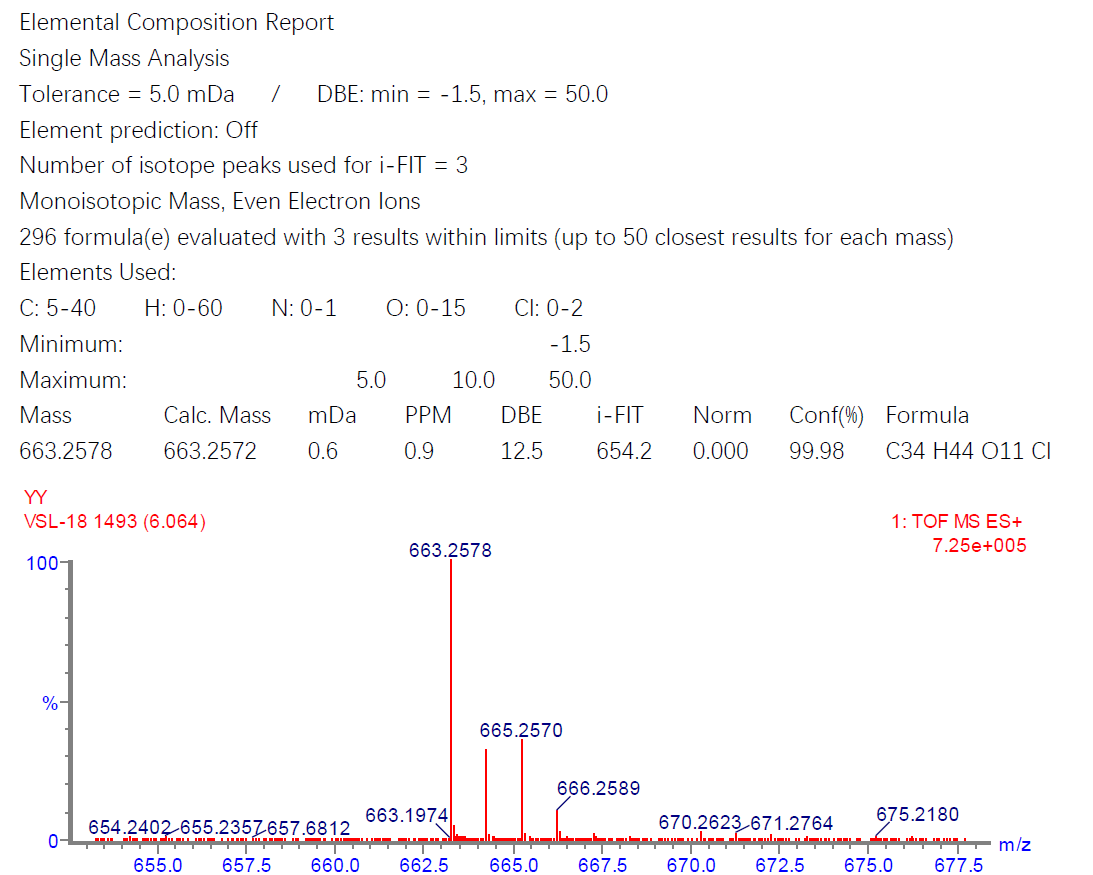


**Fig. S50** HRESIMS spectrum of diversolanolide C (**3**).


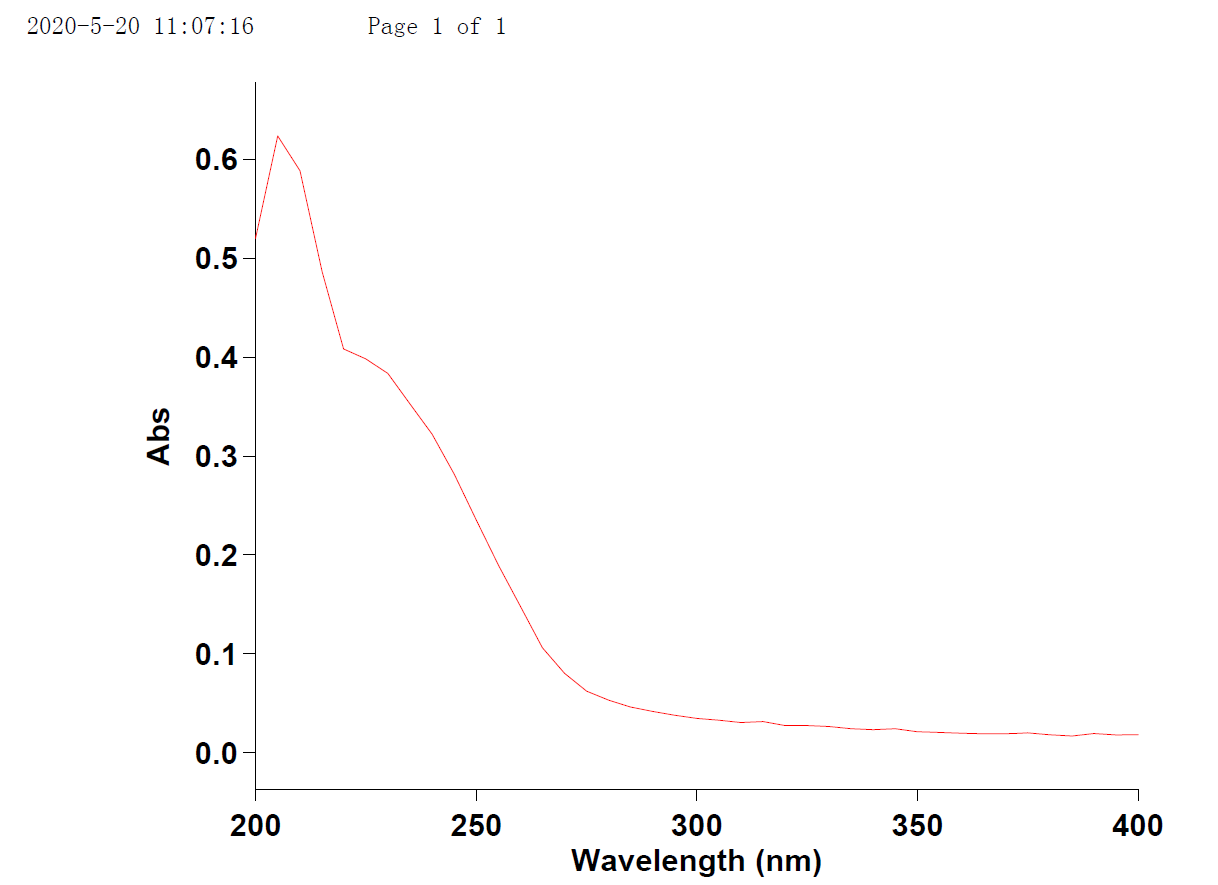


**Fig. S51** UV spectrum of diversolanolide C (**3**).


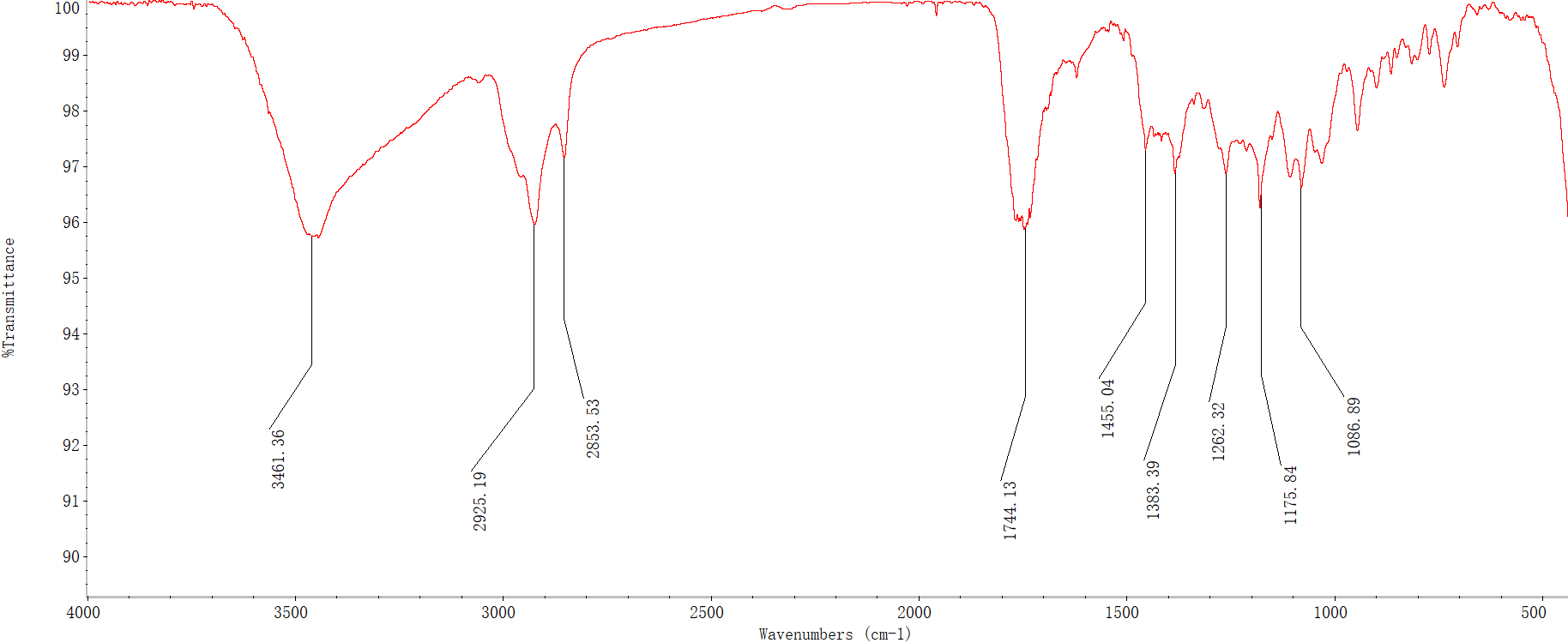


**Fig. S52** IR spectrum of diversolanolide C (**3**).

**Fig. S53** ^1^H NMR (600 MHz) spectrum of diversolanolide C (**3**) in CDCl_3_.

**Fig. S54** ^13^C and DEPT-135 NMR (125 MHz) spectra of diversolanolide C (**3**) in CDCl_3_.

**Fig. S55** ^1^H-^1^H COSY spectrum of diversolanolide C (**3**) in CDCl_3_.

**Fig. S56** HSQC spectrum of diversolanolide C (**3**) in CDCl_3_.

**Fig. S57** HMBC spectrum of diversolanolide C (**3**) in CDCl_3_.

**Fig. S58** NOESY spectrum of diversolanolide C (**3**) in CDCl_3_.


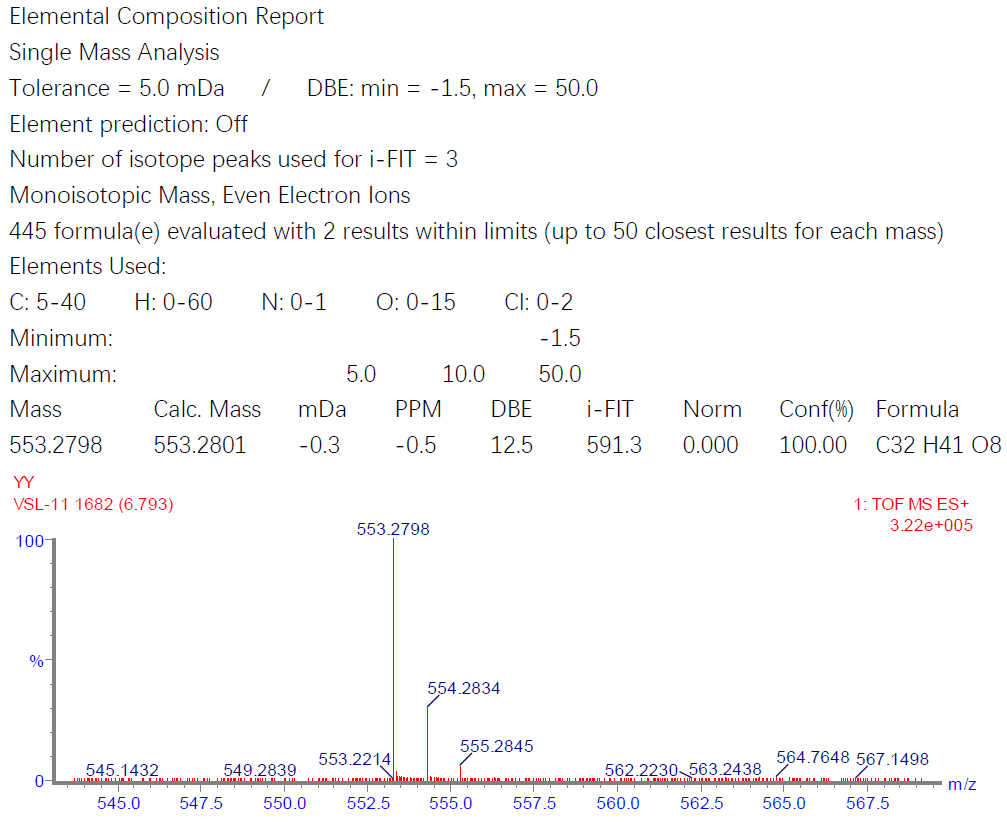


**Fig. S59** HRESIMS spectrum of diversolanolide D (**4**).


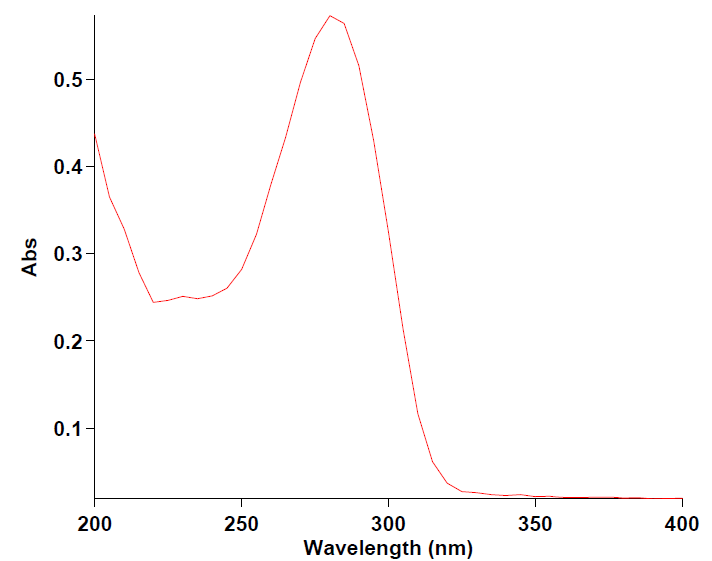


**Fig. S60** UV spectrum of diversolanolide D (**4**).


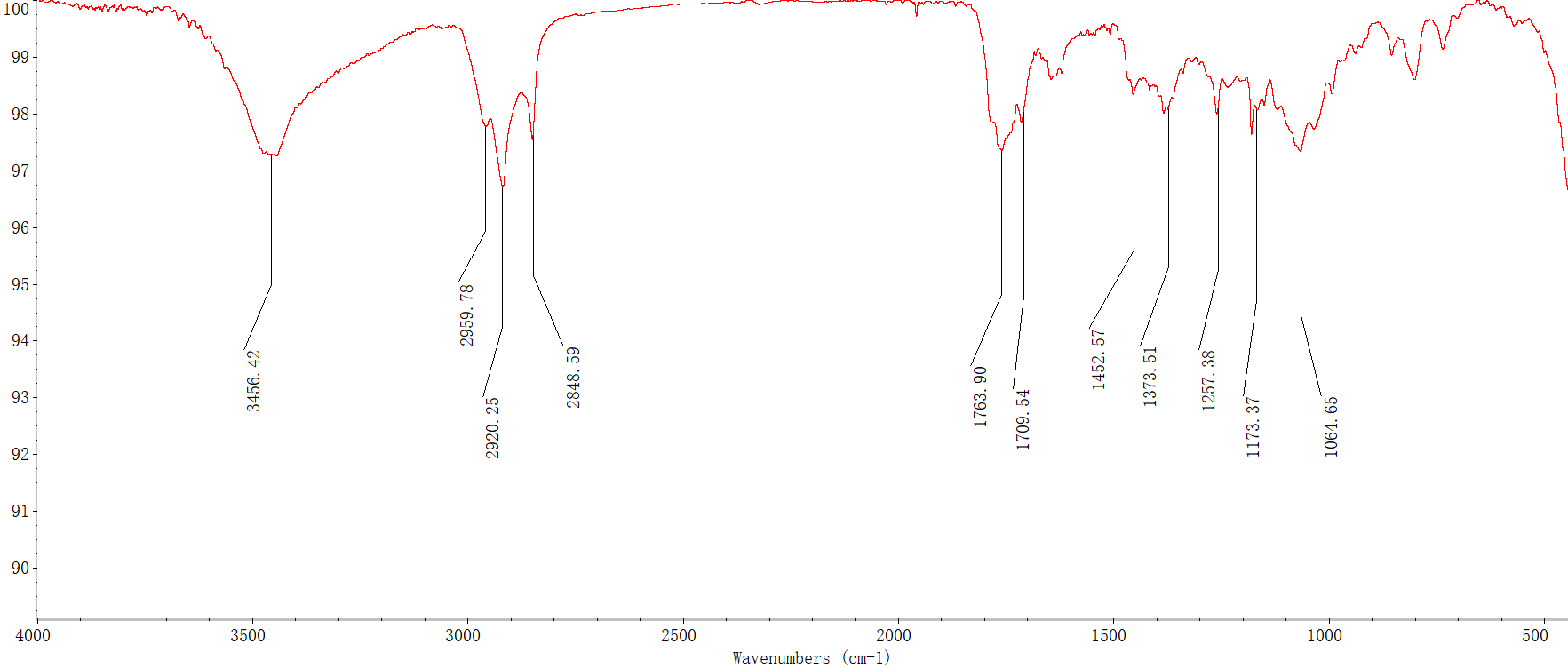


**Fig. S61** IR Spectrum of diversolanolide D (**4**).

**Fig. S62** ^1^H NMR (600 MHz) spectrum of diversolanolide D (**4**) in CDCl_3_.

**Fig. S63** ^13^C and DEPT-135 NMR (125 MHz) spectra of diversolanolide D (**4**) in CDCl_3_.

**Fig. S64** ^1^H-^1^H COSY spectrum of diversolanolide D (**4**) in CDCl_3_.

**Fig. S65** HSQC spectrum of diversolanolide D (**4**) in CDCl_3_.

**Fig. S66** HMBC spectrum of diversolanolide D (**4**) in CDCl_3_.

**Fig. S67** NOESY spectrum of diversolanolide D (**4**) in CDCl_3_.


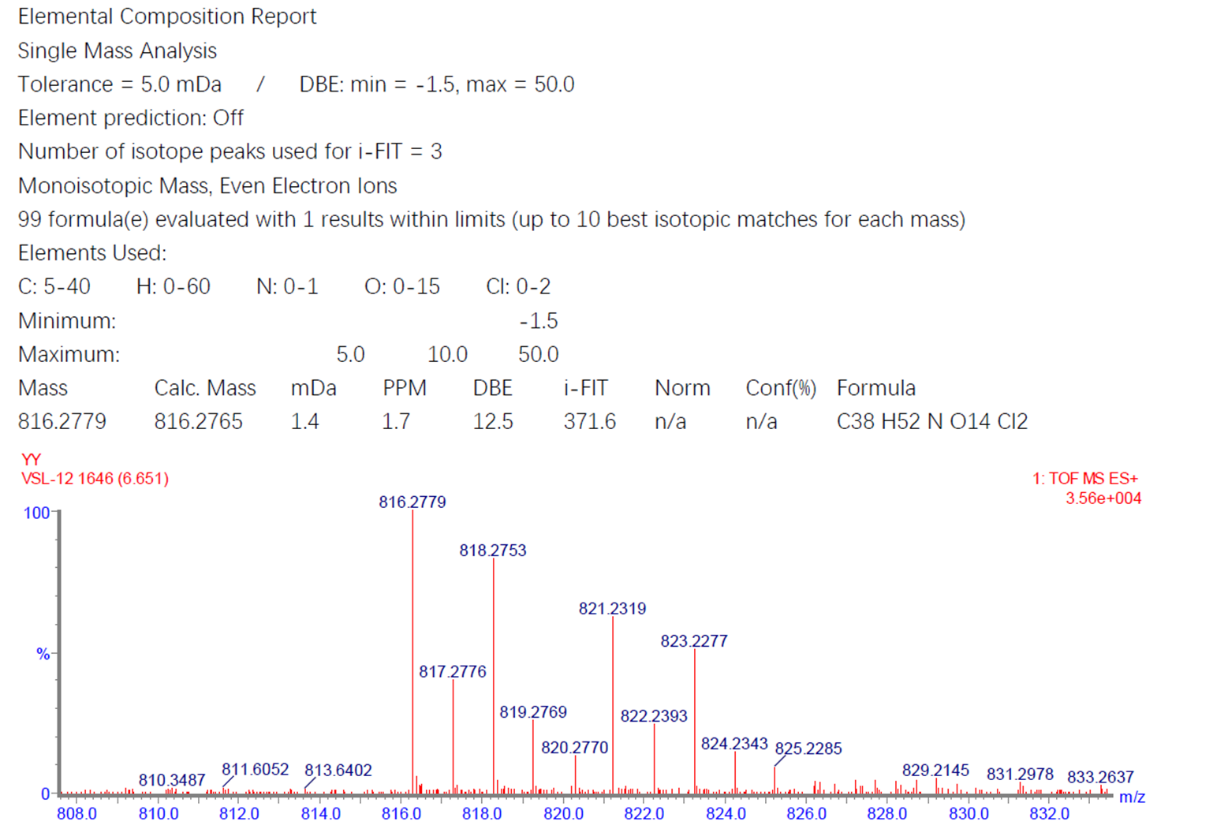


**Fig. S68** HRESIMS spectrum of diversolanolide E (**5**).


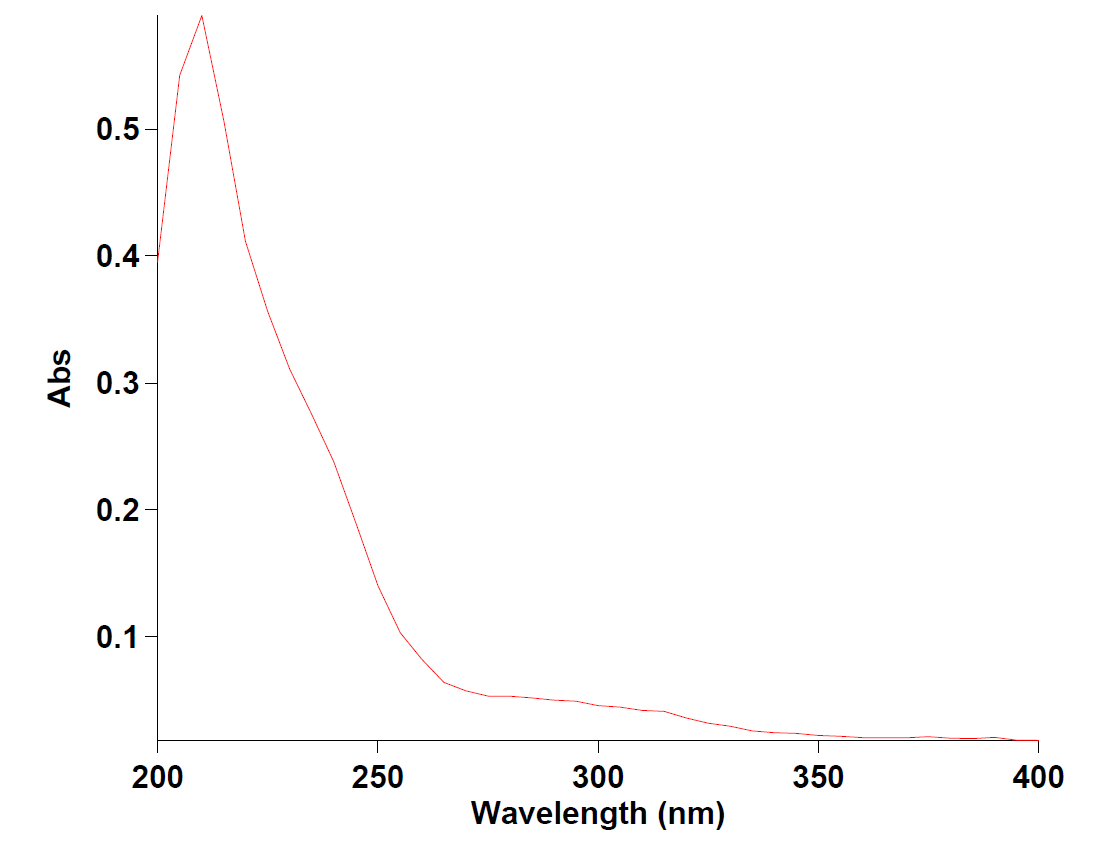


**Fig. S69** UV spectrum of diversolanolide E (**5**).


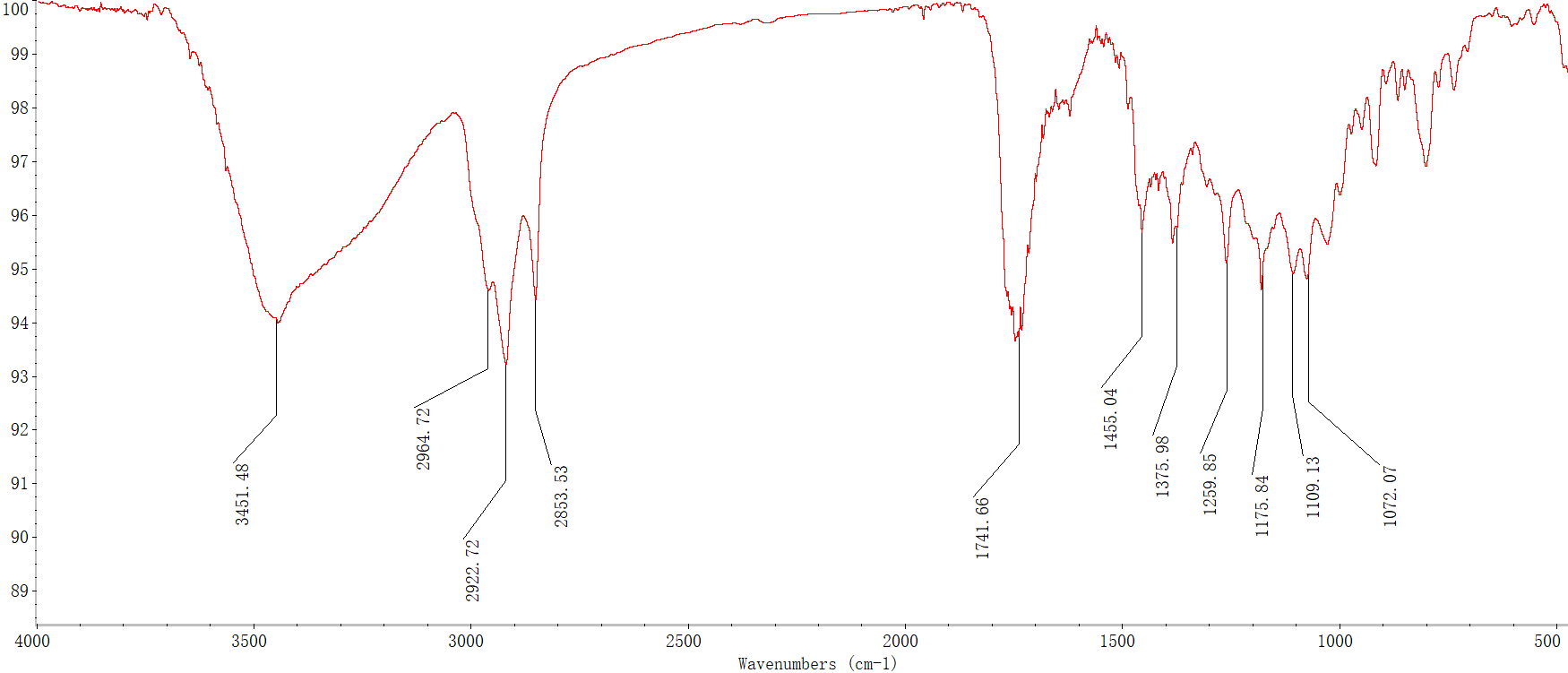


**Fig. S70** IR spectrum of diversolanolide E (**5**).

**Fig. S71** ^1^H NMR (600 MHz) spectrum of diversolanolide E (**5**) in CDCl_3_.

**Fig. S72** ^13^C and DEPT-135 NMR (125 MHz) spectra of diversolanolide E (**5**) in CDCl_3_.

**Fig. S73** ^1^H-^1^H COSY spectrum of diversolanolide E (**5**) in CDCl_3_.

**Fig. S74** HSQC spectrum of diversolanolide E (**5**) in CDCl_3_.

**Fig. S75** HMBC spectrum of diversolanolide E (**5**) in CDCl_3_.

**Fig. S76** NOESY spectrum of diversolanolide E (**5**) in CDCl_3_.


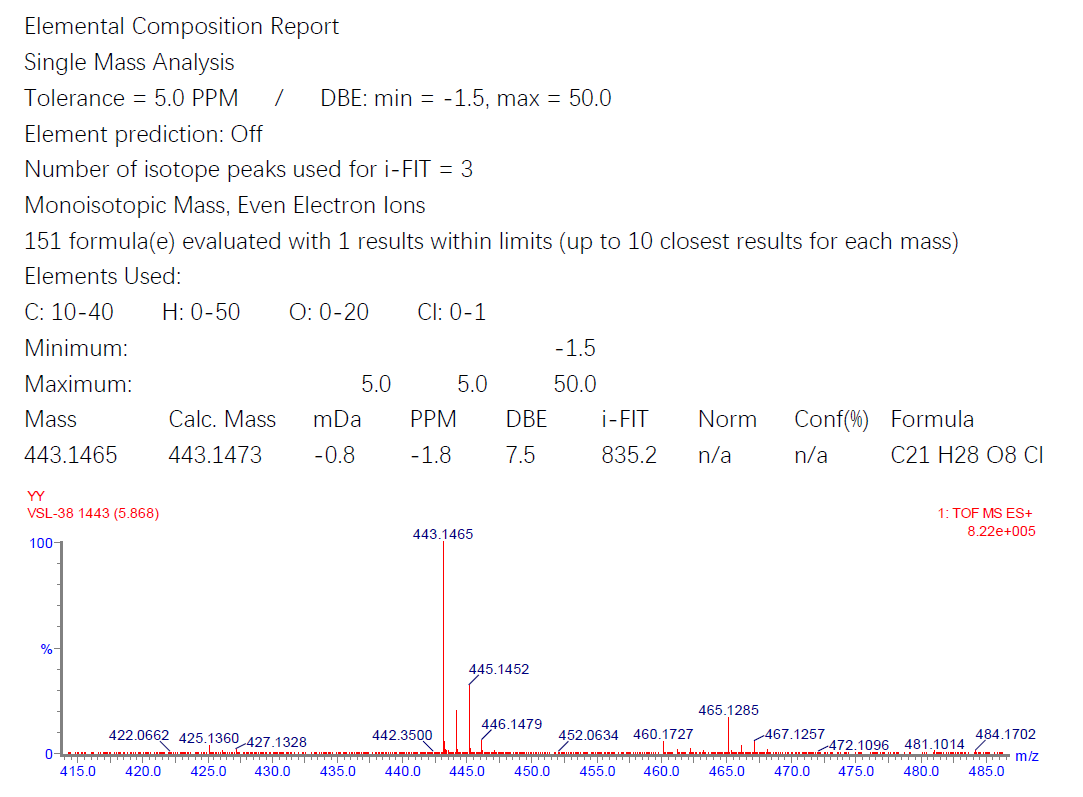


**Fig. S77** HRESIMS spectrum of versolanolide A (**6**).


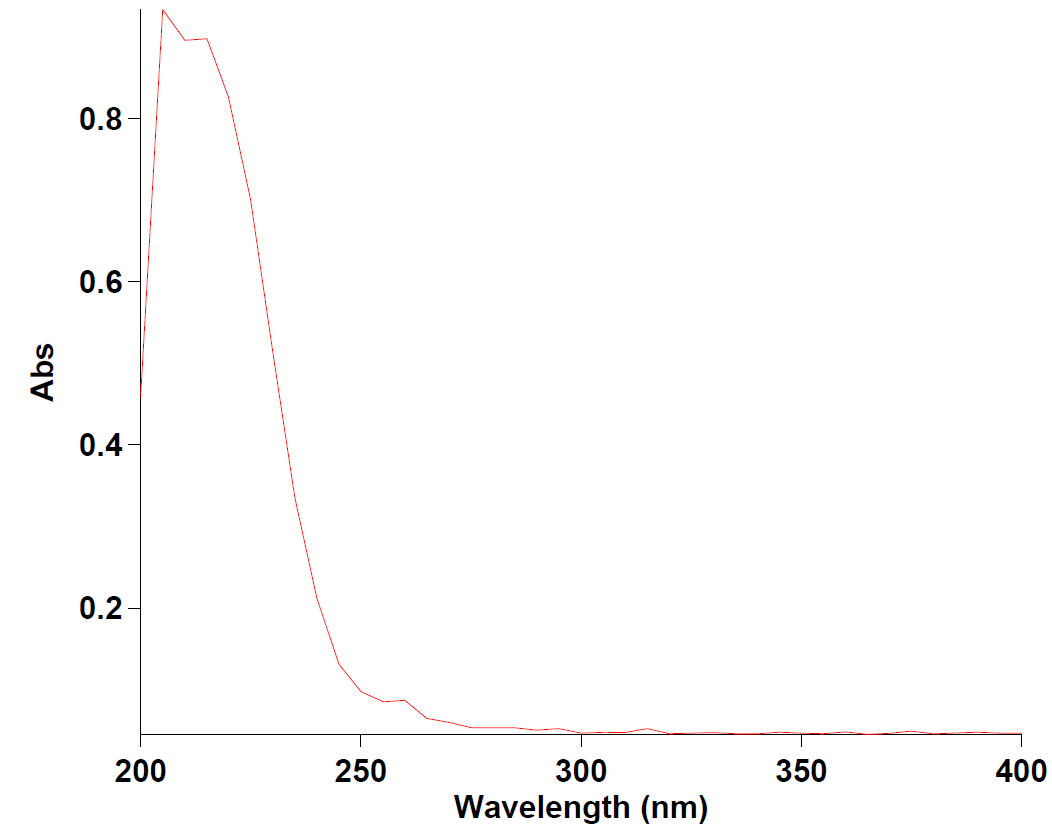


**Fig. S78** UV spectrum of versolanolide A (**6**).


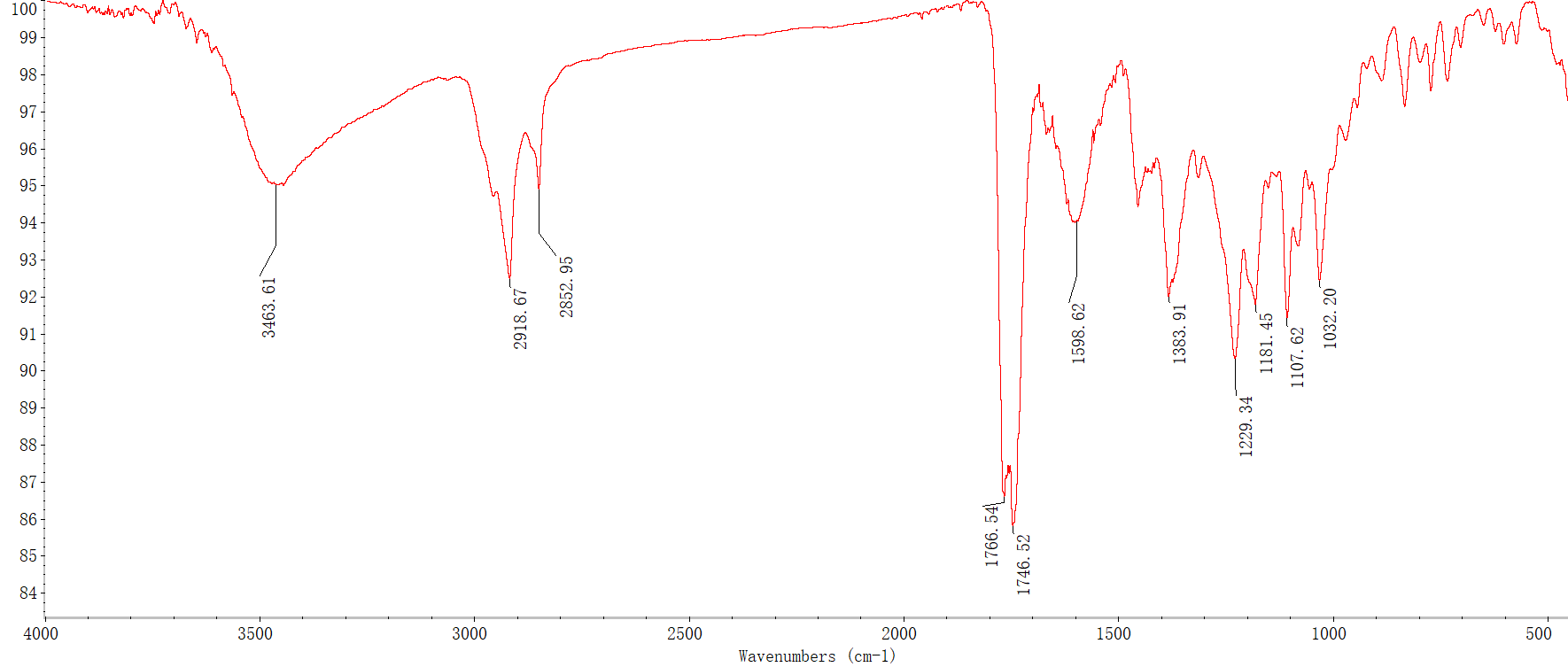


**Fig. S79** IR spectrum of versolanolide A (**6**).

**Fig. S80** ^1^H NMR (600 MHz) spectrum of versolanolide A (**6**) in DMSO-*d*_6_.

**Fig. S81** ^13^C and DEPT-135 NMR (150 MHz) spectra of versolanolide A (**6**) in DMSO-*d*_6_.

**Fig. S82** ^1^H-^1^H COSY spectrum of versolanolide A (**6**) in DMSO-*d*_6_.

**Fig. S83** HSQC spectrum of versolanolide A (**6**) in DMSO-*d*_6_.

__

**Fig. S84** HMBC spectrum of versolanolide A (**6**) in DMSO-*d*_6_.

**Fig. S85** NOESY spectrum of versolanolide A (**6**) in DMSO-*d*_6_.


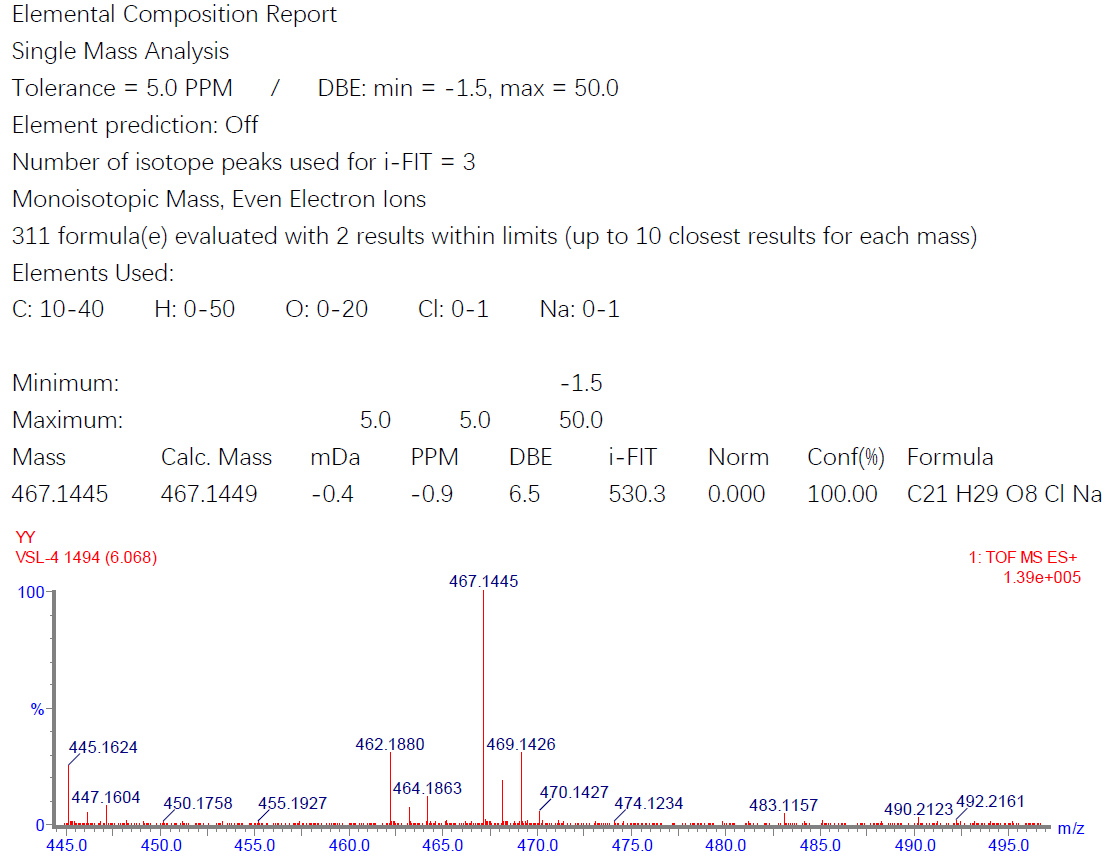


**Fig. S86** HRESIMS spectrum of versolanolide B (**7**).


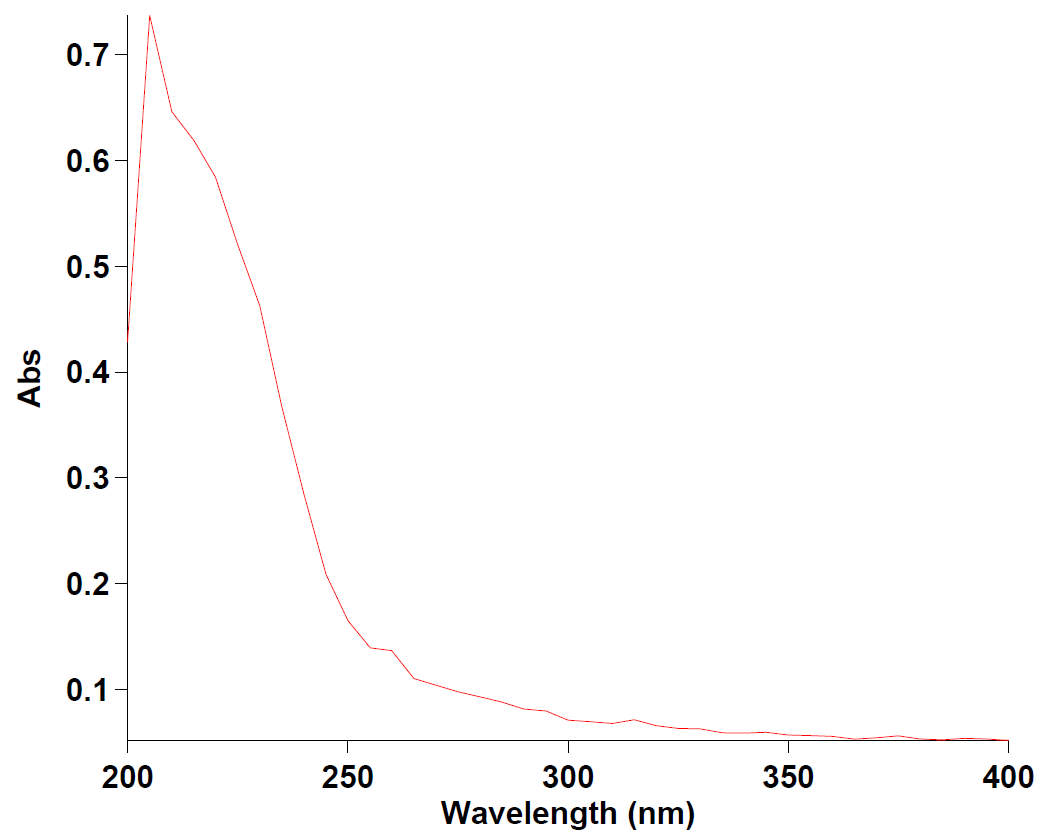


**Fig. S87** UV spectrum of versolanolide B (**7**).


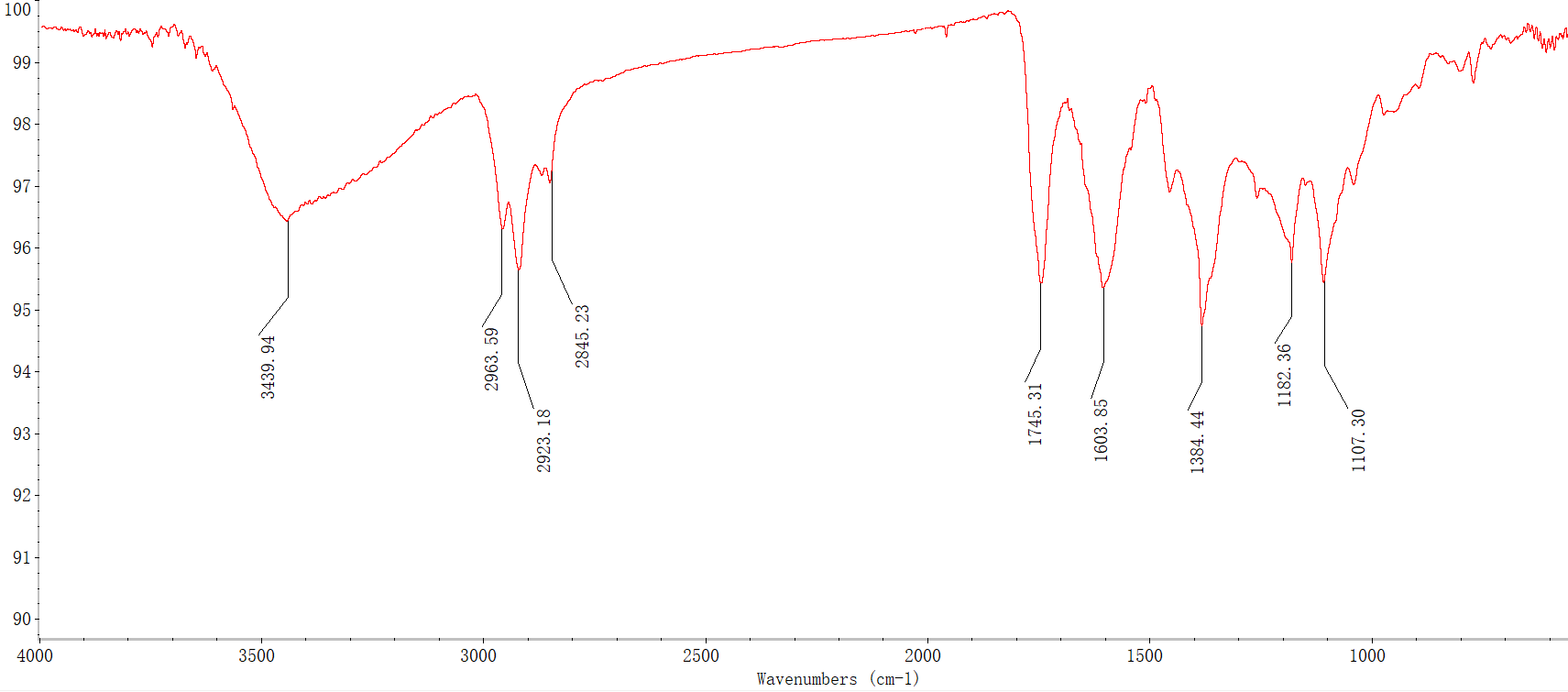


**Fig. S88** IR spectrum of versolanolide B (**7**).

**Fig. S89** ^1^H NMR (600 MHz) spectrum of versolanolide B (**7**) in CDCl_3_.

**Fig. S90** ^13^C and DEPT-135 NMR (125 MHz) spectra of versolanolide B (**7**) in CDCl_3_.

**Fig. S91** ^1^H-^1^H COSY spectrum of versolanolide B (**7**) in CDCl_3_.

**Fig. S92** HSQC spectrum of versolanolide B (**7**) in CDCl_3_.

**Fig. S93** HMBC spectrum of versolanolide B (**7**) in CDCl_3_.

**Fig. S94** NOESY spectrum of versolanolide B (**7**) in CDCl_3_.

_
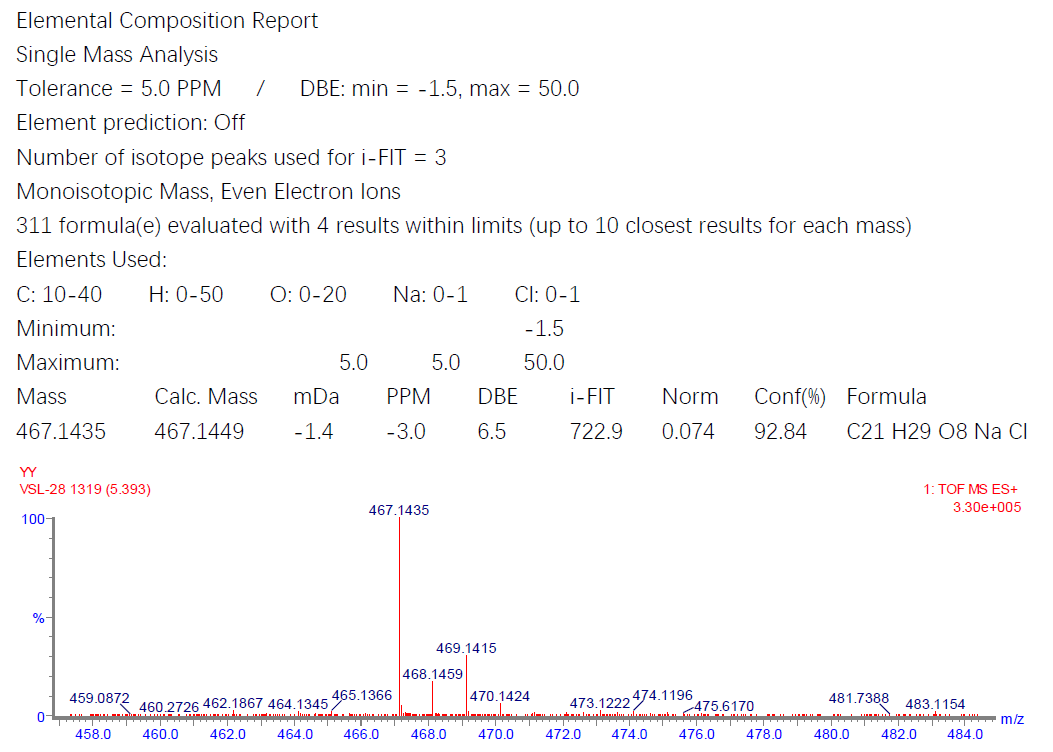
_

**Fig. S95** HRESIMS spectrum of versolanolide C (**8**).


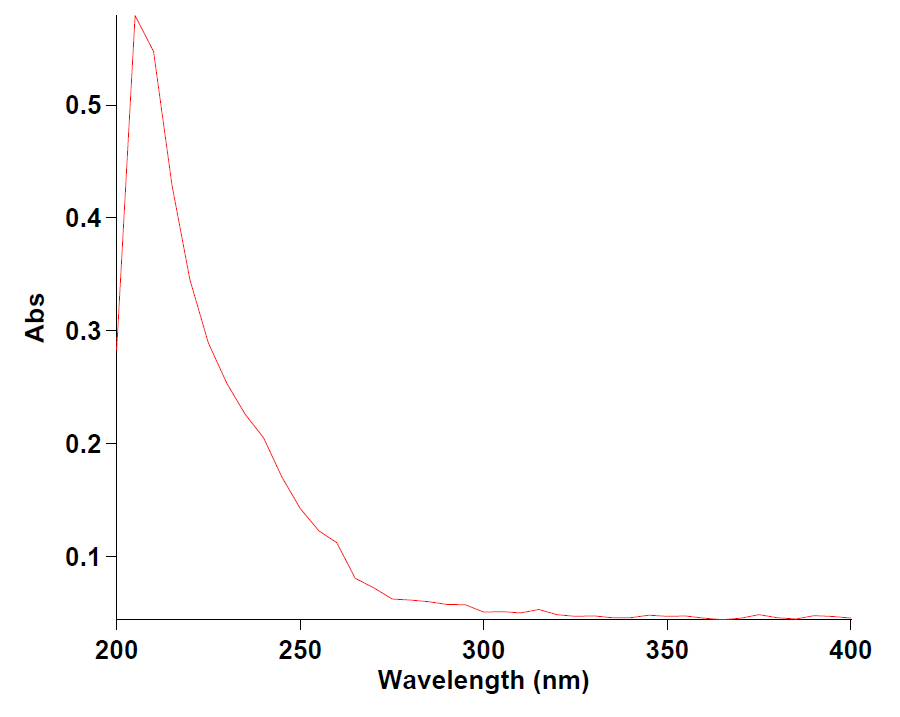


**Fig. S96** UV spectrum of versolanolide C (**8**).

_
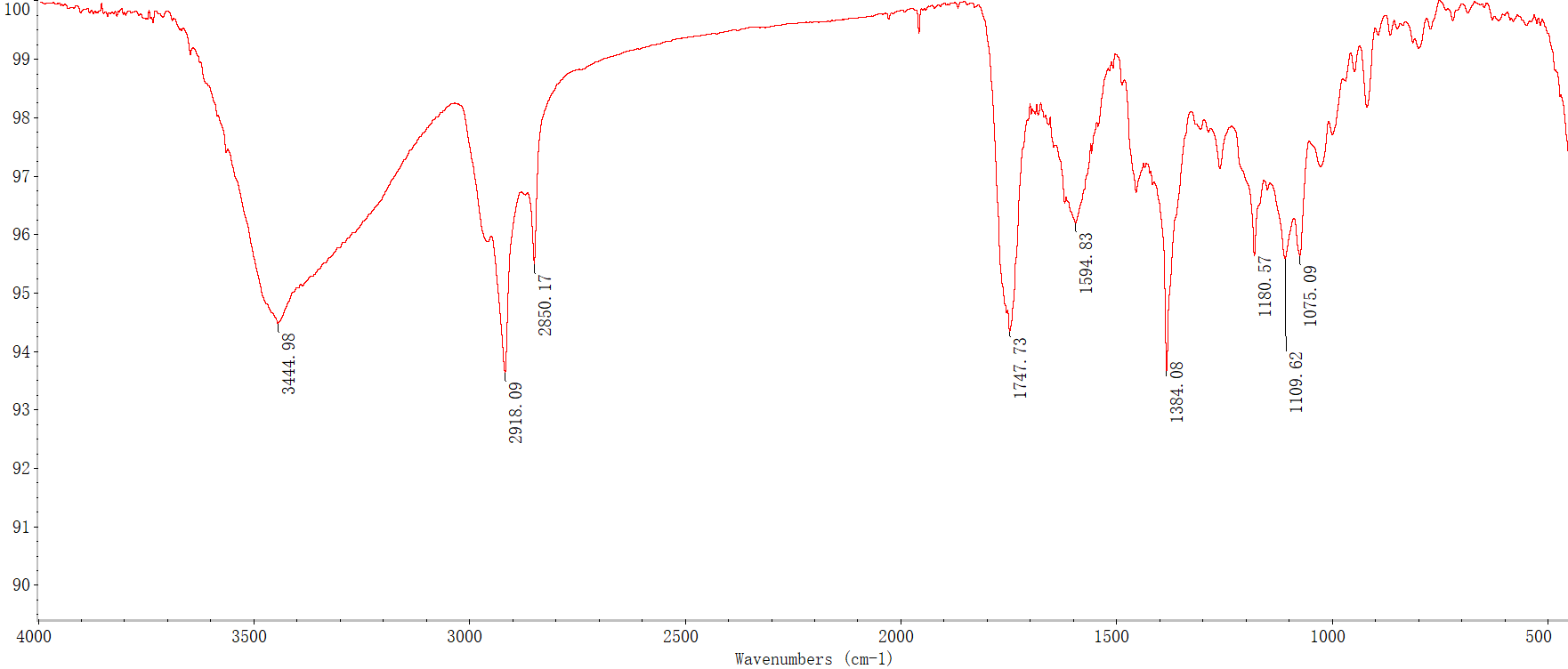
_

**Fig. S97** IR spectrum of versolanolide C (**8**).

__

**Fig. S98** ^1^H NMR (600 MHz) spectrum of versolanolide C (**8**) in CDCl_3_.

__

**Fig. S99** ^13^C and DEPT-135 NMR (125 MHz) spectra of versolanolide C (**8**) in CDCl_3_.

**Fig. S100** ^1^H-^1^H COSY spectrum of versolanolide C (**8**) in CDCl_3_.

**Fig. S101** HSQC spectrum of versolanolide C (**8**) in CDCl_3_.

**Fig. S102** HMBC spectrum of versolanolide C (**8**) in CDCl_3_.

**Fig. S103** NOESY spectrum of versolanolide C (**8**) in CDCl_3_.


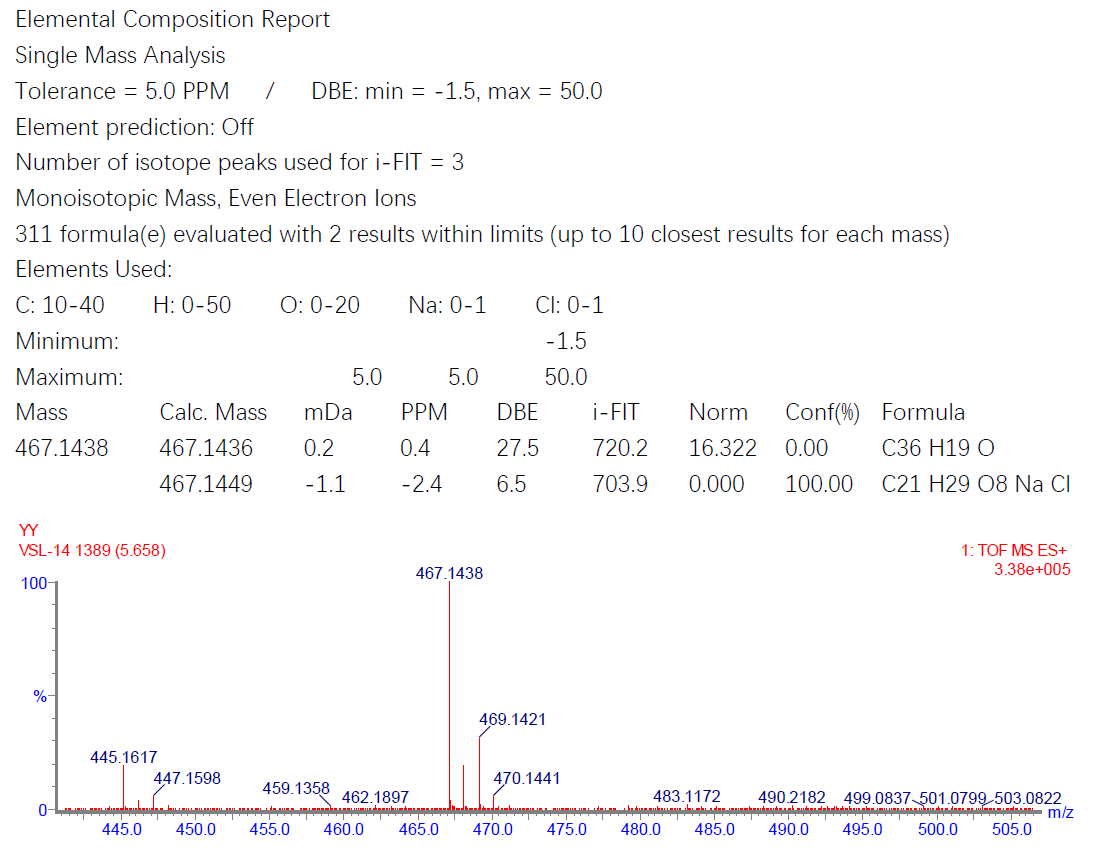


**Fig. S104** HRESIMS spectrum of versolanolide D (**9**).


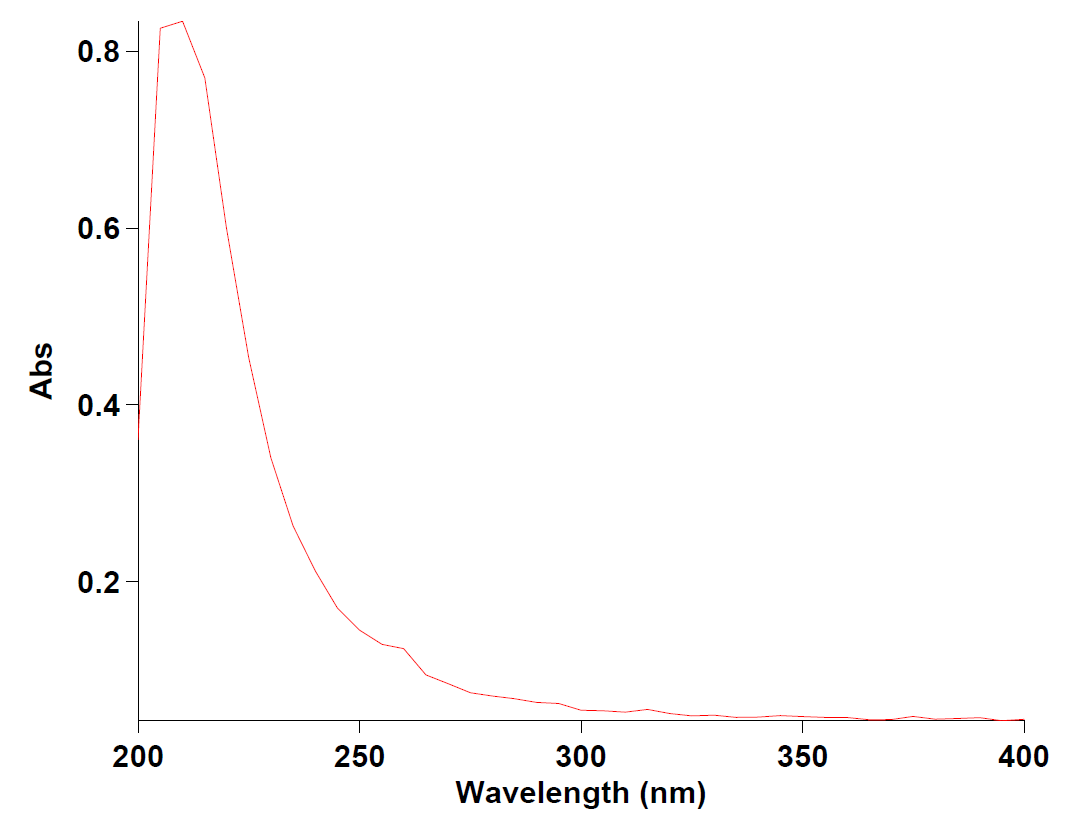


**Fig. S105** UV spectrum of versolanolide D (**9**).


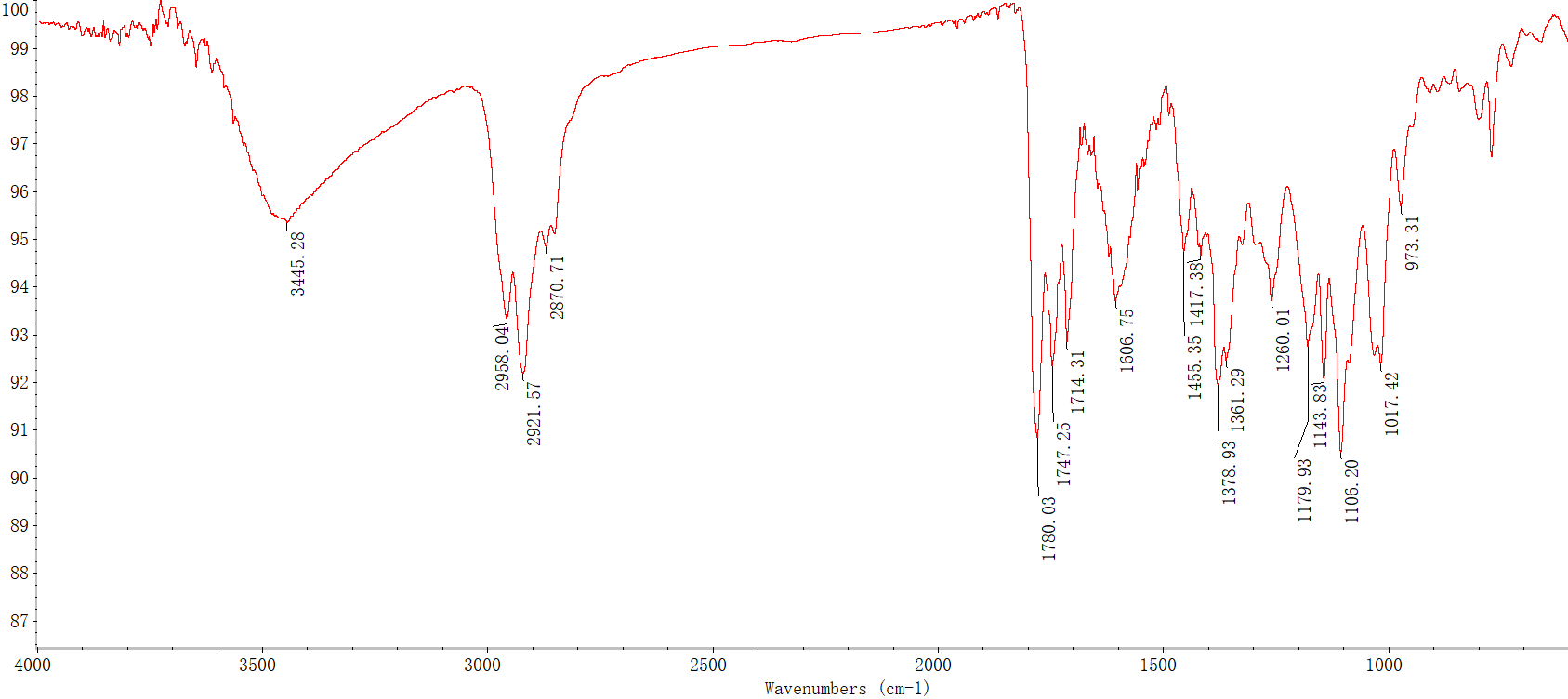


**Fig. S106** IR spectrum of versolanolide D (**9**).

**Fig. S107** ^1^H NMR (600 MHz) spectrum of versolanolide D (**9**) in CDCl_3_.

**Fig. S108** ^13^C and DEPT-135 NMR (125 MHz) spectra of versolanolide D (**9**) in CDCl_3_.

**Fig. S109** ^1^H-^1^H COSY spectrum of versolanolide D (**9**) in CDCl_3_.

**Fig. S110** HSQC spectrum of versolanolide D (**9**) in CDCl_3_.

**Fig. S111** HMBC spectrum of versolanolide D (**9**) in CDCl_3_.

**Fig. S112** NOESY spectrum of versolanolide D (**9**) in CDCl_3_.
